# Exosomal CMTM4 Induces Immunosuppressive Macrophages to Promote Ovarian Cancer Progression and Attenuate Anti‐PD‐1 Immunotherapy

**DOI:** 10.1002/advs.202504436

**Published:** 2025-05-28

**Authors:** Bo Yin, Jianyi Ding, Jie Liu, Haoran Hu, Yashi Zhu, Meiqin Yang, Huijuan Zhou, Baoyou Huang, Tiefeng Huang, Mengjie Li, Yinyan He, Ang Li, Lingfei Han

**Affiliations:** ^1^ Department of Gynecology Shanghai Key Laboratory of Maternal Fetal Medicine Shanghai Institute of Maternal‐Fetal Medicine and Gynecologic Oncology Shanghai First Maternity and Infant Hospital School of Medicine Tongji University Shanghai 200092 China; ^2^ Department of Gynecology Shanghai Tenth People's Hospital Tongji University School of Medicine Shanghai China; ^3^ School of life science and technology Tongji University Shanghai China; ^4^ Department of Gynecology The First Affiliated Hospital of Wenzhou Medical University Wenzhou Zhejiang 325027 China

**Keywords:** CMTM4, exosomal CMTM4, macrophage polarization, ovarian cancer, tumor‐associated macrophages

## Abstract

Exosomes shape the tumor microenvironment (TME) by modulating tumor‐associated macrophages (TAMs) and promoting ovarian cancer (OC) progression. This study reveals that exosomal CKLF Like MARVEL Transmembrane Domain Containing 4 (CMTM4) enhances OC malignancy and orchestrates immune evasion. Excessive macrophage infiltration in the TME, particularly in the presence of CMTM4, is strongly associated with poor prognosis. Within the TME, exosomal CMTM4 is actively internalized by macrophages, promoting M2 polarization and subsequently initiating immunosuppressive signaling. Exosomal CMTM4 activates the NF‐κB pathway in TAMs, suppressing immune function through enhanced secretion of cytokines, including TGF‐β1 and CXCL12, while simultaneously upregulating intercellular adhesion molecule‐1 (ICAM1) expression to further promote M2 polarization and facilitate cancer metastasis. Depletion of CMTM4 increases sensitivity to anti‐PD‐1 therapy by reversing immunosuppression. Notably, eltrombopag is identified as a CMTM4 inhibitor that attenuates OC progression in vivo and modulates the tumor immune microenvironment, synergizing with PD‐1 blockade immunotherapy to enhance therapeutic efficacy. The exosomal CMTM4—ICAM1—CD206 axis exacerbates disease risk in patients with OC. Collectively, the study highlights the critical role of tumor‐derived exosomal CMTM4 in immune suppression, emphasizing its potential as both a prognostic biomarker and a therapeutic target in OC immunotherapy.

## Graphical abstract

In ovarian tumors with high CMTM4 expression, exosomes transfer CMTM4 to macrophages, upregulating ICAM1 and promoting M2 polarization via p65 nuclear translocation. This suppresses immunity by increasing PD‐1 and Tregs while reducing CD8^+^ T cells. Targeting CMTM4 with drugs or RNA interference restores anti‐tumor immunity and improves prognosis.

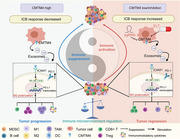

## 1 Introduction

Ovarian cancer (OC) remains one of the most lethal gynecological malignancies, primarily due to its aggressive nature and late‐stage diagnosis.^[^ ^1^ ^]^ Despite advances in surgical techniques and chemotherapy, the overall prognosis for patients with advanced OC remains poor, as the disease often recurs after initial treatment. Current therapeutic strategies primarily involve cytoreductive surgery and chemotherapy, with platinum‐based regimens as the standard of care. However, the effectiveness of these treatments is often limited by the development of chemoresistance and the complexity of the tumor microenvironment (TME), which contributes to immune evasion and cancer progression.^[^ ^2^, ^3^ ^]^

The TME comprises various stromal and immune cells that support tumor growth and metastasis.^[^ ^4^, ^5^ ^]^ Among these, tumor‐associated macrophages (TAMs) play a central role in modulating immune responses and promoting tumor progression. The polarization of TAMs toward an immunosuppressive M2 phenotype is a key factor in establishing a pro‐tumoral environment that fosters metastasis and immune evasion.^[^ ^6^, ^7^, ^8^ ^]^ Recent studies have revealed that exosomes—small vesicles secreted by cancer cells—play a critical role in facilitating communication between tumor cells and the immune system.^[^ ^9^, ^10^ ^]^ These exosomes carry bioactive molecules, including proteins, lipids, and RNAs, which can alter the behavior of recipient cells within the TME.

The CKLF Like MARVEL Transmembrane Domain Containing (CMTM) gene family has been identified as a key regulator of tumorigenesis, although its precise role in OC progression remains poorly understood.^[^ ^11^ ^]^ Notably, CMTM4, a member of this gene family, has been implicated in immune modulation and tumor progression.^[^ ^12^ ^]^ While the involvement of exosomal CMTM4 in immune regulation has been proposed, its specific mechanisms in OC remain unclear.

In this study, we investigated the role of exosomal CMTM4 in the OC microenvironment, with a particular focus on its effects on M2 macrophage polarization, immunosuppression, and tumor progression. Elucidating the molecular mechanisms by which CMTM4 drives M2 polarization may offer valuable insights for the development of innovative therapeutic strategies targeting the tumor immune microenvironment (TIME) in OC.

## 2 Results

### 2.1 Upregulated CMTM4 is Associated with Poor Prognosis in Patients with OC

**Figure 1:**
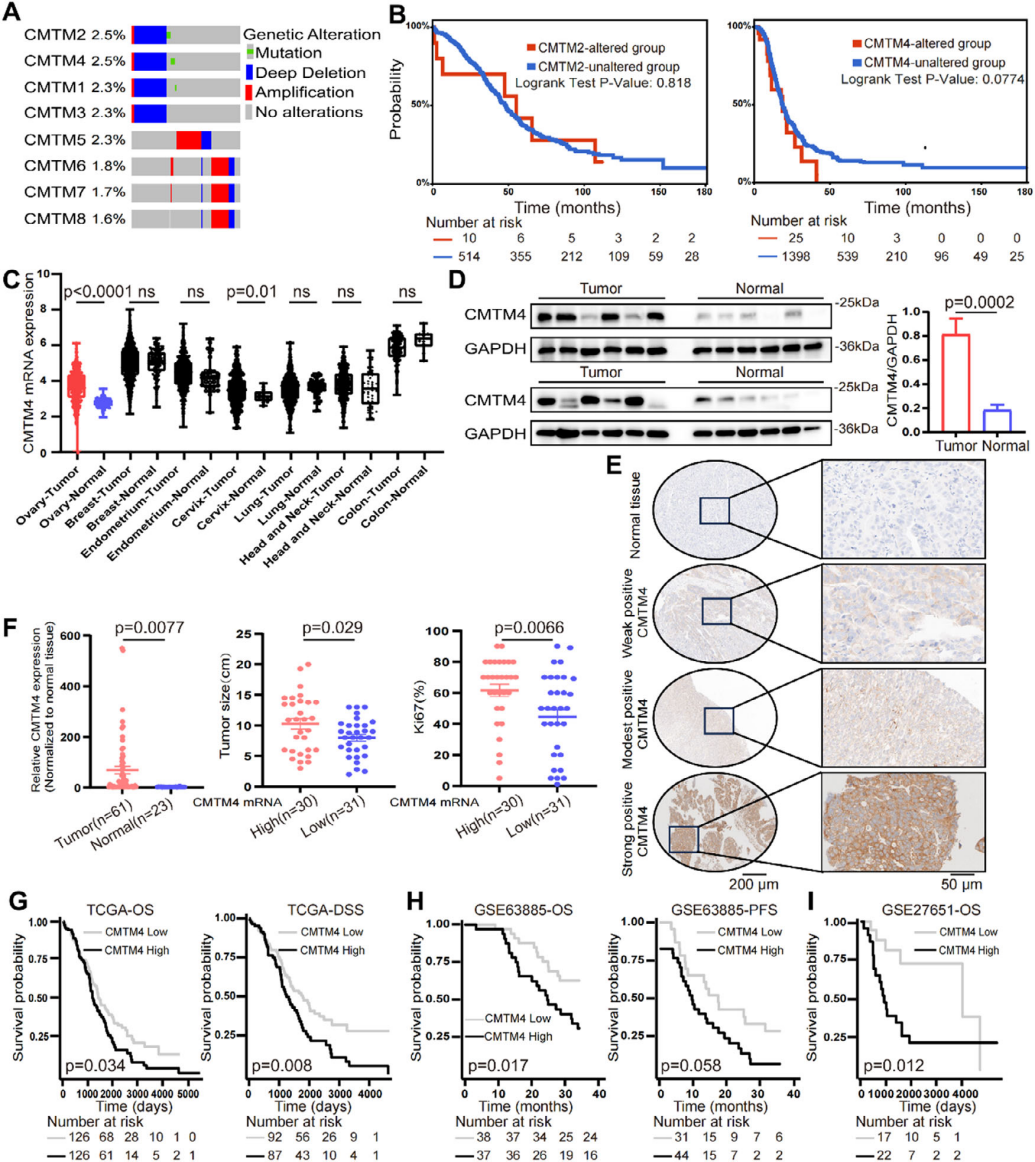
Analysis of CKLF Like MARVEL Transmembrane Domain Containing 4 (CMTM4) expression and its correlation with prognosis in ovarian cancer (OC). A‐B) Analyses were performed using the cBioPortal database (https://www.cbioportal.org/). (A) Copy number variations of CMTM family genes in OC from the TCGA database were analyzed. (B) The relationship between genetic alterations in CMTM4 and CMTM2 and survival in patients with OC from TCGA. Statistical analysis using the log‐rank test. C) Comparison of CMTM4 expression levels between cancer and normal tissues across various cancer types shown in the TCGA database. Data are presented as the mean ± standard error of the mean (SEM); unpaired two‐sided Student's t‐test (compared to normal control). ns, not significant. D) Protein levels of CMTM4 in fresh ovarian tissue (tumor versus normal) from Shanghai First Maternity and Infant Health Hospital were detected using western blotting (WB) (left) and quantified (right) using ImageJ software (n = 10). Data are presented as the mean ± SEM; unpaired two‐sided Student's t‐test (compared to normal control, p = 0.0002). E) CMTM4 expression was detected by IHC staining in different OC samples and normal tissues. Representative images are displayed. The left panels display images at low magnification (10×), while the right panels present the corresponding regions at high magnification (40×). F) mRNA expression levels of *CMTM4* in clinical samples (normalized to normal tissue) were analyzed, and the correlation between *CMTM4* expression and clinical parameters, including tumor size and Ki67 proliferation index, was assessed. Data are presented as the mean ± SEM and fold change relative to the normal group; unpaired two‐sided Student's t‐test. G) The TCGA dataset was employed to explore the relationship between CMTM4 expression and overall survival (OS; p = 0.034) and disease‐specific survival (DSS; p = 0.008) in patients with OC. Statistical analysis using the log‐rank test. H‐I) Kaplan–Meier survival curves (http://kmplot.com/analysis/) were plotted using GSE63885 (H) and GSE27651 (I) datasets to demonstrate the relationship between CMTM4 expression, OS, and progression‐free survival (PFS) in patients with OC. All statistical analyses were performed using GraphPad Prism.

We initially analyzed copy number variations (CNVs) of CMTM family genes in The Cancer Genome Atlas (TCGA) using the cBioPortal database.^[^ ^13^ ^]^ As shown in **Figure** **1**A, CMTM2 and CMTM4 exhibited the highest number of CNVs among the eight molecules analyzed. Notably, unlike CMTM2, genetic alterations in CMTM4 were associated with reduced survival duration in patients with OC (Figure 1B). Additionally, CMTM4 was expressed at significantly higher levels than CMTM2 in OC, and CMTM4 was overexpressed in cancerous tissues compared to normal tissues, whereas the opposite was true for CMTM2 (Figure S1A, Supporting Information). Therefore, we selected CMTM4 as the target gene for further investigation.

Next, we observed that, compared to other cancer types, the difference in CMTM4 expression between OC and normal samples was particularly pronounced, and its expression was indicative of poor prognosis (Figure 1C; Figure S1B, Table S1, Supporting Information). Subsequently, we used publicly available datasets beyond TCGA to examine CMTM4 expression in human OC. Analyses of the Clinical Proteomic Technology Assessment for Cancer cohort and Gene Expression Omnibus (GEO) series further revealed that CMTM4 expression is elevated in human OC tissues (Figure S1C‐D, Supporting Information).^[^ ^14^ ^]^ Moreover, the Cancer Cell Line Encyclopedia (CCLE) project confirmed that epithelial OC cells exhibit higher CMTM4 expression than normal immortalized cell lines (Figure S1E, Supporting Information).^[^ ^15^ ^]^ We assessed CMTM4 expression in human OC and normal tissues obtained from the Shanghai First Maternity and Infant Health Hospital. Our analysis also revealed that OC tissues exhibited considerably higher CMTM4 protein expression (Figure 1D). Consistent with these findings, Immunohistochemistry (IHC) staining demonstrated that the expression of CMTM4 varied among different OC samples, but was consistently higher in all OC tissues than in normal tissues (Figure 1E). In our cohort, CMTM4 mRNA levels in OC tissues were markedly elevated and analysis revealed a strong positive correlation between high CMTM4 expression and tumor size, Ki67 proliferation index, Tumor‐Node‐Metastasis stage, and histological differentiation (Figure 1F, **Table** **1**). In addition, Kaplan–Meier survival analysis across multiple cohorts showed that patients with OC with higher CMTM4 levels had shorter survival times, and subsequent univariate Cox regression analysis indicated that CMTM4 was a risk factor for OC (Figure 1G‐I; Table S2, Supporting Information). We further evaluated the prognostic value of CMTM4 using the Gene Expression Profiling Interactive Analysis (GEPIA), Human Protein Altas (HPA), OncoLnc, and TIMER2.0 databases, and found that CMTM4 was strikingly elevated in OC tissues, with increased expression associated with poorer prognoses (Figure S1F‐J, Supporting Information).^[^ ^16^ ^]^ Receiver operating characteristic curve analysis demonstrated that elevated CMTM4 is an independent prognostic indicator for patients with OC (Figure S1K, Supporting Information). These findings suggest that CMTM4 is upregulated in OC and may serve as a marker of adverse prognosis.

**Table 1:** Relationship between CMTM4 mRNA expression and clinicopathological parameters in patients with OC.

Characteristics	Low expression of CMTM4	High expression of CMTM4	*P* value
N	31	30	
Age (years) ≥55 <55	13 18	15 15	0.61
Grade G1+G2 G3	20 11	8 22	0.0046
T‐Stage			
T1+T2 T3+T4	29 2	14 16	<0.0001
N‐Stage			
N0 N1	27 4	26 4	0.99
M‐Stage			
M0 M1	22 9	14 16	0.071
CA125(U mL^−1^)			
≥500 <500	27 4	23 7	0.33

Chi‐square test.

### 2.2 CMTM4 Alters the Immune Microenvironment to Drive OC Progression via Macrophages

**Figure 2:**
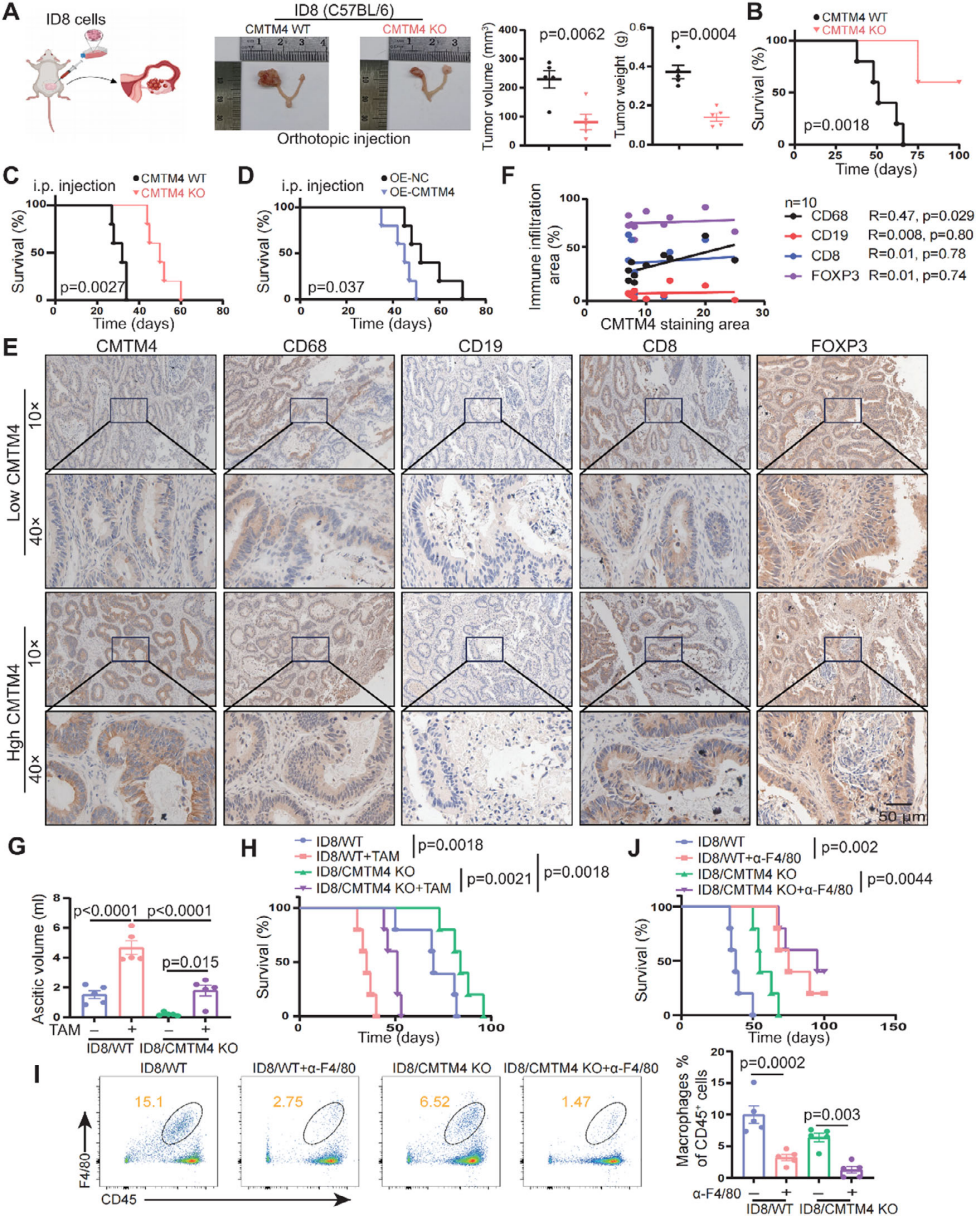
CMTM4 drives OC progression by modulating the immune microenvironment through macrophages. A) Schematic diagram of the orthotopic OC model (left). To evaluate the in vivo role of CMTM4, 1 × 10⁶ ID8 cells were orthotopically implanted into female C57BL/6 recipient mice (n = 5 mice per group). At the experimental endpoint, orthotopic syngeneic tumors were harvested, and their volume and weight were measured. Data are presented as the mean ± SEM; unpaired two‐sided Student's t‐test (compared to the CMTM4 KO group). B) Kaplan–Meier survival curves of mice (n = 5), analyzed using the log‐rank test (p = 0.0018). C‐D) To further assess the tumorigenic potential of CMTM4 in vivo, C57BL/B6 mice (n = 5) received an intraperitoneal (i.p.) injection of ID8 cells. (C) Kaplan–Meier survival curves of mice bearing i.p. tumors (ID8/WT versus ID8/CMTM4 KO; 3 × 10⁶ ID8 cells; p = 0.0027; n = 5). (D) Kaplan–Meier survival curves of mice bearing i.p. tumors (ID8/OE‐NC versus ID8/OE‐CMTM4; 2 × 10⁶ ID8 cells; p = 0.037; n = 5). E) IHC staining for CD68⁺ macrophages, CD19⁺ B cells, CD8⁺ T cells, and FOXP3⁺ Tregs in OC tissues with high and low CMTM4 expression. Representative images are shown. Upper panels display low magnification (10×), and lower panels show the corresponding regions at high magnification (40×). Scale bar: 50 µm. F) Pearson correlation and linear regression analysis of immune cell infiltration biomarkers and CMTM4 expression in human OC tissues (n = 10). G‐H) TAMs treated with ID8−CM were mixed with ID8 cells at a 1:1 ratio and administered to recipient mice via i.p. injection (n = 5 mice per group). G) Mice were euthanized at the endpoint and ascites volumes were collected and quantified (n = 5). Data are presented as the mean ± SEM; one‐way ANOVA followed by Tukey's multiple comparisons test. H) Kaplan–Meier survival curves of ID8‐bearing mice with or without exogenous TAMs (n = 5) and analyzed using the log‐rank test. (I‐J) A total of 4 × 10⁶ ID8 cells (ID8/WT versus ID8/CMTM4 KO) were injected into the peritoneal cavity (n = 5 mice per group). I) Infiltration ratio of CD45^+^F4/80^+^ macrophages in hemorrhagic ascites (n = 5). Data are presented as the mean ± SEM; one‐way ANOVA followed by Tukey's multiple comparisons test. Representative flow cytometry (FCM) results are shown. J) Kaplan–Meier survival curves of ID8‐bearing mice with or without α‐F4/80 treatment (n = 5) and analyzed using the log‐rank test. All statistical analyses were performed using GraphPad Prism and FCM data were analyzed by FlowJo.

To delve deeper into the role of CMTM4 in OC development, we analyzed the expression of CMTM4 in several OC cell lines. Accordingly, we knocked out the CMTM4 gene in A2780 and ES2 cell lines (Figure S2A‐B, Supporting Information). Given the low CMTM4 expression in HEY cell line, we also established a CMTM4‐overexpressing (CMTM4‐OE) HEY cell line using an overexpression vector (Figure S2C, Supporting Information). The efficiency of gene knockout and overexpression was verified by WB. Notably, neither knockout nor overexpression of CMTM4 had a considerable impact on tumor cell proliferation and apoptosis in vitro (Figure S2D‐I, Supporting Information). Similarly, alterations in CMTM4 expression did not affect tumor cell migration or invasion in vitro (Figure S2J‐L, Supporting Information). Sequence alignment revealed a high degree of conservation between human and mouse CMTM4 (Figure S2M, Supporting Information). Following stable knockout of CMTM4 in the ID8 cell line, we orthotopically transplanted these cells into C57BL/6 mice (**Figure** **2**A; Figure S3A, Supporting Information). In contrast to the in vitro findings, silencing CMTM4 significantly inhibited tumor growth in vivo (Figure 2A). Mice bearing ID8/WT tumors exhibited substantial hemorrhagic ascites, metastases at multiple peritoneal sites, and tumor‐induced splenomegaly (Figure S3B‐C, Supporting Information). However, these phenomena were notably attenuated in ID8/CMTM4 KO tumor‐bearing mice, which was associated with prolonged survival (Figure 2B; Figure S3D, Supporting Information). Hematoxylin‐Eosin staining showed that the presence of CMTM4 in tumor tissue increased atypical cell production, and Immunofluorescence (IF) staining confirmed a significant reduction in Ki67‐ and CD44‐positive (invasive marker) regions in ID8/CMTM4 KO tumors (Figure S3E, Supporting Information). Tumor progression was also suppressed in mice bearing ID8/CMTM4 KO tumors following intraperitoneal (i.p.) injection. These mice exhibited reduced tumor growth, diminished ascites formation, smaller spleens, fewer peritoneal implantations, and decreased Ki67 and CD44 expression in peritoneal lesions compared to ID8/CMTM4 WT tumor‐bearing mice (Figure S3F‐J, Supporting Information). Consequently, the survival of ID8/CMTM4 KO mice was notably prolonged (Figure 2C; Figure S3K, Supporting Information). As expected, i.p. injection of ID8 cells overexpressing CMTM4 promoted OC progression, accelerating the development of the malignant features and reducing mouse survival (Figure 2D; Figure S3L‐P, Supporting Information). The discrepancy between CMTM4's in vivo and in vitro effects suggests that its primary role is in modulating TIME, rather than acting directly on tumor cells.

To explore this, we analyzed the association between CMTM4 expression and CD45^+^ tumor‐infiltrating leukocytes (including CD68^+^ macrophages, CD19^+^ B cells, CD8^+^ T cells, and FOXP3^+^ regulatory T cells [Tregs]) in the TCGA‐OC cohort. This analysis revealed a strong correlation between CMTM4 levels and increased CD68^+^ macrophage infiltration (Figure S4A, Supporting Information), which was further confirmed by IHC staining (*n*  =  10) (Figure 2E‐F). IF analysis of mouse orthotopic tumors also demonstrated a significant reduction of CD68^+^ macrophages in the CMTM4‐KO group (Figure S4B, Supporting Information). Additionally, immune profiling of the peritoneal cavity revealed a reduction in F4/80 macrophages, accompanied by a decrease in CD8^+^ T cells and a marked increase in Tregs (Figure S5A‐B, Supporting Information). A similar pattern was observed in tumor‐induced ascites from mice who received an i.p. injection of ID8 cells (Figure S5C‐D, Supporting Information). Data from the TCGA‐OC cohort and the TIMER database further supported the positive correlation between macrophage infiltration and CMTM4 expression (Figure S4C‐D, Supporting Information). Importantly, stratification of TCGA samples into high‐ and low‐macrophage infiltration groups revealed that CMTM4 expression significantly affected OS only in patients with high infiltration, whereas it had no substantial impact in those with low infiltration (Figure S4E, Supporting Information). To determine whether the impaired growth of ID8/CMTM4 KO tumors was due to reduced macrophage presence, we generated TAMs by exposing RAW264.7 cells to conditioned media (CM) from ID8. Consistent with previous studies,^[^ ^17^ ^]^ these TAMs exhibited significant upregulation of both M1 and M2 macrophage phenotypes (Figure S4F, Supporting Information). We then administered equal proportions of conditioned RAW264.7 and ID8 cells into naïve recipient mice via i.p. injection. Co‐administration of TAMs markedly enhanced the growth of ID8/CMTM4 KO tumors and increased ascites accumulation to levels comparable to those in ID8/WT tumors (Figure 2G; Figure S4G‐H, Supporting Information). Correspondingly, the survival of mice bearing ID8/CMTM4 KO tumors was significantly reduced following TAM supplementation (Figure 2H). Furthermore, we depleted macrophages using an α‐F4/80 monoclonal antibody (mAb) in ID8‐bearing mice (Figure S4I, Supporting Information). Macrophage depletion effectively reduced ascites accumulation, and the number of peritoneal TAMs fell below those observed in the ID8/CMTM4 KO group (Figure 2I; Figure S4I, Supporting Information). Notably, ID8/CMTM4 KO bearing mice lacking TAMs demonstrated the lowest tumor burden and the longest survival (Figure 2J). These findings indicate that CMTM4 deficiency in tumors compromises TAM’ infiltration and reduces their immunosuppressive activity.

### 2.3 Exosomal CMTM4 Targets Macrophages to Induce Immunosuppression and Promote OC Progression

**Figure 3:**
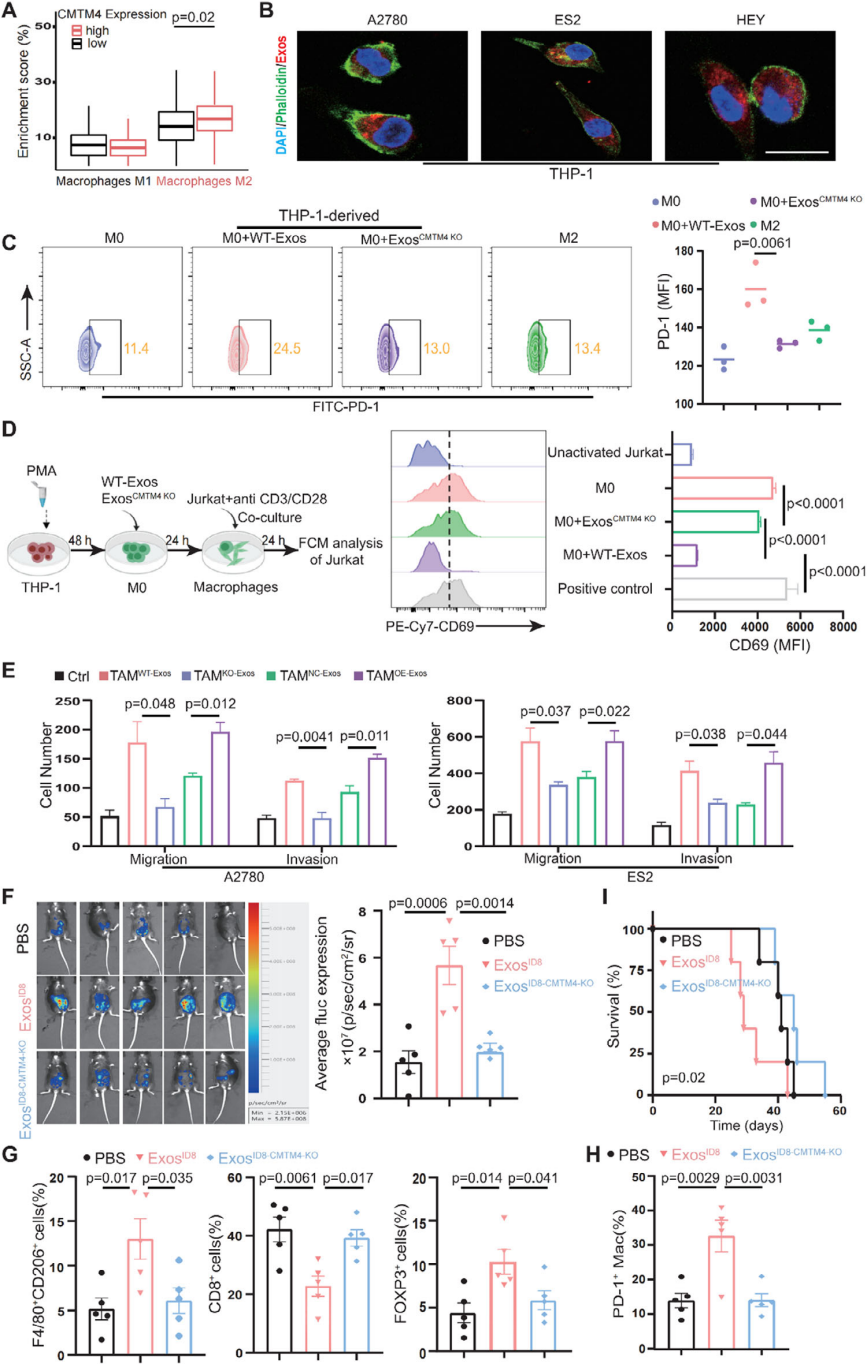
Exosomal CMTM4 acts on macrophages to induce immunosuppression and enhance OC malignancy. A) Patients in the TCGA‐OC cohort were stratified into high and low CMTM4 expression groups to evaluate the differences in the proportions of M1 and M2 macrophages. Data are presented as the mean ± SEM. Statistical analysis was performed using the Wilcoxon rank‐sum test. B) Laser scanning confocal microscopy images illustrating the uptake of OC cell‐derived exosomes (A2780, ES2, and HEY cell lines) by macrophages. Scale bar, 25 µm. C) THP‐1‐derived macrophages were cocultured with exosomes from the control or CMTM4−KO A2780 OC cell line for 24 h. FCM analysis was then performed to determine the percentage of PD‐1^+^ macrophages. IL‐4‐treated macrophages (M2) served as the positive control. Data are presented as the mean ± standard deviation (SD); one‐way ANOVA followed by Tukey's multiple comparisons test (n = 3 independent experiments per group). Mean fluorescence intensity (MFI) was analyzed using FlowJo. D) Schematic diagram illustrating the co‐culture workflow of macrophages and Jurkat T cells (activated using anti‐CD3/CD28 antibodies) (left). Jurkat T cells were collected for FCM analysis to assess CD69 expression across different groups. Jurkat T cells alone were defined as un‐activated, while those treated with anti‐CD3/CD28 without macrophage co‐culture served as positive controls. Data are presented as the mean ± SD; one‐way ANOVA followed by Tukey's multiple comparisons test (n = 3 independent experiments per group). MFI was analyzed using FlowJo. E) OC cell lines were co‐cultured with macrophages treated with WT‐Exos versus KO‐Exos, or OE‐Exos versus NC‐Exos for 24 h, followed by a Transwell assay to assess migratory and invasive abilities of A2780 (left) and ES2 (right) cells. Data are presented as the mean ± SD; unpaired two‐sided Student's t‐test (n = 3 independent experiments per group). Cell counts were quantified using ImageJ software. F) A mixture of 3 × 10^6^ ID8 cells and 3 × 10^5^ (RAW264.7) macrophages (10:1) with specified treatments was administered to mice via i.p. injection. In vivo bioluminescence imaging was conducted on day 30 (n = 5 per group). Data are presented as the mean ± SEM; one‐way ANOVA followed by Tukey's multiple comparisons test. G) FCM analysis of immune cell subsets in mouse ascites, including CD45^+^F4/80^+^CD206^+^ macrophages, CD45^+^CD3^+^CD8^+^ T cells, and CD45^+^CD3^+^CD4^+^FOXP3^+^ Tregs across three groups (n = 5). Data are presented as the mean ± SEM; one‐way ANOVA followed by Tukey's multiple comparisons test. H) FCM analysis of CD45^+^F4/80^+^CD206^+^PD‐1^+^ macrophages in mouse ascites across three groups (n = 5). Data are presented as the mean ± SEM; one‐way ANOVA followed by Tukey's multiple comparisons test. I) Kaplan–Meier survival analysis of mice co‐injected with ID8 cells and macrophages under different treatment conditions (p = 0.02; n = 5 per group), Statistical analysis was performed using the log‐rank test. All statistical analyses were conducted using GraphPad Prism, and FCM results were analyzed with FlowJo.

We next analyzed the impact of the M2‐to‐M1 macrophage ratio on OC prognosis and found that higher M2 and lower M1 infiltration were correlated with poor outcomes (Figure S6A, Supporting Information). Within the TME, macrophages are prone to differentiate into the M2 phenotype.^[^ ^18^ ^]^ The prognostic effects of M2 macrophages and CMTM4 expression were similar, suggesting that CMTM4 may drive tumor progression by regulating M2 polarization. Analysis of the TCGA‐OC dataset revealed a positive correlation between M2 infiltration and CMTM4 expression (**Figure** **3**A). FCM analysis of ascites in animal models showed that CMTM4 downregulation decreased CD206^+^ (M2) macrophage levels, while CMTM4 overexpression ascended this population (Figure S6B‐C, Supporting Information). Knockdown of CMTM4 in IL‐4‐treated M2 macrophages overwhelmingly inhibited their development, and in vitro co‐culture confirmed that tumor cell‐derived CMTM4 boosted M2 polarization, indicating that CMTM4 promotes macrophage‐mediated malignancy (Figure S6D‐G, Supporting Information). Additional analysis using public datasets supported the positive correlation between CMTM4 expression and M2 macrophage infiltration in OC (Figure S6H‐J, Supporting Information).

Recent studies have shown that exosomes are essential for regulating interactions between tumor cells and TAMs.^[^ ^19^ ^]^ We hypothesized that CMTM4 is packaged into exosomes and transported to monocytes, promoting their differentiation into M2 macrophages. Exosomes isolated from OC cells were confirmed by transmission electron microscopy and particle size analysis, showing a bilayer morphology and diameters ranging from 50–200 nm (Figure S7A‐B, Supporting Information). WB analysis confirmed the presence of CMTM4 along with exosome markers (CD9‐positive, calnexin‐negative) (Figure S7C, Supporting Information). Confocal microscopy further showed that Dil‐labeled exosomes (red) were distributed within macrophages (Figure 3B; Figure S7D, Supporting Information). Furthermore, CMTM4 levels in M0 macrophages gradually increased upon incubation with exosomes (Figure S7E‐F, Supporting Information), suggesting that CMTM4 is encapsulated in exosomes and delivered to macrophages.

To validate this, exosomes were collected from CMTM4‐altered cells and used to educate macrophages. CMTM4 levels in exosomes were confirmed by WB (Figure S7G‐J, Supporting Information). Macrophages treated with CMTM4‐KO exosomes (Exos^CMTM4 KO^) exhibited fewer M2 macrophages than those treated with control exosomes (WT‐Exos) (Figure S8A‐B, Supporting Information). Conversely, exosomes derived from CMTM4‐overexpressing OC cells critically increased the proportion of M2 macrophages (Figure S8C, Supporting Information). These findings suggest that exosomal CMTM4 plays a pivotal role in driving macrophage polarization toward the M2 phenotype. M2 macrophages exert immunosuppressive effects within the TME. Therefore, we evaluated whether CMTM4‐mediated M2 polarization in OC contributes to immunosuppression. Our results demonstrated that exosomal CMTM4 upregulated the immune checkpoint molecules (ICMs) PD‐1 and TIM3 on macrophages, while the expression of PD‐L1, CTLA4, and LAG3 remained unchanged (Figure 3C; Figure S8D‐E, Supporting Information). Given that PD‐1 expression on macrophages can suppress T cell activity, we further investigated whether exosomal CMTM4‐induced PD‐1 expression on macrophages modulates T cell activity and immune responses. We co‐cultured macrophages under various treatments with Jurkat T cells. CD69, an early T cell activation marker, increased in the Jurkat T cells stimulated with anti‐CD3/CD28 antibodies but decreased when co‐cultured with macrophages pretreated with WT‐Exos. In contrast, macrophages stimulated with Exos^CMTM4 KO^ restored CD69 expression levels (Figure 3D). Moreover, IFN‐γ secretion by T cells was diminished in co‐cocultures with WT‐Exos‐treated macrophages compared to those treated with Exos^CMTM4 KO^ (Figure S8F, Supporting Information). These findings indicate that macrophages exposed to exosomal CMTM4 acquire enhanced immunosuppressive capacity.

To further investigate the tumor‐promoting effects of exosomal CMTM4, we constructed a coculture system to examine how tumor exosomal CMTM4 internalized by TAMs influences tumor behavior (Figure S9A, Supporting Information). The migratory and invasive capabilities of tumor cells were notably enhanced following co‐culture with TAMs pretreated with exosomal CMTM4 (Figure 3E; Figure S9B‐C, Supporting Information). In vivo, i.p. injection of exosomes from ID8 cells promoted peritoneal metastasis in the WT‐Exos group compared to that in the PBS‐treated and Exos^CMTM4 KO^ groups (Figure 3F; Figure S9D, Supporting Information). Similarly, exosomes from CMTM4‐overexpressing cells (Exos^OE‐CMTM4^) boosted OC metastasis more effectively than the control exosomes (Exos^NC^) (Figure S9E, Supporting Information). Given the impact of exosomal CMTM4 on tumor progression, we next examined its effects on the immune microenvironment. Notably, CD8^+^ T cells in ascitic fluid increased, whereas M2 macrophages, Tregs, and PD‐1 expression on macrophages decreased in the Exos^CMTM4 KO^ and Exos^NC^ group (Figure 3G‐H; Figure S10A‐B, Supporting Information). Mouse survival also improved in the absence of exosomal CMTM4 supplementation (Figure 3I; Figure S9F, Supporting Information). Together, these results suggest that exosomal CMTM4 is phagocytosed by macrophages, promoting their polarization toward a more malignant M2 phenotype, and fostering an immunosuppressive microenvironment and tumorigenesis.

### 2.4 Exosome‐Mediated Regulation of M2 Polarization by CMTM4 via NF‐κB Requires ICAM1 in OC Development

**Figure 4:**
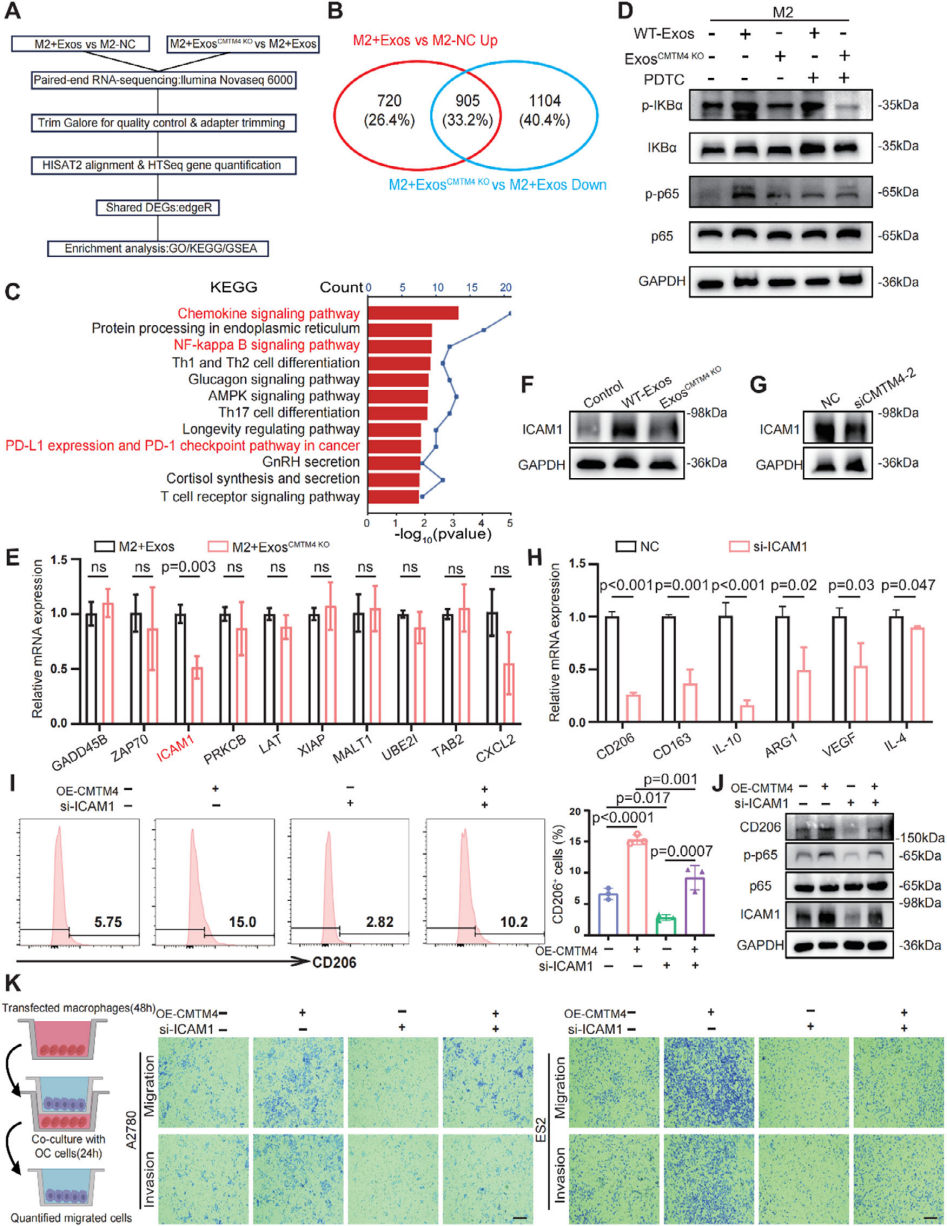
CMTM4 promotes M2 macrophage polarization and enhances OC metastasis by upregulating ICAM1. A) Schematic diagram of the RNA sequencing workflow for macrophages. Common DEGs between the two groups (M2+Exos versus M2‐NC or M2+Exos^CMTM4 KO^ versus M2+Exos) were identified and subjected to enrichment analysis. B) Venn diagram showing the shared DEGs in M2 macrophages regulated by Exos^CMTM4^ or Exos^CMTM4 KO^. The number of shared and unique genes is indicated. C) Bar chart illustrating the enriched signaling pathways of shared DEGs. Bar length represents the p‐value, and the corresponding y‐axis values indicate the number of genes. D) M2 macrophages were treated for 48 h with exosomes derived from OC cells with or without CMTM4 KO, with or without pretreatment using 200 µM PDTC. WB was performed to evaluate the phosphorylation of IκBα and p65. Representative WB images are shown (n = 3 independent experiments per group). E) M2 macrophages were treated for 24 h with exosomes from OC cells with or without CMTM4 KO. Quantitative Polymerase Chain Reaction (qPCR) was used to evaluate the mRNA expression levels of genes (*GADD45B, ZAP70, PRKCB, ICAM1, LAT, XIAP, MALT1, UBE2I, TAB2, CXCL2*) between the two groups. Data are presented as the mean ± SD; unpaired two‐sided Student's t‐test. Results are shown as fold change relative to the M2+Exos^CMTM4 KO^ group, which is set to 1 after normalization to GAPDH (n = 3 independent experiments per group); ns, not significant. F) WB analysis of ICAM1 in macrophages treated with exosomes with or without CMTM4 for 48 h. Representative WB images were shown (n = 3 independent experiments per group). G) WB analysis of ICAM1 in macrophage treated with CMTM4‐siRNA or control. Representative WB images were shown (n = 3 independent experiments per group). H) qPCR analysis of *CD206, CD163, IL‐10, ARG1, VEGF, and IL‐4* mRNA levels in M2 macrophages treated with IL‐4 and transfected with siICAM1 or control. Data are presented as the mean ± SD; unpaired two‐sided Student's t‐test. Results are shown as fold change relative to the NC group, which is set to 1 after normalization to GAPDH (n = 3 independent experiments per group). I) M2 macrophages were transfected with the indicated plasmids or control. FCM was used to quantify the percentage of CD206‐positive macrophages. Data are presented as the mean ± SD; one‐way ANOVA followed by Tukey's multiple comparisons test (n = 3 independent experiments per group). J) WB analysis of ICAM1, p65, p‐p65, and CD206 expression in M2 macrophages under the indicated treatment. Representative WB images are shown (n = 3 independent experiments per group). K) M2 macrophages were transfected with the indicated plasmids or control for 48 h, then seeded into the lower chamber of a co‐culture system (left). OC cells were placed in the upper chamber (8 µm pore size). After 24 h, the migratory and invasive abilities of OC cells (A2780, ES2) were quantified using ImageJ software. All panels are the same magnification. Scale bar, 100 µm. Representative images are shown (n = 3 independent experiments per group). All statistical analyses were performed using GraphPad Prism and FCM results were analyzed with FlowJo.

To elucidate how CMTM4 influences M2 macrophages, we subjected M2 macrophages treated with OC‐derived exosomes to RNA sequencing (RNA‐seq) (**Figure** **4**A). The analysis revealed 1,625 upregulated genes in the M2+Exos group compared to the NC group and 2,009 downregulated genes in the M2+Exos^CMTM4 KO^ group compared to the M2+Exos group. A Venn diagram in Figure 4B highlights 905 overlapping genes between the two comparisons, and which were subsequently analyzed for enrichment analysis. Gene Ontology analysis of the transcriptomic data indicated that CMTM4 modulates various macrophage‐associated functions, including response to chemokine, cytokine production, and chemokine activity (Figure S11A‐B, Supporting Information). Kyoto Encyclopedia of Genes and Genomes (KEGG) pathway analysis identified significant alterations in several pathways related to M2 macrophage polarization, such as the chemokine signaling, NF‐kappa B pathway, and PD‐L1/PD‐1 checkpoint pathway (Figure 4C). Gene Set Enrichment Analysis further revealed the enrichment of multiple M2‐macrophage‐related pathways in macrophages educated with exosomal CMTM4 (Figure S11C, Supporting Information), supporting a role for CMTM4 in promoting M2 polarization.

To investigate the regulatory relationship between exosomal CMTM4, the NF‐κB pathway, and macrophages, we assessed for p65 (p‐p65) and IκBα phosphorylation in exosome‐treated macrophages—key markers of NF‐κB activation. WB revealed that exosomal CMTM4 induced phosphorylation of both p65 and IκBα. To assess the role of the CMTM4–p65–NF‐κB axis, M2 macrophages were treated with the NF‐κB pathway inhibitor PDTC, which suppressed the phosphorylation of p65 and IκBα induced by exosomal CMTM4 (Figure 4D). Additionally, the WT‐Exos group exhibited significant p65 nuclear translocation, which was inhibited by PDTC treatment (Figure S11D, Supporting Information). In macrophages overexpressing CMTM4, PDTC not only reversed the increase in p‐p65 levels (Figure S11E‐F, Supporting Information) but also suppressed CMTM4‐driven M2 polarization (Figure S11G, Supporting Information), providing further evidence that CMTM4 regulates macrophage polarization through the NF‐κB pathway. Finally, ICMs such as PD‐1 and PD‐L1 were upregulated in macrophages treated Exos^CMTM4^, an effect that was partially reversed by PDTC treatment (Figure S8D, Supporting Information).

Subsequently, we identified potential target genes of exosomal CMTM4 in macrophages. RNA‐seq data analysis focused on genes involved in the NF‐κB signaling pathway revealed ten upregulated genes in macrophages treated with WT‐Exos (Figure S12A, Supporting Information). Among these differentially expressed genes (DEGs), *ICAM1*—a key regulatory factor in the NF‐κB pathway—was the most significantly upregulated gene in macrophages treated with CMTM4‐containing exosomes (Figure 4E). Furthermore, Spearman's correlation analysis in the TCGA‐OC cohort revealed a significant positive correlation between CMTM4 and ICAM1 expression (Figure S12B, Supporting Information). Similar to CMTM4, ICAM1 was highly expressed in OC tissues and associated with poor prognosis (Figure S12C‐D, Supporting Information), supporting the strong association between CMTM4 and ICAM1 in OC, as well as the regulatory impact of CMTM4 on ICAM1 expression in macrophages. In addition, online database analysis revealed a positive correlation between ICAM1 expression and M2 infiltration (Figure S12E, Supporting Information). Data from the GEPIA database also indicated that ICAM1 expression was higher in M2 macrophages than in M1 or M0 subsets (Figure S12F, Supporting Information), suggesting a potential role for ICAM1 in promoting M2 polarization in OC. Based on these findings, we hypothesize that CMTM4 regulates the NF‐κB pathway by enhancing ICAM1 expression in macrophages.

To test this, we performed WB and found that ICAM1 expression was elevated in macrophages treated with CMTM4‐enriched exosomes compared to those treated with exosomes lacking or containing baseline levels of CMTM4 (Figure 4F; Figure S12G, Supporting Information). Consistent with this, ICAM1 expression was downregulated in macrophages transfected with si‐CMTM4 (Figure 4G). These findings suggest that exosomal CMTM4 enhances ICAM1 expression in macrophages. To examine whether exosomal CMTM4 regulates M2 polarization via ICAM1, we silenced ICAM1 in IL‐4‐treated M2 macrophages, which substantially reduced M2 marker expression (Figure 4H; Figure S12H, Supporting Information). Moreover, ICAM1 knock down in CMTM4‐overexpressing cells impaired CMTM4‐induced M2 polarization, as evidenced by a decrease in CD206‐positive macrophages and reduced CD206 expression. Furthermore, partial depletion of ICAM1 reversed the CMTM4‐induced upregulation of p‐p65 levels (Figure 4I‐J). These findings indicate that CMTM4 requires ICAM1 to activate the NF‐κB pathway and promote macrophage M2 polarization. Finally, we investigated the role of ICAM1 in CMTM4‐mediated M2 macrophage‐driven OC progression. Macrophages were polarized with IL‐4, overexpressed with CMTM4, and then subjected to ICAM1 knockdown before co‐culture with OC cells. Transwell assays showed that CMTM4 overexpression in macrophages significantly enhanced OC cell migration and invasion, whereas ICAM1 knockdown evidently attenuated this effect (Figure 4K; Figure S12I, Supporting Information). Collectively, these findings suggest that exosomal CMTM4 functionally regulates macrophages through the NF‐κB pathway in an ICAM1‐dependent manner, which is critical for M2 macrophage‐associated OC progression.

### 2.5 Exosomal CMTM4‐Mediated Augmentation of Cytokine/Chemokine Production by Macrophages

**Figure 5:**
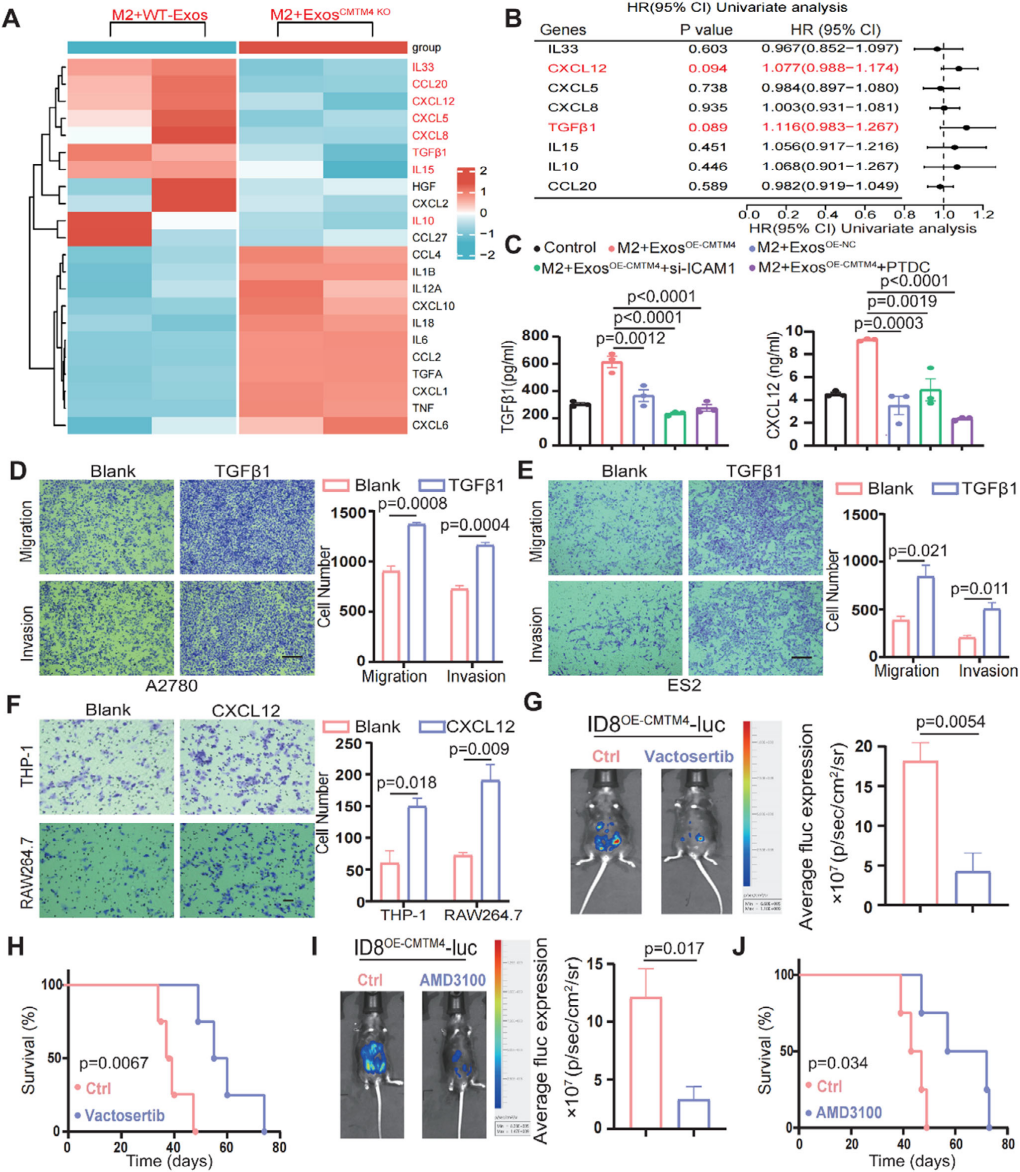
Exosomal CMTM4 enhances cytokine and chemokine production in macrophages. A) Heat‐maps showing differential cytokine and chemokine expression in macrophages treated with or without exosomal CMTM4. B) Forest plot from univariate Cox analysis of eight cytokines and chemokines (IL33, CXCL12, CXCL5, CXCL8, TGFβ1, IL15, IL10, CCL20) in the TCGA‐OC cohort. C) Concentrations of TGF‐β1 and CXCL12 in the supernatant of M2 macrophages treated with Exos^OE‐CMTM4^, Exos^OE‐NC^, Exos^OE‐CMTM4^+si‐ICAM1, or Exos^OE‐CMTM4^+PDTC, measured by ELISA. Data are presented as the mean ± SD (n = 3 independent experiments per group); one‐way ANOVA followed by Tukey's multiple comparisons test. D‐E) Transwell assays assessing the migration and invasion abilities of A2780 (D) and ES2 (E) cells after 24 h of TGF‐β1 treatment. All panels are shown at the same magnification. Scale bar, 100 µm. Data are presented as the mean ± SD (n = 3 independent experiments per group); unpaired two‐sided Student's t‐test. F) Chemotactic ability of THP‐1 (M0) and RAW264.7 cells after 24 h of CXCL12 treatment measured using the Transwell assay. All panels are shown at the same magnification. Scale bar, 100 µm. Data are presented as the mean ± SD (n = 3 independent experiments per group); unpaired two‐sided Student's t‐test. (G, I) A total of 3 × 10^6^ ID8‐luc (OE‐CMTM4) cells were injected into the peritoneal cavity of mice, followed by injection of CMTM4‐enriched exosomes (derived from ID8^OE‐CMTM4^ cells) every 10 days. In vivo bioluminescence imaging was conducted on day 30 (n = 4). G) Representative bioluminescence images and quantitative fluorescence analysis from the control (saline) and vactosertib‐treated groups. Data are presented as the mean ± SD; unpaired two‐sided Student's t‐test. H) Kaplan–Meier survival curves of mice in the control and vactosertib‐treated groups. Statistical analysis was performed using the log‐rank test (p = 0.0067). I) Representative in vivo bioluminescence images and quantitative fluorescence analysis of the control (saline) and AMD3100‐treated groups. Data are presented as the mean ± SD; unpaired two‐sided Student's t‐test. J) Kaplan–Meier survival curves of mice in the control and AMD3100‐treated groups. Statistical analysis was performed using the log‐rank test (p = 0.034). All statistical analyses were performed using GraphPad Prism.

Macrophages are widely recognized for supporting tumor cells within the TME through the secretion of various factors.^[^ ^20^ ^]^ To further investigate this, we extracted the aforementioned RNA‐seq data and analyzed the differentially expressed factors between the two groups(M2+WT‐Exos versus M2+Exos^CMTM4 KO^). We found that inflammatory cytokines and chemokines including IL33, TGFβ1, IL15, IL10, CCL20, CXCL12, CXCL5, CXCL8 associated with the NF‐κB pathway activation were significantly up‐regulated in WT‐Exos‐treated macrophages (**Figure** **5**A). Univariate Cox regression and survival analysis identified TGFβ1 and CXCL12 as the most impressive risk factors for overall survival among the differentially expressed factors (Figure 5B; Figure S13A‐B, Supporting Information). Data from TCGA further revealed that TGFβ1 and CXCL12 exhibited the highest expression levels and were predominantly expressed in M2 macrophages compared to other immune cell types, suggesting that their expression in macrophages may be regulated by tumor‐derived exosomal CMTM4 (Figure S13C‐D, Supporting Information). qPCR results further validated a significant increase in TGFβ1 and CXCL12 expression in macrophages treated with WT‐Exos compared to those treated with Exos^CMTM4 KO^ (Figure S13E, Supporting Information). Similarly, macrophages stimulated with CMTM4‐enriched exosomes exhibited higher levels of TGFβ1 and CXCL12 compared to the control group. (Figure 5C; Figure S13F, Supporting Information).

In research from other fields, ICAM1 has been shown to facilitate the secretion of TGFβ1 and CXCL12 via interaction with macrophages, therefore we determined whether CMTM4 in tumor‐derived exosomes enhances macrophage secretion of these cytokines through ICAM1 upregulation or NF‐κB activation.^[^ ^21^, ^22^ ^]^ As anticipated, both si‐ICAM1 and PDTC treatment effectively reduced the elevated secretion of these factors (Figure 5C; Figure S13F, Supporting Information). Next, we assessed whether TGFβ1 and CXCL12 contribute to tumorigenic processes in OC. We first confirmed that the addition of TGFβ1 significantly amplified the migration and invasion of OC cells, while CXCL12 facilitated macrophages recruitment (Figure 5D‐F). Finally, we examined whether elevated TGFβ1 and CXCL12 levels mediate the tumor‐promoting effects of exosomal CMTM4 in OC. To address this, we established an abdominal tumor mouse model using ID8‐luc cells overexpressing CMTM4. Specifically, CMTM4‐enriched exosomes (derived from ID8^OE‐CMTM4^ cells) were administered via i.p. injection into mice. In the treatment group, vactosertib (TGFβ1 inhibitor; 10 mg kg^−1^) or AMD3100 (CXCL12 receptor inhibitor; 5 mg kg^−1^,) was administered, while the control group received an equivalent volume of saline. Bioluminescence imaging revealed that both vactosertib and AMD3100, when administered individually, effectively suppressed tumor growth and augmented survival in mice (Figure 5G‐J). FCM analysis revealed that vactosertib significantly increased the number and proportion of CD8^+^ T cells within the TME, while concurrently reducing the proportions of M2 macrophages and Tregs (Figure S13G‐I, Supporting Information). Furthermore, treatment with AMD3100 notably ameliorated macrophage infiltration (Figure S13J, Supporting Information). Taken together, these findings indicate that paracrine factors TGFβ1 and CXCL12 contribute to exosomal CMTM4‐mediated M2 macrophage polarization and subsequent OC progression.

### 2.6 Targeting Tumor‐Derived CMTM4 for OC Treatment

**Figure 6:**
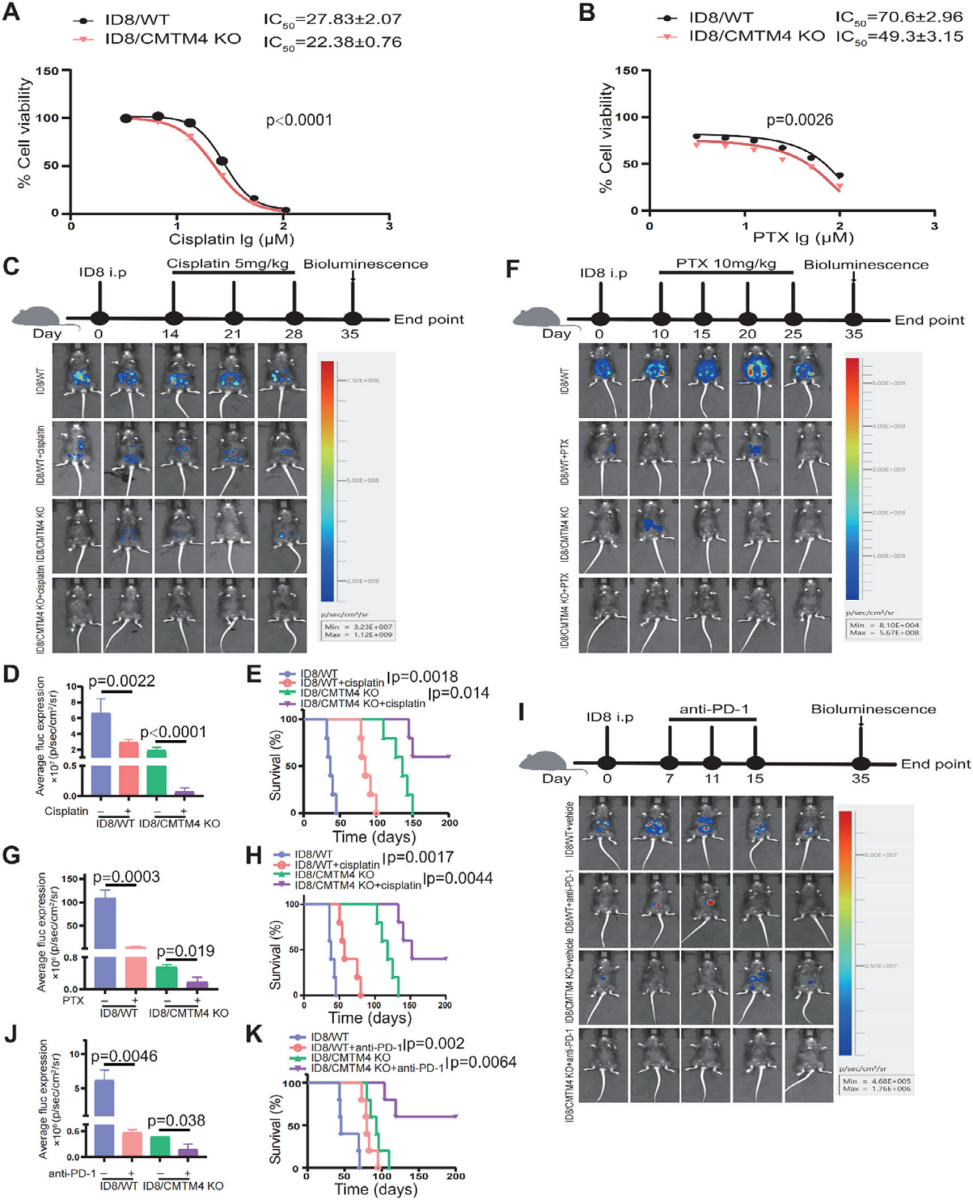
Targeting tumor‐derived CMTM4 as a therapeutic strategy for OC. A) Dose‐response curves of ID8 cells following 48‐h cisplatin treatment. B) Dose‐response curves of ID8 cells after paclitaxel (PTX) treatment. (A‐B) Cell viability in the absence of drug treatment was defined as 100% (n = 3 independent experiments per group). IC₅₀ Comparisons were performed using an unpaired two‐tailed Student's t‐test. C‐E) Presence of CMTM4 abrogated the therapeutic effect of cisplatin. ID8‐bearing mice were treated with cisplatin starting at 2 weeks after tumor implantation, administered once per week for 3 weeks (*n*  =  5 mice per group). Tumor progression and body weight (E) were monitored. In vivo bioluminescence imaging was performed on day 35 (C‐D). Data are presented as the mean ± SEM. Statistical analysis was performed using one‐way ANOVA followed by Tukey's multiple comparisons test. Mouse survival was analyzed using log‐rank test. (F‐H) CMTM4 expression modulates the response to PTX treatment. Following tumor implantation, ID8 tumor‐bearing mice received PTX every 5 days for a total of 4 times (n  =  5 mice per group). Biofluorescence imaging was conducted on day 35 F), followed by quantitative analysis G). Data are presented as the mean ± SEM; unpaired two‐tailed Student's t‐test. Mouse survival was monitored and recorded H), and analyzed using the log‐rank test. I‐K) Anti‐PD‐1 treatment inhibited tumor development in ID8‐tumor‐bearing mice and notably improved the prognosis of mice bearing ID8/CMTM4 KO tumors (K). ID8‐bearing mice treated with anti‐PD‐1 starting at 1 week after tumor implantation, administered every 4 days for a total of 3 doses (n  =  5 mice per group). In vivo fluorescence and quantification were conducted on day 35 (I‐J). Data are presented as the mean ± SEM. Statistical analysis was performed using one‐way ANOVA followed by Tukey's multiple comparisons test. Mouse survival was analyzed using the log‐rank test. All statistical analyses were performed using GraphPad Prism.

To validate the role of CMTM4 as a therapeutic target in OC, we examined the impact of CMTM4 knockout on the sensitivity of ID8 cells to two commonly used chemotherapeutic agents, cisplatin and paclitaxel (PTX), by measuring their IC_50_ values. In vitro, silencing CMTM4 sensitized OC cells to cisplatin, enabling effective induction of apoptosis at low drug concentrations (**Figure** **6**A). In vivo, tumors derived from ID8 cells lacking CMTM4 expression exhibited significantly slower growth upon cisplatin treatment, and mice experienced markedly improved survival (Figure 6C‐E). Similar results were observed with PTX treatment (Figure 6F‐H). The effects of CMTM4 knockout on tumor cell sensitivity were comparable, with ID8/CMTM4 KO cells showing increased susceptibility to PTX‐induced cell death, further supporting the role of CMTM4 in modulating drug resistance in OC (Figure 6B). Although, immunotherapy has transformed cancer treatment, it shows limited efficacy in OC, partly due to the immunosuppressive ascitic microenvironment enriched with M2‐polarized TAMs and Tregs.^[^ ^23^ ^]^ Our findings indicate that CMTM4 fosters an immunosuppressive TME by modulating TAMs, which may compromise the effectiveness of immunotherapy. Given that macrophages play a crucial role in phagocytosis—a key defense mechanism—tumor cells have been shown to evade macrophage‐mediated immune surveillance by exploiting ICMs such as PD‐L1 and CD47.^[^ ^24^ ^]^

To test this hypothesis, we evaluated the effect of CMTM4 on the expression of these ICMs. FCM analysis revealed a significant downregulation of PD‐L1 expression in CMTM4‐KO OC cells, with a similar trend observed upon CMTM4 overexpression (Figure S14A‐C, Supporting Information). In contrast, the expression of CD47, another macrophage phagocytosis checkpoint molecule, was largely unaffected (Figure S14D‐F, Supporting Information). Correlation analysis from GEPIA further corroborated these findings (Figure S14G, Supporting Information). To investigate whether CMTM4 affects macrophage phagocytosis, we conducted in vitro macrophage phagocytosis assays. Macrophages engulfed significantly more CMTM4‐silenced cells than control cells (Figure S14H‐I, Supporting Information). Next, we investigated the role of CMTM4 in modulating the efficacy of immunotherapy. Mice bearing ID8 tumors were treated with an anti‐PD‐1 mAb. Although PD‐1 blockade alone showed therapeutic effects, the combined depletion of CMTM4 and PD‐1 blockade produced a synergistic effect, resulting in minimal tumor dissemination and prolonged survival (Figure 6I‐K). In the combined treatment group, the peritoneal cavity exhibited a substantial decrease in the proportions of M2 macrophages and Tregs, along with significant increase in CD8^+^ T cells. Furthermore, PD‐1 expression was conspicuously suppressed in macrophages (Figure S14J, Supporting Information). Taken together, these findings demonstrate that targeting tumor‐derived CMTM4 can alleviate chemoresistance and enhance the efficacy of immunotherapy, thereby strongly enhancing current OC treatments.

### 2.7 Identification of Eltrombopag (ELT) as an Inhibitor of CMTM4 and OC Progression

**Figure 7:**
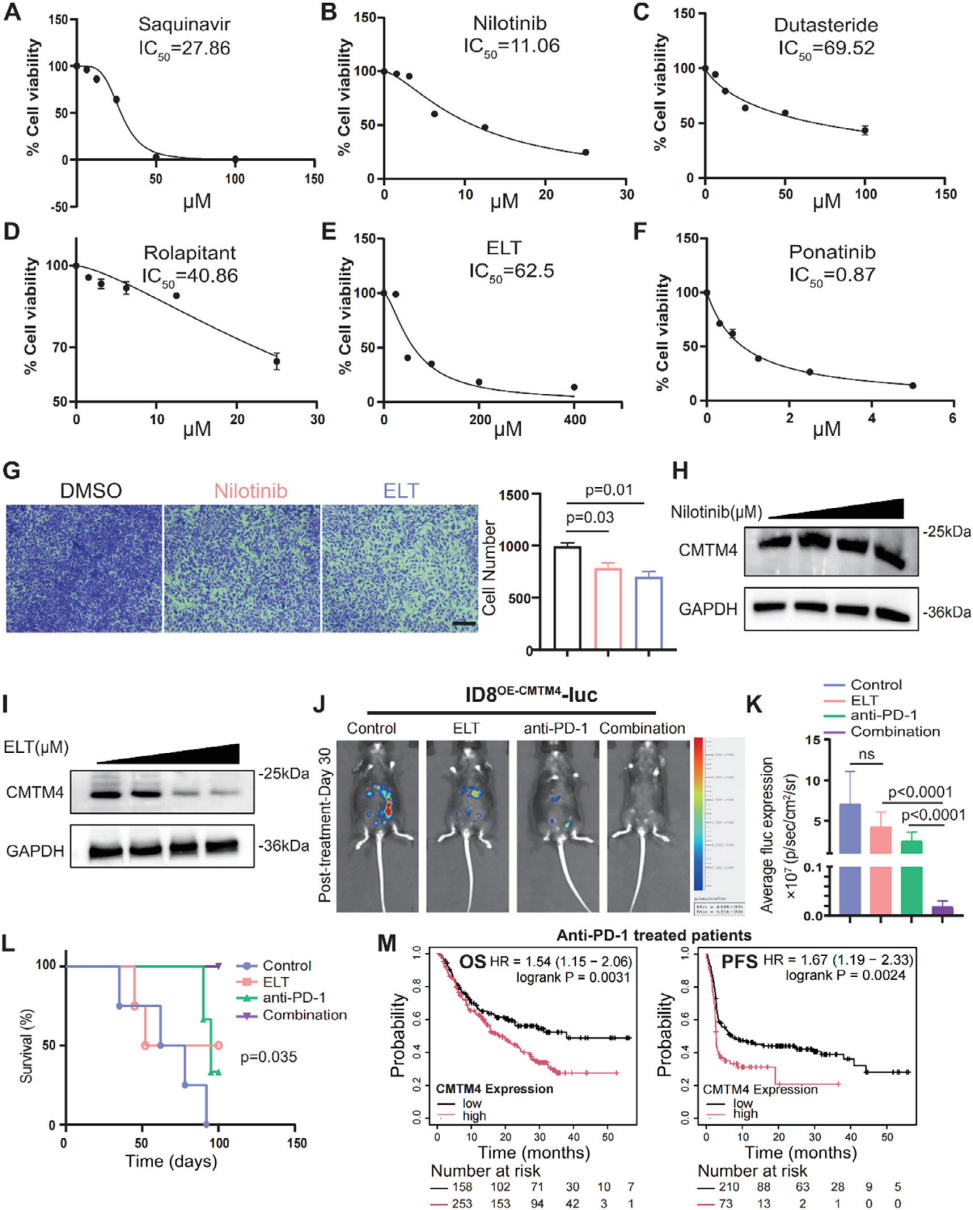
Eltrombopag (ELT) inhibits CMTM4 and synergizes with PD‐1 blockade immunotherapy. A‐F) The IC_50_ value of the candidate inhibitors was calculated by treating ID8 cells with various concentrations for 48 h. Cell viability was assessed using the CCK‐8 assay, with untreated cells set as 100% (n = 3 independent experiments per group). G) Transwell migration assays of ID8 cells treated with nilotinib and ELT for 48 h. All panels are the same magnification. Scale bar, 100 µm. Representative images are shown (n = 3 independent experiments per group). Data are presented as the mean ± SD; one‐way ANOVA followed by Tukey's multiple comparisons test. H‐I) WB analysis of CMTM4 protein expression in ID8 cells treated with increasing concentrations of nilotinib and ELT for 48 h. Representative WB images are shown (n = 3 independent experiments per group). J‐K) Mice received an i.p. injection of ID8^OE‐CMTM4^‐luc cells (3 × 10^6^) and were then treated with ELT, anti‐PD‐1 or a combination of both at day 10 and 20, followed by in vivo bioluminescence imaging to monitor tumor metastasis and quantify tumor burden (K, n = 4). In vivo bioluminescence imaging was performed on day 30 to evaluate the therapeutic effects (J). Data are presented as the mean ± SD; unpaired two‐tailed Student's t‐test. ns, not significant. L) Kaplan–Meier survival analysis of mice treated with ELT and anti‐PD‐1 (n = 4 per group; p =  0.035). Statistical analysis was performed using the log‐rank test. M) Comparison of OS (left) and PFS (right) in the combined cancer types (bladder, glioblastoma, melanoma, and other tumors) patients who had high CMTM4 versus low CMTM4 intra‐tumoral expression receiving anti‐PD‐1. Survival analysis was analyzed using log‐rank test. All statistical analyses were performed using GraphPad Prism.

Given the crucial role of CMTM4 in OC development, we investigated potential molecular inhibitors of CMTM4. We first applied the AlphaFold software to predict CMTM4 protein structure (Figure S15A, Supporting Information). Next, we conducted a virtual screening of potential CMTM4‐binding molecules using Autodock and Zinc, a free database of commercially available compounds. Ten compounds with low docking scores were selected for further evaluation (Table S3, Supporting Information). To explore the anti‐tumor potential of these compounds, ID8 cells were individually treated with each compound, and cell viability were measured. Among the candidates, saquinavir, nilotinib, dutasteride, rolapitant, ELT, and ponatinib effectively reduced ID8 viability (**Figure** **7**A‐F; Figure S15B‐E, Supporting Information). Transwell assays revealed that nilotinib and ELT markedly suppressed the migratory capacity of ID8 cells (Figure 7G; Figure S15F, Supporting Information). Additionally, WB analysis demonstrated that ELT inhibited CMTM4 expression in a dose‐dependent manner, whereas nilotinib had no appreciable effect (Figure 7H‐I). As predicted, the remaining compounds did not significantly alter CMTM4 expression (Figure S15G‐J, Supporting Information). Collectively, these results identify ELT as a potent small‐molecule inhibitor of CMTM4, with notable anti‐tumor activity (Figure S15K, Supporting Information). Furthermore, AutoDock‐based docking analysis revealed that ELT interacts with key amino acid residues in CMTM4, including hydrophobic residues such as Ala117 and Leu104 and, polar residues such as Thr38, Glu37, Ile31, and Phe51 (Figure S15L, Supporting Information). To confirm binding, we performed a microscale thermophoresis assay using lysates from HEK‐293T cells transfected with GFP‐tagged CMTM4. Upon incubation of GFP‐CMTM4 lysates with ELT, a concentration‐dependent thermophoretic shift was observed with a calculated binding affinity of ≈16.84 µM, determined by non‐linear regression. This indicates a moderate affinity between CMTM4 and ELT. In contrast, no significant binding was detected in lysates from cells transfected with an empty GFP vector (Figure S15 M, Supporting Information).

To evaluate the therapeutic potential of ELT in OC, we administered ELT (30 mg kg^−1^) in a murine peritoneal tumor model. Throughout the treatment period, ELT was well‐tolerated, with no signs of acute toxicity in the liver or kidneys (Table S4, Supporting Information). Given ELT's clinical use in treating thrombocytopenia, we also measured platelet levels in ELT‐treated tumor‐bearing mice. Due to dose constraints, platelet counts in the treated mice did not return to the normal physiological levels (Table S5, Supporting Information).^[^ ^25^ ^]^ Pre‐treatment analysis confirmed that the baseline levels of abdominal tumor burden were nearly identical across all experimental groups, thereby ensuring the validity of subsequent therapeutic comparisons (Figure S15N, Supporting Information). Post‐treatment analysis demonstrated substantial tumor progression in the control group. In contrast, ELT‐treated mice exhibited a markedly reduced progression of abdominal tumor burden, although the difference did not reach statistical significance, and resembled the therapeutic efficacy observed with anti‐PD‐1 monotherapy (Figure 7J‐K). Importantly, ELT significantly strengthened the efficacy of immune checkpoint blockade (ICB) therapy. By inhibiting CMTM4, ELT synergized with PD‐1 blockade, resulting in marked tumor suppression and prolonged survival in treated mice (Figure 7L). FCM analysis of peritoneal fluid suggested that the proportion of M2 macrophages in the ELT and anti‐PD‐1 combination groups was substantially lower than that in the control and monotherapy groups (Figure S15O, Supporting Information). To extend the clinical relevance of these findings, we observed that patients with low CMTM4 expression who received anti‐PD‐1 therapy exhibited improved survival compared to those with high CMTM4 expression (Figure 7M). These results suggest that ELT inhibits tumor growth and M2 cell polarization in vivo. In conclusion, ELT acts as a small‐molecule inhibitor of CMTM4, suppressing OC growth and highlighting its potential for broader clinical applications. These findings further emphasize the critical role of CMTM4 in regulating the immune landscape within the TME, particularly through its influence on TAM phenotypes.

### 2.8 Deciphering the Clinical Implications of the CMTM4–ICAM1–M2 Macrophage Polarization Axis in OC

**Figure 8:**
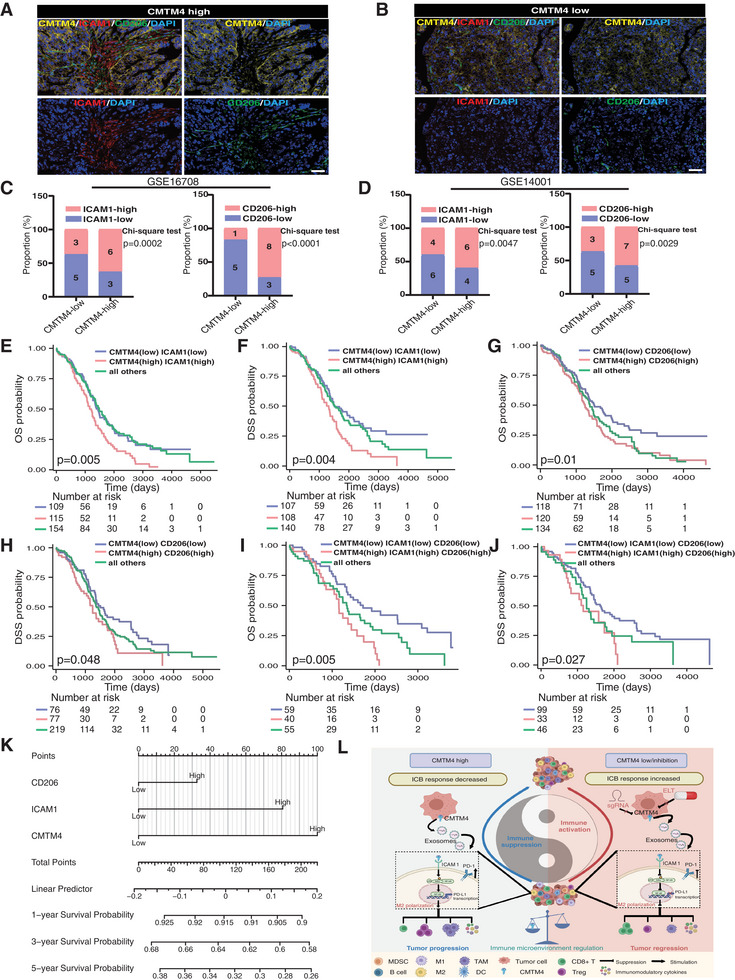
The CMTM4–ICAM1–CD206 axis as a prognostic biomarker in patients with OC. A‐B) Representative mIF images showing CMTM4 (yellow), ICAM1 (red), and CD206 (green) in tumor tissue sections with CMTM4^high^ (A) or CMTM4^low^ (B) expression from patients with OC. Scale bar, 40 µm. C‐D) Proportions of *ICAM1* (left) and *CD206* (right) mRNA expression levels in OC tissues with low or high CMTM4 expression from datasets GSE16708 (C) and GSE14001 (D). Statistical analysis was performed using the chi‐square test. E‐F) Kaplan–Meier analysis of OS and DSS in patients from the TCGA‐OC cohort with CMTM4^high^ICAM1^high^ and CMTM4^low^ICAM1^low^ expression profiles. Statistical analysis was performed using the log‐rank test. G‐H) Kaplan–Meier analysis of OS and DSS in patients from the TCGA‐OC cohort with CMTM4^high^CD206^high^ and CMTM4^low^CD206^low^ expression profiles. Statistical analysis was performed using the log‐rank test. I‐J) Kaplan–Meier analysis of OS and DSS evaluating the impact of the CMTM4–ICAM1–CD206 axis in patients from the TCGA‐OC cohort. Statistical analysis was performed using the log‐rank test. K) A nomogram was constructed based on independent prognostic factors identified in TCGA‐OC data. L) Schematic illustration depicting how exosomal CMTM4 mediates tumor cell–macrophage communication, promoting the formation of an immunosuppressive microenvironment in OC (created with MedPeer: https://www.medpeer.cn). Expansively, in ovarian tumors with high CMTM4 expression, exosomes deliver CMTM4 to macrophages, leading to ICAM1 upregulation and M2 polarization via p65 nuclear translocation. This process enhances the secretion of immunosuppressive factors and increases PD‐1 expression. In this immunosuppressive TME, macrophages diminish the proportion of CD8^+^ T cells and elevate Tregs, thereby fostering tumor progression. Conversely, strategies aimed at reducing CMTM4 expression in tumors via pharmacological agents or RNA interference trigger a robust anti‐tumor immune response, reverse the immunosuppressive TME, and consequently improve patient prognosis. All statistical analyses were performed using GraphPad Prism.

To investigate the clinical value of the CMTM4–ICAM1–M2 macrophage polarization axis in OC, we systematically assessed CMTM4 expression along with associated ICAM1 and CD206 levels in patient specimens. Multiplex IF (mIF) analysis demonstrated that elevated CMTM4 expression was significantly associated with increased infiltration of M2 macrophages and higher ICAM1 expression. A similar, albeit less pronounced, trend was observed in patients with low CMTM4 expression (**Figure** **8**A‐B). Furthermore, correlation analysis further confirmed a robust positive correlation between CMTM4 expression and both CD206‐positive macrophage infiltration and ICAM1 expression (Figure S16, Supporting Information). To validate these findings, we examined two additional datasets. Consistent with previous results, tumors exhibiting high CMTM4 expression showed elevated levels of both CD206 and ICAM1 expression (Figure 8C‐D). In conclusion, these results establish a strong association between elevated CMTM4 levels and increased M2 macrophage infiltration as well as ICAM1 expression in OC.

Moreover, patients with higher expression of CMTM4, ICAM1, or CD206 experienced markedly reduced OS and DSS compared to controls (Figure 8E‐H). Notably, patients with simultaneous elevation in CMTM4, ICAM1, and CD206‐positive macrophages had the poorest outcomes in both OS and DSS (Figure 8I‐J). Based on these findings, we constructed a prognostic nomogram incorporating these biomarkers to quantitatively predict survival outcomes in OC (Figure 8K). Figure 8L presents a graphical summary of the proposed mechanistic model and workflow of this study. Overall, the CMTM4–ICAM1–M2 macrophage polarization axis is closely associated with poor clinical outcomes and holds strong potential as a prognostic biomarker in patients with OC.

## 3 Discussion

Throughout tumor progression and metastasis, the interplay between tumor cells and immune components within the TME profoundly influences therapeutic efficacy. Among the diverse immune cell populations, TAMs have emerged as key players in fostering an immunosuppressive landscape and promoting tumor development.^[^ ^26^, ^27^, ^28^ ^]^ In this context, our study identifies exosomal CMTM4 as a critical mediator of TAM‐induced immunosuppression, highlighting its role in shaping the OC microenvironment. While previous studies have explored how various molecular signals facilitate TAM‐tumor cell interactions, the specific role of exosomal proteins—such as CMTM4—in reprogramming TAMs toward an M2‐like phenotype has remained largely unexplored. Here, we provide new insights into how exosomal CMTM4 modulates TAM polarization, activates NF‐κB signaling, and promotes the secretion of immunosuppressive cytokines and chemokines, thereby driving tumor progression and metastasis. These findings underscore the potential of targeting TAM–exosome interactions as a promising therapeutic strategy in OC.

CMTM4, a recently identified gene, has garnered increasing attention due to its pivotal role in stabilizing PD‐L1.^[^ ^29^ ^]^ However, its contribution to the TIME remains poorly characterized. Elucidating the physiological and pathological functions of CMTM4 in cancer progression is essential for developing novel immunotherapeutic strategies. In this study, through the use of comprehensive datasets, clinical specimens, and multiple tumor‐bearing animal models, we demonstrate that CMTM4 is significantly overexpressed in OC. Notably, the stark contrast between our in vitro and in vivo findings underscores the critical role of the TIME in mediating the oncogenic function of CMTM4. Analysis of TCGA‐OC cohort revealed a strong correlation between CMTM4 expression and CD68⁺ macrophage infiltration, which was further validated in both human and murine tumor samples. CMTM4 deficiency reduced TAM infiltration, and macrophage depletion phenocopied its tumor‐suppressive effects, while TAM supplementation restored tumor growth. Notably, TAM depletion in ID8/WT‐bearing mice exerted an even greater anti‐tumor effect than CMTM4 depletion, underscoring the central role of TAMs in OC progression. Moreover, CMTM4 not only increased TAM abundance but also enhanced their immunosuppressive phenotype, as evidenced by elevated Tregs and reduced CD8⁺ T cell infiltration. Collectively, these findings establish CMTM4 as a key orchestrator of the TIME, promoting immune evasion and tumor progression via TAM modulation.

TAMs are typically polarized into the M2 phenotype, which is associated with immune suppression and tumor progression. M2‐polarized TAMs promote tumor growth by creating an immunosuppressive microenvironment that impairs effective anti‐tumor immune responses.^[^ ^30^, ^31^, ^32^ ^]^ Our study further supports this concept. We found that CMTM4 not only increased TAM infiltration but also skewed their polarization toward the M2 phenotype. TCGA‐OC data confirmed a positive correlation between CMTM4 levels and M2 infiltration. Both in vitro and in vivo assays further validated that CMTM4 promotes M2 polarization. We identified exosomes as the vehicle for CMTM4 transfer from tumor cells to macrophages, where they progressively increased CMTM4 expression in recipient macrophages. Exosomes from CMTM4‐overexpressing cells promoted M2 polarization, upregulated PD‐1 and TIM3 on macrophages, and inhibited T cell activation and IFN‐γ secretion, whereas those from CMTM4‐knockout cells did not. This highlights the pivotal role of exosomal CMTM4 in driving the immunosuppressive TAM phenotype. Furthermore, CMTM4‐enriched exosomes promoted tumor migration, invasion, and peritoneal metastasis. Ablation of exosomal CMTM4 reduced TAM‐mediated immunosuppression, restored CD8⁺ T cell activity, decreased Treg infiltration, and prolonged survival in tumor‐bearing mice. Taken together, exosomal CMTM4 serves as a key mediator between tumor cells and the TIME, promoting OC progression.

We delineate the molecular mechanisms underlying these effects through RNA sequencing, revealing that the NF‐κB pathway functions as a central orchestrator of the immunosuppressive signaling cascade activated by exosomal CMTM4. ICAM‐1, a critical mediator of immune cell adhesion, is tightly regulated by NF‐κB signaling. Upon inflammatory stimulation, NF‐κB becomes activated, translocates into the nucleus and initiates the transcription of genes such as ICAM‐1, thereby enhancing immune cell adhesion and migration. In the context of cancer, sustained NF‐κB activation drives persistent ICAM‐1 expression, promoting tumor cell adhesion and metastasis.^[^ ^33^, ^34^, ^35^ ^]^ In this study, we demonstrate that ICAM1 acts as a key mediator of exosomal CMTM4‐induced M2 polarization and NF‐κB activation, both of which significantly contribute to OC progression and metastasis. Our findings further revealed that tumor‐derived exosomes carrying CMTM4 potentiate the secretion of TGF‐β1 and CXCL12 through NF‐κB activation in TAMs. Pharmacological blockade of TGF‐β1 and CXCL12 effectively mitigated the tumor‐promoting effects of CMTM4 overexpression in vivo. Consistent with previous studies, our findings highlight that enhanced secretion of TGF‐β1 and CXCL12 by TAMs induced—educated by CMTM4‐enriched exosomes—facilitates the establishment of an immunosuppressive microenvironment.

Emerging evidence has highlighted that exosomes‐derived from both cancer cells and components of the TME, carrying various molecular cargos, can profoundly modulate responses to ICB therapy. Specifically, cancer‐derived exosomes facilitate immune suppression by upregulating key immunoregulatory molecules such as PD‐L1. The presence of PD‐L1^+^ exosomes in plasma has been identified as a potential predictive biomarker for response to anti‐PD‐1 therapy.^[^ ^36^, ^37^ ^]^ In our study, depletion of exosomal CMTM4 in tumor cells led to a notable reduction in PD‐L1 expression, enhanced macrophage‐mediated phagocytosis of tumor cells, and promoted increased infiltration of effector T cells into the peritoneal cavity of mice. This intervention also improved the response to anti‐PD‐1 therapy. These findings position CMTM4 as a promising immunotherapeutic target.

The anti‐tumor efficacy of natural small‐molecule compounds has garnered increasing interest in recent years. However, no clinical inhibitors specifically targeting CMTM4 are currently available. In this study, we identified ELT as a potential small‐molecule that selectively targets CMTM4. Moreover, ELT is a naturally derived compound with demonstrated safety, showing no toxicity to the kidneys or liver. We validated the anti‐tumor activity of ELT in both in vitro and in vivo models. Notably, ELT also enhanced sensitivity to ICB therapy, significantly prolonging survival in murine models. These findings provide robust support for the clinical potential of ELT as a therapeutic agent targeting CMTM4 to impede OC progression.

In summary, this study highlights the critical role of exosomal CMTM4 expression in OC and its profound impact on modulating the TIME. Exosomal CMTM4 engages macrophages via ICAM1, enhancing cancer cell–macrophage interactions, facilitating p65 nuclear translocation, and promoting M2 polarization, thereby driving tumor progression. Our findings underscore the clinical relevance of the CMTM4–ICAM1–CD206 axis in OC and propose exosomal CMTM4 as a novel target to enhance ICB therapy. This study delineates the pivotal role of exosomal CMTM4 in intercellular communication and immune suppression, positioning it as a promising biomarker and therapeutic target in OC.

## 4 Experimental Section

### Patients and Specimens

A total of 61 primary OC tissues and 23 normal tissue samples were obtained from the Shanghai First Maternity and Infant Health Hospital. Fresh patient tissue samples were partially fixed in paraformaldehyde for subsequent immunohistochemistry, while a portion was used for protein extraction and stored at −80 °C. The remaining tissue was frozen at −80 °C for future RNA extraction. Informed consent was obtained from all patients participating in this study. Furthermore, detailed clinical information of these patients with OC was retrieved from the hospital's electronic medical record system. This study was approved by the Ethics Committee of Shanghai First Maternity and Infant Health Hospital (Approval No KS21299).

### Antibodies and Reagents

The antibodies and reagents used in this study are listed in Table S6 (Supporting Information).

### RNA Isolation, Reverse Transcription, and qPCR

Total RNA was extracted from tissues and cell lines using TRIzol reagent (TaKaRa, Japan), following the manufacturer's instructions. Reverse transcription was performed using the PrimeScript RT Master Mix kit (TaKaRa) with random primers. Real‐time qPCR was performed according to the manufacturer's instructions, with GAPDH serving as an endogenous control. Relative RNA expression was calculated using the 2^−ΔΔCT^ method. Primer sequences are provided in Table S7 (Supporting Information).

### RNA Interference, Stable Cell Line generation, and Plasmid Transfection

To construct a CMTM4 knockout, CMTM4 Single‐guide RNA (sgRNA) was cloned into the LentiCRISPRv2 vector. The sgRNAs were designed using the online Benchling CRISPR sgRNA design tool (http://www.benchling.com) (human CMTM4 sgRNA: 5′‐CACCGACTACCTGCGCGGCGCGCTC‐3′, 5′‐AAACGAGCGCGCCGCGCAGGTAGTC‐3′; mouse CMTM4 sgRNA: 5′‐CACCGCGTGCGAAGGCCTCTACTTC‐3′, 5′‐AAACGAAGTAGAGGCCTTCG‐3′). Knockout efficiency was evaluated using WB. For overexpression of human and mouse CMTM4, the full‐length open reading frame was subcloned into the pLV3 vector and pCDH vectors, respectively. si‐CMTM4, si‐ICAM1 and their respective negative controls were designed and synthesized by Sangon Biotech (Shanghai, China). siRNA transfection was performed using Lipofectamine^TM^ RNAiMAX Reagent (Invitrogen, Carlsbad, CA, USA) following the manufacturer's instructions. All sequences used in this study are summarized in Table S8 (Supporting Information).

### Flow Cytometry

Briefly, samples were processed into single‐cell suspensions and stained with antibodies on ice for 30 min. After staining, cells were washed with PBS and centrifuged at 1000 × g for 5 min. The resulting pellets were resuspended in 100 µL of PBS for analysis using a flow cytometer (FACS Calibur, BD Biosciences, Franklin Lakes, NJ, USA). Human and mouse M1 macrophages were identified as CD11b^+^ CD86^+^, while human M2 macrophages were defined as CD11b^+^ CD206^+^. Mouse macrophages/TAMs were identified as CD45^+^ F4/80^+^, mouse CD8^+^ T cells as CD45^+^ CD3^+^ CD8^+^, and Tregs as CD45^+^ CD3^+^ CD4^+^ FOXP3^+^.^[^ ^30^ ^]^

### Quantification and Statistical Analysis

Statistical analyses were performed using R (v4.2.2) or GraphPad Prism (v.9) software. Cell counts were analyzed using the ImageJ software, and FCM data were quantified using the FlowJo (v10) software. Details are provided in the figure legends. A p‐value of <0.05 was considered statistically significant.

## Conflict of Interest

The authors declare no conflict of interest.

## Author Contributions

B.Y., J.D., and J.L. contributed equally to this work and share first authorship. B.Y. and L.F.H. designed and supervised the study; B.Y., J.Y.D., and J.L. performed the experiments; LFH provided administrative, technical, and material support; H.R.H., Y.S.Z., M.Q.Y., H.J.Z., B.Y.H., T.F.H., and M.J.L. analyzed the data; B.Y., A.L., and Y.Y.H. wrote and revised the manuscript. All authors read and approved the final manuscript.

## Supporting information



Supporting Information

## Data Availability

The data that support the findings of this study are available on request from the corresponding author. The data are not publicly available due to privacy or ethical restrictions.
