# Supplementary material for: Exosomal CMTM4 Induces Immunosuppressive Macrophages to Promote Ovarian Cancer Progression and Attenuate Anti‐PD‐1 Immunotherapy
Source: Adv Sci (Weinh). 2025 May 28;12(30):e04436. doi: 10.1002/advs.202504436 (PMC12376563; doi:10.1002/advs.202504436)
Supplement: Supplementary file 1 — Supporting Information [file ADVS-12-e04436-s001.docx]

**Supplementary Materials for**

**Exosomal CMTM4 Induces Immunosuppressive Macrophages to Promote Ovarian Cancer Progression and Attenuate Anti-PD-1 Immunotherapy**

*Bo Yin****†****, Jianyi Ding****†****, Jie Liu****†****, Haoran Hu, Yashi Zhu, Meiqin Yang, Huijuan Zhou, Baoyou Huang, Tiefeng Huang, Mengjie Li, Yinyan He*, Ang Li*, Lingfei Han**

*Corresponding Author: Lingfei Han, E-mail: lingfeihan@tongji.edu.cn; Ang Li, liang@tongji.edu.cn; Yinyan He，E-mail: yinyan_he@tongji.edu.cn.

**†** These authors contributed equally to this work and share first authorship

**This file includes:**

**Supplementary Materials and Methods**

**Reference**

**Supplementary Table S1 to S8**

**Supplementary Figure S1-S16 and Figure Legends**

**Supplementary Materials and Methods**

**Cell culture and treatment**

OC cell lines A2780 (RRID:CVCL_0134), ES2 (RRID:CVCL_AX39), HEY (RRID: CVCL_0297), OVCA-429 (RRID:CVCL_3936), OVCA8 (RRID:CVCL_1629), human monocytic leukemia cells (THP1-RRID:CVCL_0006), Mouse Monocytic Macrophage Leukemia Cell Line (RAW264.7-RRID:CVCL_C6XG), jurkat cell (RRID:CVCL_0065), and human embryonic kidney 293T (HEK293T- RRID:CVCL_0063) cells were purchased from the Cell Banks of Type Culture Collection of Chinese Academy of Sciences (Shanghai, China). ID8 cell line (RRID: CVCL_IU14) was purchased from FuHeng Biology (Shanghai, China). The above cells were cultured in routinely cultured in high glucose DMEM (Gibco, USA) or 1640 (Gibco, USA) containing 10% fetal bovine serum (Gibco, USA). All cells were nurtured in a saturated humidity at 37 °C with 5% CO2. The cell lines were authenticated by short tandem repeat (STR) profiling and tested free of mycoplasma.

For THP-1 differentiation, THP-1 cells were seeded in 6-well/12-well/24-well plates at a density of 2 × 10^5^/1 × 10^5^/0.5 × 10^5^cells/mL and treated with 200 ng/mL PMA (Sigma, USA) for 48 h to polarize macrophages. Subsequently, PMA-THP-1 macrophages were cultured by the addition of IL-4 (20 ng/ml) (Peprotech, USA) or exosomes from OC cells for follow-up analysis.

**Public database analysis**

The gene expression profiles of OC were retrieved from The Cancer Genome Atlas (TCGA) or Gene Expression Omnibus (GEO) database. The Spearman correlation coefficient was determined to evaluate the CMTM4 expression and TME cell compositions in the TCGA-OC cohort. The TME matrix was calculated using the CIBERSORT algorithm. ^[1]^ Kaplan–Meier and Cox regression analyses were performed to investigate the prognosis and clinical significance of CMTM4 expression. Survival analyses are available online from the Gene Expression Profiling Interactive Analysis (GEPIA) dataset, Kaplan–Meier Plotter and TIMER2.0. Immune cell infiltration is available online from TIMER2.0. ^[2-4]^ The remaining databases are described in detail in the manuscript results section.

**Western Blotting**

Tissues and cells were lysed with ice-cold RIPA lysis buffer (NCM Biotech, China) supplemented with protease and phosphatase inhibitors. The lysates were incubated on ice for 30 min and centrifuged at 12,000 × g for 15 min at 4°C to collect the supernatant. The total protein concentration was quantified using a BCA assay kit (Thermo Fisher Scientific) according to the manufacturer’s instructions.

Equal amounts of protein were mixed with loading buffer, denatured at 95°C for 5 min, and separated by SDS-PAGE. Proteins were transferred to PVDF membranes, which were blocked with 5% non-fat milk in TBST for 1 h at room temperature. The membranes were incubated overnight at 4°C with primary antibodies specific to CMTM4, GAPDH, CD9, Calnexin, ICAM1, p-IKBα, IKBα, p-NF-κB, NF-κB, and CD206. After washing, the membranes were incubated with HRP-conjugated secondary antibodies for 1 h at room temperature. Protein bands were visualized using an enhanced chemiluminescence (ECL) detection kit and captured with a bioanalytical imaging system.

**Immunohistochemistry, immunofluorescence and Hematoxylin-eosin**

Paraffin-embedded sections from OC patient or mouse tumor tissues were used for immunohistochemistry (IHC), hematoxylin-eosin (H&E), and immunofluorescence (IF) analyses.

For H&E, Slides were deparaffinized in xylene, rehydrated through graded alcohols, and stained with hematoxylin and eosin.

For IHC, antigen retrieval was performed using Tris-EDTA buffer. Nonspecific binding was blocked with 5% bovine serum albumin (BSA). Slides were incubated with primary antibodies (CMTM4, CD44, Ki67, CD68, CD19, CD8, FOXP3), followed by horseradish peroxidase (HRP)-conjugated secondary antibodies. Diaminobenzidine (DAB) substrate was applied for visualization, and slides were counterstained with hematoxylin.

For IF, after antigen retrieval and blocking with 5% BSA, slides were incubated with primary antibodies (CMTM4, Ki67, CD44, CD68). Cy3-conjugated secondary antibodies were applied, and slides were mounted with DAPI-containing medium. Imaging was performed using a fluorescence microscope (Olympus).

**Proliferation, migration and invasion assays**

5-ethynyl-2’-deoxyuridine (EdU, CX002, Epizyme Biotech, Shanghai) assay was used to detect cell proliferation. Appropriate amounts of cell from different treatment groups were inoculated in 12-well plates and imaged with fluorescence microscope (Olympus) at specified time. The transwell chamber (3422, Corning, USA) is used to measure the ability of cells to migrate and invade. OC cells (1 × 10^5^, 200 μL) were inoculated in the upper chamber with serum-free medium and the lower chamber was filled with medium containing 10% FBS. After 24 h, the cells on the membrane of transwell inserts were fixed with 4% paraformaldehyde and stained with 0.1% crystal violet. The counts of migrated cells were quantified using the ImageJ software.

**Cell Apoptosis Assay**

Cells were collected and washed twice with cold PBS. The apoptosis assay was performed using an Annexin V-FITC/PI apoptosis detection kit according to the manufacturer’s instructions. Briefly, cells were resuspended in binding buffer and stained with Annexin V-FITC and propidium iodide (PI) for 15 min at room temperature in the dark. After staining, samples were analyzed using FCM. The fluorescence signals of Annexin V and PI were detected in the FITC and PE channels, respectively. Data analysis was performed to quantify apoptotic cells.

**Establishment of ID8-luciferase (ID8-luc) cells**

Luciferase-expressing ID8 cells (ID8-luc) were established by transducing murine ovarian cancer ID8 cells with a lentiviral vector encoding the firefly luciferase gene. The lentivirus was purchased from GeneChem Co., Ltd. (Shanghai, China). After 48 h, the culture medium was replaced, and G-418 (MedChemExpress, China) was added at a concentration of 500 μg/mL for 10 days to select stably transduced cells. Successful luciferase expression was confirmed by bioluminescence imaging using D-luciferin substrate.

**Animal experiment**

All in vivo experiments were carried out in accordance with the requirements of the Laboratory Animal Research Center of Tongji University and approved by the Animal Care Committee of Tongji University (Approval No TJBA03524101). The endpoint for all animal experiments was defined as follows: mice were euthanized if their abdominal circumference exceeded 1.4 times the initial measurement, body weight increased to more than 1.3 times the baseline weight, or signs of cachexia were observed during the experiment.

For syngeneic intraovarian model, female C57BL/6 mice (6–8 weeks old) were anesthetized with isoflurane, and a dorsal incision was made to expose the ovaries. ID8 cells (1 × 10⁶) were injected into the left ovarian sac. Mice were weighed weekly, and tumor size was assessed upon reaching the experimental endpoint. Tumor volume was calculated using the formula (L × W²)/2, where L represents the length and W the width, measured with a digital caliper.

In the i.p. model, 2.0 × 10⁶/3.0 × 10⁶ ID8 tumor cells were injected intraperitoneally. Mice were euthanized and analyzed once the experimental endpoint was reached. Body weight was recorded at indicated times.

For the i.p. treatment model in mice, 3 × 10^6^ ID8-luc cells were injected into the peritoneal cavity for chemotherapy or in vivo antibody therapy treatment followed the schedule shown in the chart of the paper. 3 × 10^6^ ID8-luc (OE-CMTM4) cells were injected into the peritoneal cavity of mice for vactosertib and AMD3100 treatment.

**In** **vivo bioluminescence imaging**

To monitor tumor progression and therapeutic response in vivo, bioluminescence imaging was performed using an in vivo imaging system (Tanon ABL-X6, Shanghai, China). Mice were anesthetized with isoflurane and injected intraperitoneally with D-luciferin (3mg/mouse) 10 min prior to imaging. Images were acquired with an exposure time of 1–3 min depending on signal intensity, and data were analyzed using Tanon Image Analysis Software. The bioluminescent signal was quantified as average flux (photons/sec/cm^2^/sr) within a region of interest (ROI) over the tumor area. All imaging procedures were conducted under consistent settings and environmental conditions.

**In vivo exosomes injection and tumorigenicity assay**

To evaluate the impact of exosomes and exosomal CMTM4 on OC progression, female mice were randomly assigned to five groups (n = 5): the PBS group, the wild-type exosomes group (WT-Exos), the CMTM4-knockout exosomes group (Exos^CMTM4 KO^), the CMTM4 overexpression control group (Exos^OE-NC^), and the CMTM4 overexpression group (Exos^OE-CMTM4^). Tumor cells and macrophages were co-injected intraperitoneally at a 1:10 ratio. Subsequently, 100 µL of PBS or 40 µg/100 µL of exosomes (respectively from ID8, ID8^CMTM4 KO^, ID8^OE-NC^, ID8^OE-CMTM4^) were administered intraperitoneally injection. Exosomes treatments were repeated weekly for a total of three doses. Tumor metastasis was ultimately assessed using vivo bioluminescence imaging system.

**Exosome uptake assay**

CM-Dil, a red fluorescent dye (Invitrogen, USA), was employed to label exosomes and track their uptake by macrophages. Confocal microscopy was then used to analyze the samples 24 h after CM-Dil treatment.

**Exosome isolation and characterization**

Equal amounts of OC cells were plated in 150 mm culture dishes and allowed to grow until reaching 70% confluency. The cells were then washed with PBS and cultured in exosome-depleted medium for 48-72 h. Conditioned media of equal volume were collected, centrifuged at 3,000 rpm for 10 minutes, and then at 10,000 × g for 30 min at 4°C to remove cell debris. The supernatants were filtered through 0.22 μm membrane filters, and exosomes were isolated by ultracentrifugation at 100,000 × g for 90 min, followed by resuspension in 100 μL of PBS.

Exosome characterization was performed using transmission electron microscopy (TEM) and nanoparticle tracking analysis (NTA). For TEM, exosome samples were placed on copper grids, stained with 2% uranyl acetate, and air-dried before imaging to confirm their morphology and size. NTA was employed to determine the size distribution and concentration of exosomes, with samples diluted in PBS to an appropriate concentration and analyzed according to the manufacturer’s instructions.

**RNA sequencing**

RNA sequencing (RNA-seq) of macrophages was performed by Shanghai OE Biotech Co., Ltd. Total RNA was extracted, and RNA quality was assessed to ensure integrity and purity. Library construction was carried out using a standardized protocol, followed by high-throughput sequencing. The resulting raw data were processed for quality control, alignment, and differential expression analysis.

**Enzyme-Linked Immunosorbent Assay (ELISA)**

TGF-β1 and CXCL12 levels were measured using ELISA kits from UpinBio (Zhejiang, China), following the manufacturer’s instructions. Briefly, samples and standards were added to the precoated ELISA plates and incubated. After washing, the detection antibody was added, followed by the substrate solution. The reaction was stopped, and absorbance was measured at the specified wavelength using a microplate reader. The concentrations of TGF-β1 and CXCL12 were determined based on the standard curve.

**Evaluation of IC_50_**

The IC_50_ value of the drug was determined using the CCK-8 assay. Cells were seeded into 96-well plates and treated with different concentrations of the drug for 48 h. CCK-8 solution was added to each well and incubated for 2 h at 37°C. Absorbance at 450 nm was measured using a microplate reader, and the IC_50_ value was calculated using a dose-response curve.

**Molecular docking and virtual screening analysis**

The structure of the CMTM4 protein was obtained from the AlphaFold database (based on the UniProt ID: Q8CJ61) and preprocessed in Discovery Studio 2019 Client by removing water molecules and native ligands, followed by grid box definition.^[5, 6]^ Hydrogens were added using AutoDockTools 1.5.6, and receptors were saved in pdbqt format. Ligands were downloaded from the Zinc database (https://zinc.docking.org/), optimized to their lowest-energy conformations using the MMFF94 force field in Open Babel 2.4.1, and converted to pdbqt format after hydrogen addition and rotatable bond assignment via AutoDockTools.^[7, 8]^ Molecular docking was carried out using AutoDock Vina 1.1.2 with parameters set as: exhaustiveness = 8, energy_range = 4 kcal/mol, and maximum binding modes = 10. The conformation with the highest absolute binding energy was selected and analyzed using Discovery Studio. Binding affinity was assessed based on binding energy, where a higher absolute value indicates stronger affinity. The top ten complexes with the highest absolute binding energies were selected for further analysis. In the visualized docking results, dashed lines represent binding interactions; residue names are annotated as letter–number codes, while pure numbers indicate bond lengths.

**Jurkat cell activation assays**

For activation, Jurkat cells were seeded into 12-well plates at a density of 1 × 10⁶ cells per well and stimulated with 25 μL of ImmunoCult™ Human CD3/CD28 T Cell Activator (STEMCELL, Canada) for 24 h. After stimulation, the cells were co-cultured with macrophages for subsequent analysis. Expression of activation markers such as CD69 and IFN-γ was evaluated by flow cytometry.

**MST Assay**

HEK-293T cells were transfected with GFP-tagged OC-CMTM4 plasmid or GFP-tagged OE-NC (negative control) using Lipofectamine^TM^ RNAiMAX Reagent following the manufacturer’s instructions. After 48 h, cells were harvested and lysed in MST-optimized lysis buffer supplemented with protease inhibitors. The lysates were clarified by centrifugation and the supernatant was collected for MST analysis. A serial dilution of ELT (in DMSO) was prepared and incubated with a constant concentration of the lysate at room temperature for 10 min. The samples were then loaded into standard treated capillaries (Nano Temper Technologies) and subjected to MST analysis. Binding interactions were monitored using the Monolith NT.115 instrument with an LED/excitation power of 20% and MST power of 40%. The dissociation constant (K_d_) was calculated using MO. Affinity Analysis software (NanoTemper Technologies), and the binding curves were plotted using GraphPad Prism. All experiments were conducted in triplicate to ensure reproducibility.

**Multiplex IF**

Tissue slides were processed following the manufacturer’s protocol for the TSA kit. Primary antibodies against CMTM4, ICAM1, and CD206 were incubated overnight at 4°C in a humidified chamber. After washing with PBS, slides were treated with enzyme-labeled secondary antibodies and incubated for 1 h at room temperature. DAPI solution was then applied for nuclear staining at room temperature. Imaging was performed using the PanoVIEW VS200 slide scanner (Panovue) equipped with an Olympus 20x objective.

**Macrophage phagocytosis assays**

In an in vitro macrophage phagocytosis assay, 1 × 10^5^ macrophages were labeled with the CD11b antibody and seeded in transparent 96-well plates. Separately, 1 × 10^5^ tumor cells were labeled with a 5 μM green fluorescent probe, CFSE (BD Biosciences). The macrophages and tumor cells were co-cultured for 4 h. The results were analyzed using FCM.

**Supplementary Table S1 to S8**

**Supplementary Table S1** TCGA Cancer Abbreviations and Full Names

| Abbreviations | Full titles |
| --- | --- |
| BRCA | Breast Invasive Carcinoma |
| CESC | Cervical Squamous Cell Carcinoma and Endocervical Adenocarcinoma |
| COAD | Colon Adenocarcinoma |
| HNSC | Head and Neck Squamous Cell Carcinoma |
| LUAD | Lung Adenocarcinoma |
| LUSC | Lung Squamous Cell Carcinoma |
| OV | Ovarian Serous Cystadenocarcinoma |
| READ | Rectum Adenocarcinoma |
| UCEC | Uterine Corpus Endometrial Carcinoma |

**Supplementary Table S2** Univariate and multivariate analyses of OS in TCGA-OC

| Characteristics | | Total(N) | | Univariate analysis | |  | Multivariate analysis | |
| --- | --- | --- | --- | --- | --- | --- | --- | --- |
|  |  |  |  | Hazard ratio (95% CI) | P value |  | Hazard ratio (95% CI) | P value |
| CMTM4 | | 379 | | 1.211 (1.038 - 1.413) | 0.015 |  | 1.136 (0.965 - 1.338) | 0.125 |
| Histologic grade | | 367 | |  |  |  |  |  |
| G2 | | 45 | | Reference |  |  |  |  |
| G3 | 322 | | | 1.234 (0.834 - 1.826) | 0.293 |  |  |  |
| Clinical stage | 375 | | |  |  |  |  |  |
| Stage II | 23 | | | Reference |  |  | Reference |  |
| Stage III&Stage IV | 352 | | | 2.519 (1.037 - 6.120) | 0.041 |  | 2.060 (0.764 - 5.558) | 0.153 |
| Venous invasion | 105 | |  | |  |  |  |  |
| No | 41 | | Reference | |  |  |  |  |
| Yes | 64 | | 0.896 (0.487 - 1.649) | | 0.723 |  |  |  |
| Lymphatic invasion | 148 | |  | |  |  |  |  |
| No | 48 | | Reference | |  |  |  |  |
| Yes | 100 | | 1.413 (0.833 - 2.396) | | 0.200 |  |  |  |
| Age | 379 | |  | |  |  |  |  |
| <= 60 | 207 | | Reference | |  |  | Reference |  |
| > 60 | 172 | | 1.352 (1.045 - 1.749) | | 0.022 |  | 1.341 (1.018 - 1.765) | 0.037 |
| Tumor status | 337 | |  | |  |  |  |  |
| Tumor free | 72 | | Reference | |  |  | Reference |  |
| With tumor | 265 | | 9.598 (4.487 - 20.532) | | < 0.001 |  | 9.148 (4.276 - 19.568) | < 0.001 |

**Supplementary Table S3** Small-molecule compounds

| Name | Formula | Molecular Weight | Docking core |
| --- | --- | --- | --- |
| Saquinavir | C_38_H_50_N_6_O_5_ | 670.84 | -12.1 |
| Nilotinib | C_28_H_22_F_3_N_7_O | 529.52 | -12.1 |
| Telmisartan | C_33_H_30_N_4_O_2_ | 514.62 | -12 |
| Dutasteride | C_27_H_30_F_6_N_2_O_2_ | 528.53 | -12 |
| Rolapitant | C_25_H_26_F_6_N_2_O_2_ | 500.48 | -11.7 |
| Zafirlukast | C_31_H_33_N_3_O_6_S | 575.68 | -11.4 |
| Lumacaftor | C_24_H_18_F_2_N_2_O_5_ | 452.41 | -11.3 |
| Eltrombopag | C_25_H_22_N_4_O_4_ | 442.47 | -11.1 |
| Conivaptan | C_32_H_26_N_4_O_2_ | 498.57 | -11 |
| Ponatinib | C_29_H_27_F_3_N_6_O | 532.56 | -11 |

**Supplementary Table S4** Effects of i.p injection of ELT on key liver and renal function indices in mice: Supplementary Table to Figure 7

| Biochemical examination | ELT treatment  (Average ± SEM) | Reference range |
| --- | --- | --- |
| ALT (U/L) | 66.47 ± 7.5 | 10.06 - 96.47 |
| AST (U/L) | 245.57 ± 22.13 | 36.31 -235.48 |
| ALP (U/L) | 20.29 ± 4.29 | 22.52 - 474.35 |
| ALB (g/L) | 29.22 ± 2.06 | 21.22 - 39.15 |
| DBIL (μmol/L) | 0.71 ± 0.07 | 0.45 - 33.89 |
| UREA (mmol/L) | 7.27 ± 0.41 | 7.1 - 18.9 |
| BUN (mg/dL) | 20.35 ± 1.05 | 10.81 - 34.74 |
| CREA (μmol/L) | 15.22 ± 2.37 | 10.91 - 85.09 |
| UA (μmol/L) | 68.76 ± 19.32 | 4.42 - 224.77 |

ALT: alanine transaminase; AST: aspartate transaminase; ALP: alakline phosphatase; ALB: albumin; DBIL: direct bilirubin; BUN: Blood Urea Nitrogen; CREA: creatinine; UA: Uric Acid

**Supplementary Table S5** Impact of i.p. ELT injection on complete blood count parameters in mice: Supplementary Table for Figure 7

| Parameter | Result | Unit | Reference range |
| --- | --- | --- | --- |
| WBC | 2.63 ± 0.48 | 10^9^/L | 0.8 - 10.6 |
| Lymph | 1.80 ± 0.35 | 10^9^/L | 0.6 - 8.9 |
| Mon | 0.13 ± 0.03 | 10^9^/L | 0.04 - 1.4 |
| Gran | 0.70 ± 0.15 | 10^9^/L | 0.23 - 3.6 |
| Lymph | 68.53 ± 1.81 | % | 40 - 92 |
| Mon | 5.13 ± 0.37 | % | 0.9 - 18 |
| Gran | 26.3 ± 0.89 | % | 6.5 - 50 |
| RBC | 8.41 ± 0.29 | 10^12^/L | 6.5 - 11.5 |
| HGB | 137 ± 5.72 | g/L | 110 - 165 |
| HCT | 38.87 ± 1.86 | % | 35 - 55 |
| MCV | 46.17 ± 0.66 | fL | 41 - 55 |
| MCH | 16.23 ± 0.20 | pg | 13 - 18 |
| MCHC | 352.33 ± 0.35 | g/L | 300 - 360 |
| RDW | 18.37 ± 1.56 | % | 12 - 19 |
| PLT | 215 ± 50.1 | 10^9^/L | 400 - 1600 |
| MPV | 6.43 ± 0.10 | fL | 4.0 - 6.2 |
| PDW | 16.67 ± 0.14 | / | 12.0 - 17.5 |
| PCT | 0.14 ± 0.036 | % | 0.100 - 0.780 |

WBC: white blood cell; Lymph: lymphocyte; Mon: monocyte; Gran: granulocyte; RBC: red blood cell; HGB: hemoglobin; HCT: hematocrit; MCV: mean corpuscular volume; MCH: mean corpuscular hemoglobin; MCHC: mean corpuscular hemoglobin concentration; RDW: red blood cell distribution width; PLT: platelet count; MPV: mean platelet volume; PDW: platelet distribution width; PCT: plateletcrit

**Supplementary Table S6** Antibodies and Reagents

| **Antibodies** | **Source** | **Identifier** | **Application** |
| --- | --- | --- | --- |
| anti-CMTM4 | Cell Signaling Technology | Cat#17433 | WB (1:1000) |
| anti-CMTM4 | Sigma | HPA014704 | IHC (1:200)  IF (1:100) |
| anti-GAPDH | NCM Biotech | Cat#AB2100 | WB (1:20000) |
| anti-CD9 | Cell Signaling Technology | Cat#13174 | WB (1:1000) |
| anti- Calnexin | Cell Signaling Technology | Cat#2433 | WB (1:1000) |
| anti-ICAM1 | Santa Cruz | Cat#sc-390483 | WB (1:2000)  IF (1:50) |
| anti-p-IKBα | Abmart | Cat#TP56280 | WB (1:1000) |
| anti- IKBα | Abmart | Cat#T55026 | WB (1:1000) |
| anti-p-NF-κB | Abmart | Cat#TP56372 | WB (1:1000) |
| anti-NF-κB | Abmart | Cat#T55034 | WB (1:1000) |
| anti-NF-κB | ABclonal | Cat#A19653 | IF (1:100) |
| anti-CD206 | Santa Cruz | Cat#sc-376232 | WB (1:500)  IF (1:50) |
| anti-CD44 | ABclonal | Cat#A21919 | IHC (1:200)  IF (1:100) |
| anti-Ki67 | ABclonal | Cat#A20018 | IHC (1:200)  IF (1:100) |
| anti-CD68 | Cell Signaling Technology | Cat#97778 | IHC (1:500)  IF (1:200) |
| anti-CD19 | ABclonal | Cat#A19013 | IHC (1:200) |
| anti-CD8 | ABclonal | Cat#A23305 | IHC (1:200) |
| anti-FOXP3 | Cell Signaling Technology | Cat#12653 | IHC (1:300) |
| Zombie Aqua™ Fixable Viability Kit | Biolegend | Cat#423101 | FCM |
| Brilliant Violet 421™ anti-mouse CD45 | Biolegend | Cat#103134 | FCM |
| FITC anti-mouse CD3 | Biolegend | Cat#100203 | FCM |
| PerCP/Cyanine5.5 anti-mouse CD8a | Biolegend | Cat#162310 | FCM |
| PerCP/Cyanine5.5 anti-mouse F4/80 | Biolegend | Cat#123128 | FCM |
| PE Anti-Mouse CD206/MMR | Elabscience | Cat# E-AB-F1135D | FCM |
| APC Anti-Mouse CD4 | Elabscience | Cat# E-AB-F1097E | FCM |
| PE Anti-Mouse Foxp3 | Elabscience | Cat# E-AB-F1238D | FCM |
| PE/Cyanine7 anti-human CD69 | Biolegend | Cat# 310912 | FCM |
| APC anti-human IFN-γ | Biolegend | Cat#986702 | FCM |
| FITC anti-human CD279 (PD-1) | Biolegend | Cat#379205 | FCM |
| APC anti-human CD206/MMR | Biolegend | Cat#321110 | FCM |
| PE anti-human CD86 | Biolegend | Cat#374206 | FCM |
| FITC anti-human CD11b | Biolegend | Cat#982614 | FCM |
| APC anti-mouse/human CD11b | Biolegend | Cat#101212 | FCM |
| Brilliant Violet 421™ anti-human CD274 (B7-H1, PD-L1) | Biolegend | Cat#329714 | FCM |
| PE anti-human CD47 | Biolegend | Cat#323108 | FCM |

WB: western blotting; IF: immunofluorescence; IHC: immunohistochemistry; FCM: flow cytometry.

| **Reagents** | **Source** | **Identifier** |
| --- | --- | --- |
| InVivoMAb anti-mouse F4/80 | BioXCell | Cat#BE0206 |
| InVivoMAb anti-mouse PD-1 (CD279) | BioXCell | Cat#BE0146 |
| ImmunoCult™ Human CD3/CD28 T Cell Activator | STEMCELL Technologies | Cat#10971 |
| CellTracker™ CM-Dil | Invitrogen | Cat#C7001 |
| FITC Phalloidin | Yeasen Biotechnology | Cat#40735ES75 |
| Pyrrolidinedithiocarbamate ammonium | MedChemExpress | Cat#HY-18738 |
| G-418 | MedChemExpress | Cat#HY-17561 |
| TGF beta 1/TGFB1 Protein, Human | MedChemExpress | Cat#HY-P7118 |
| SDF-1 alpha/CXCL12 Protein, Human | MedChemExpress | Cat#HY-P70469 |
| SDF-1 alpha/CXCL12 Protein, Mouse | MedChemExpress | Cat#HY-P7285 |
| Vactosertib | MedChemExpress | Cat#HY-19928 |
| Plerixafor (AMD3100) | MedChemExpress | Cat#HY-10046 |
| Cisplatin (CDDP) | MedChemExpress | Cat#HY-17394 |
| Paclitaxel | MedChemExpress | Cat#HY-B0015 |
| BD Horizon™ CFSE | BD Biosciences | Cat#565082 |
| D-Luciferin potassium salt | Aladdin | Cat#L120798 |
| Saquinavir | MedChemExpress | Cat#HY-17007 |
| Nilotinib | MedChemExpress | Cat#HY-10159 |
| Telmisartan | MedChemExpress | Cat#HY-13955 |
| Dutasteride | MedChemExpress | Cat#HY-13613 |
| Rolapitant | MedChemExpress | Cat#HY-14751 |
| Zafirlukast | MedChemExpress | Cat#HY-13613 |
| Lumacaftor | MedChemExpress | Cat#HY-17492 |
| Eltrombopag | MedChemExpress | Cat#HY-15306 |
| Conivaptan | MedChemExpress | Cat#HY-18347 |
| Ponatinib | MedChemExpress | Cat#HY-12047 |

**Supplementary Table S7 qPCR primer list**

| Gene name | Species | Forward Sequence | Reverse Sequence |
| --- | --- | --- | --- |
| CMTM4 | Human | CTGCCGTGATATTTGGCTTCT | CGGATGTAGTCATTGGTGCTCT |
| GAPDH | Human | AAAGGCATTCTTCACCTGCTCC | GCCATCACGCCACAGTTTC |
| CD206 | Human | GGGTTGCTATCACTCTCTATGC | TTTCTTGTCTGTTGCCGTAGTT |
| CD163 | Human | TTTGTCAACTTGAGTCCCTTCAC | TCCCGCTACACTTGTTTTCAC |
| IL-10 | Human | CTGAGAACCAAGACCCAGACA | AAAGGCATTCTTCACCTGCTCC |
| IL-4 | Human | CTGCTTCCCCCTCTGTTCTTC | TGATATCGCACTTGTGTCCGTG |
| ARG1 | Human | TGACGGACTGGACCCATCTT | GGCTTGTGATTACCCTCCCG |
| VEGF | Human | GTGCCCACTGAGGAGTCCA | GTGCTGGCCTTGGTGAGGT |
| TGFB1 | Human | CACTCTCAAACCTTTACGAGACC | CGTTGCTAGGGGCGAAGATG |
| CXCL12 | Human | CCGCGCTCTGCCTCAGCGACGGGAAG | CTTGTTTAAAGCTTTCTCCAGGTACT |
| ICAM1 | Human | GTCACCTATGGCAACGACTCCTTC | AGTGTCTCCTGGCTCTGGTTCC |
| PD-1 | Human | CCAGGATGGTTCTTAGACTCCC | TTTAGCACGAAGCTCTCCGAT |
| PD-L1 | Human | TGGCATTTGCTGAACGCATTT | TGCAGCCAGGTCTAATTGTTTT |
| CTLA-4 | Human | GTGAACCTCACTATCCAAGGAC | TGCCTATGCCCAGGTAGTA |
| LAG-3 | Human | GCGGGGACTTCTCGCTATG | GGCTCTGAGAGATCCTGGGG |
| TIM-3 | Human | AGACAGTGGGATCTACTGCTG | CCTGGTGGTAAGCATCCTTGG |
| GADD45B | Human | TACGAGTCGGCCAAGTTGATG | GGATGAGCGTGAAGTGGATTT |
| PRKCB | Human | AGCCCCACGTTTTGTGACC | GCTGGGAACATTCATCACGC |
| ZAP70 | Human | ACGCCAAGATCAGCGACTTT | GGGTGCGTACCACTTGAGC |
| LAT | Human | GATGAGGACGACTATCACAACCC | GAAGGCACTGTCTCGGATGC |
| XIAP | Human | ACCGTGCGGTGCTTTAGTT | TGCGTGGCACTATTTTCAAGATA |
| MALT1 | Human | CGCCTCAGTTGCCTAGACC | TCACCCATTAACTTCAGCAGAC |
| UBE2I | Human | AAAAATCCCGATGGCACGATG | CTTCCCACGGAGTCCCTTTC |
| TAB2 | Human | GCCACCAAATTGATTTTCAGGTT | TGCGTAGACCAGAAATTCCAGA |
| CXCL2 | Human | GCTTGTCTCAACCCCGCATC | TGGATTTGCCATTTTTCAGCATCTT |

**Supplementary Table S8 The sequences for siRNA**

| Gene | Primer | Sequence |
| --- | --- | --- |
| CMTM4-1 | Sense | CCGUGAUAUUUGGCUUCUUTT |
|  | Antisense | AAGAAGCCAAAUAUCACGGTT |
| CMTM4-2 | Sense | GCAUAUGCAGUGAACACAUTT |
|  | Antisense | AUGUGUUCACUGCAUAUGCTT |
| ICAM1-1 | Sense | GUAUGAACUGAGCAAUGUGCATT |
|  | Antisense | UGCACAUUGCUCAGUUCAUACTT |
| ICAM1-2 | Sense | GCUAAAACCUUCCUCACCGUGTT |
|  | Antisense | CACGGUGAGGAAGGUUUUAGCTT |

**Supplementary Figure S1-S16 and Figure Legends**

**Fig. S1**


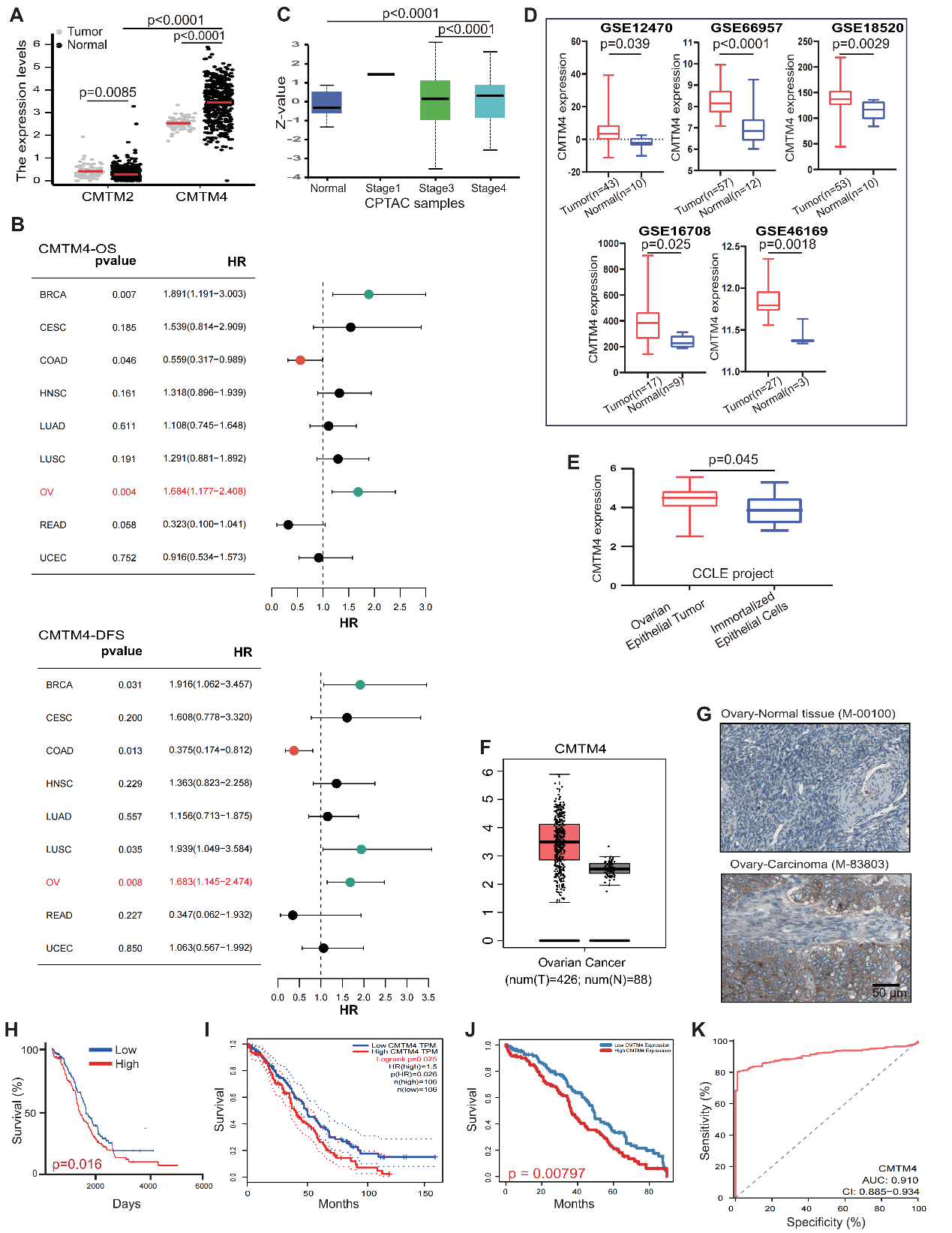


**Fig. S1** Expression and clinical significance of CMTM4 in OC.

(A) CMTM4 and CMTM2 expression are evaluated in cancer tissues compared to normal tissues based TCGA-OC. Data are shown as mean; unpaired two-sided Student's t-test (compared to normal control).

(B) Forest maps from TCGA data showed the hazard ratios (HR) of CMTM4 expression to OS (up) and DFS (down) in different cancers.

(C) The validation of CMTM4 overexpression in human OC tissues from the CPTAC cohort.

(D) The expression of CMTM4 in tumor samples and normal samples was analyzed in five GEO datasets (GSE12470, GSE66957, GSE18520, GSE16708, GSE46169). Data are shown as mean ± SEM; unpaired two-sided Student's t-test (compared to normal control).

(E) The expression of CMTM4 in epithelial OC cell lines and normal immortalized cell lines was examined using the CCLE database (<https://sites.broadinstitute.org/ccle>). Data are shown as mean ± SEM; unpaired two-sided Student's t-test.

(F-G) Further validation of CMTM4 overexpression in OC tissues through GEPIA(F) (gepia.cancer-pku.cn) and HPA (G) (https://www.proteinatlas.org/) databases.

(H-J) Further validation supported the association between high CMTM4 expression and an adverse prognosis through OncoLnc (H) (www.OncoLnc.org), TIMER2.0 (I) (timer.cistrome.org) and GEPIA (J). Statistical analysis was performed using the log-rank test.

(K) The area under the curve (AUC) indicates the accuracy of CMTM4 as a biomarker.

For A, D, E, all statistical analyses were using GraphPad Prism.

**Fig. S2**


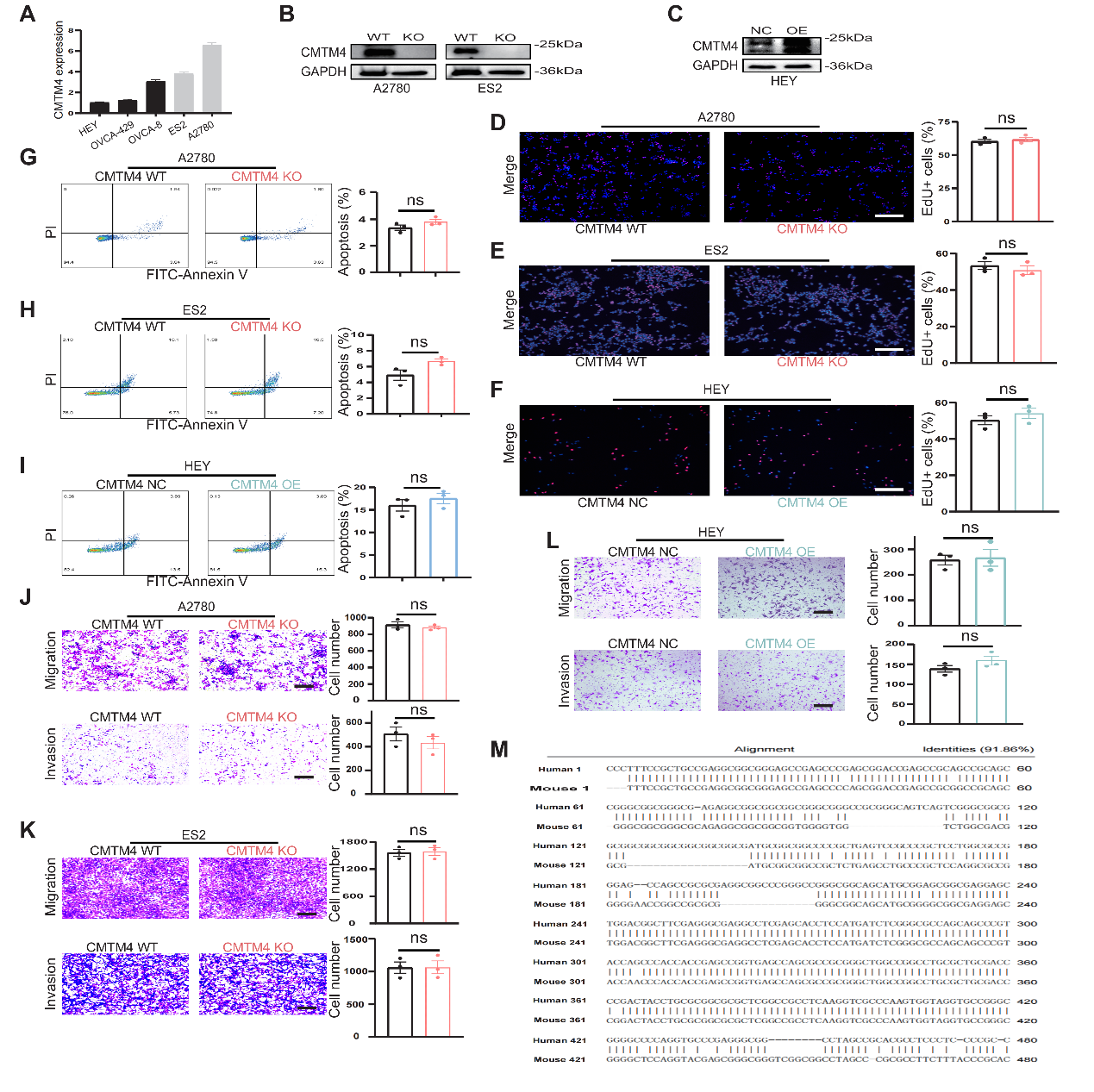


**Fig. S2** The change of CMTM4 expression did not affect the biological function of OC in vitro.

(A) qPCR was performed to detected the mRNA expression of *CMTM4* in HEY, OVCA-429, OVCA-8, ES2 and A2780 cell lines. Data are shown as mean ± SD (n = 3 independent experiments per group).

(B) WB showed that the knockout efficiency of CMTM4 sgRNA on A2780 and ES2. Representative WB images are shown (n = 3 independent experiments per group).

(C) WB analysis demonstrated the successful establishment of CMTM4-overexpressing HEY cell lines. Representative WB images are shown (n = 3 independent experiments per group).

(D-F) The proliferation of A2780(D), ES2(E) and HEY(F) cells as indicated treatments were evaluated using EdU assays, respectively. Scale bar, 100 μm. Representative cell images are shown. Data are shown as mean ± SD (n = 3 independent experiments per group). ns, not significant.

(G-I) The apoptosis of A2780 (G), ES2 (H) and HEY (I) cells as described treatments was studied by FCM, respectively. Representative results are shown. Data are shown as mean ± SD (n = 3 independent experiments per group). ns, not significant.

(J-L) The migration and invasion of A2780 (J), ES2 (K) and HEY (L) cells according to the treatments applied were demonstrated using Transwell assays, respectively. Scale bar, 100 μm. Representative cell images are shown. Data are shown as mean ± SD (n = 3 independent experiments per group). ns, not significant.

(M) Sequence alignment identification of human and mouse CMTM4.

All statistical analyses were using GraphPad Prism, cells were quantified using ImageJ software, and FCM results were analyzed by FlowJo.

**Fig. S3**


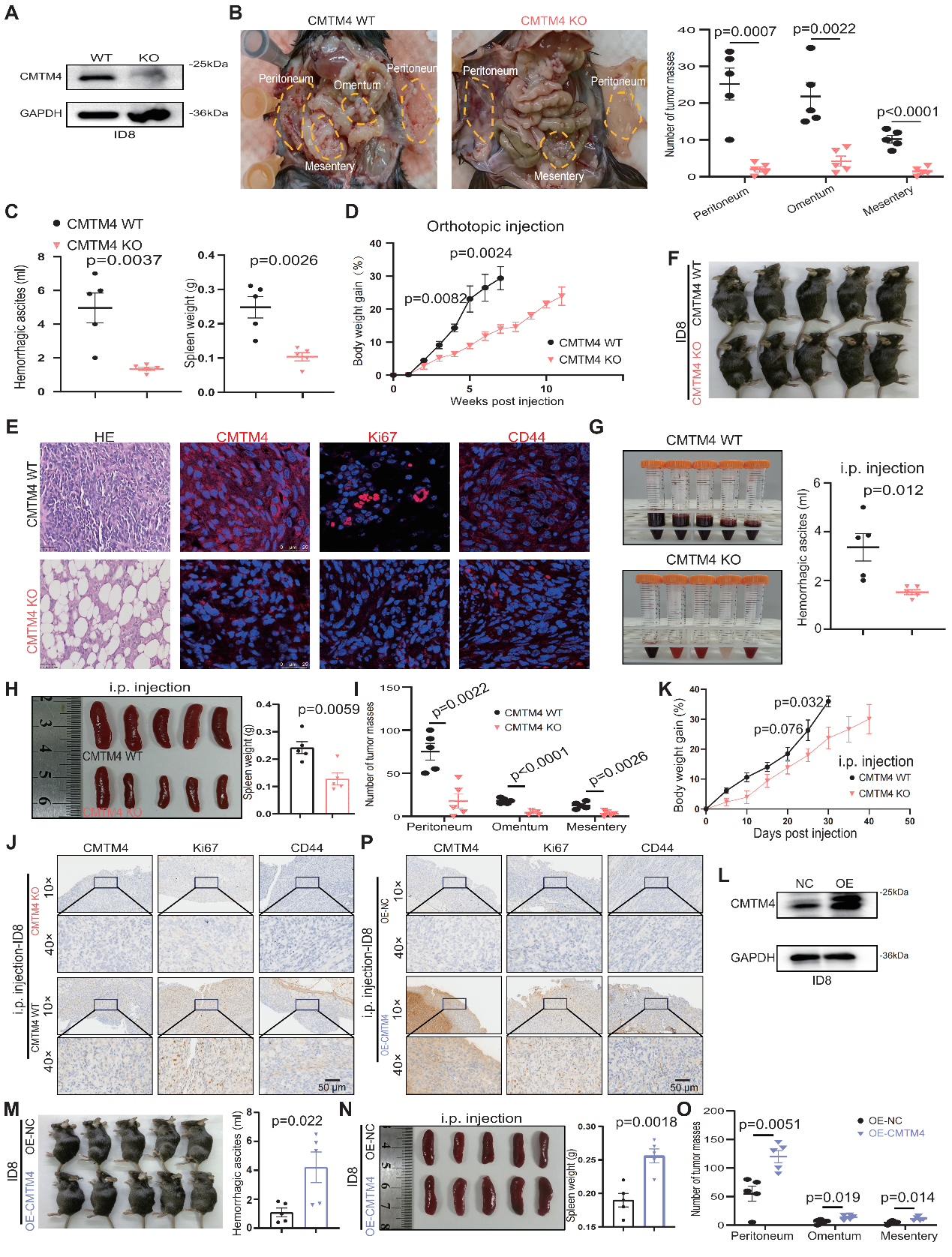


**Fig. S3** The loss of CMTM4 slows down the progression of OC in vivo.

(A) CMTM4 deletion in ID8 cells was confirmed by WB. Representative WB images are shown (n = 3 independent experiments per group).

(B-E) An orthotopic syngeneic mouse model was created by injecting 1 × 10^6^ ID8 cells into C57BL/6 mice (n = 5 per group).

(B) Representative images (ID8/WT vs. ID8/CMTM4 KO) of tumor implantations in peritoneum, omentum and mesentery. Data are presented as the means ± SEM (n = 5 per group); unpaired two-sided Student's t-test (compared to CMTM4 KO group).

(C) Comparison and quantification of abdominal hemorrhagic ascites volume (left) and spleen weight (right) in two groups of mice. Data are presented as the means ± SEM (n = 5 per group); unpaired two-sided Student's t-test (compared to CMTM4 KO group).

(D) Weekly body weight recording and ascites volume as percentage of weight gain between groups. Data are presented as the means ± SEM (n = 5 per group); unpaired two-sided Student's t-test (compared to CMTM4 KO group).

(E) H&E and IF staining images of CMTM4, Ki67 and CD44 in mouse tumor sections (ID8/WT vs. ID8/CMTM4 KO). Typical images are shown. (HE Scale bar, 50 μm and IF Scale bar 25 μm).

(F-K) I.p. tumor model injected with 3 × 10^6^ ID8 cells (n = 5 mice per group).

(F) When the ID8/WT-bearing mice reached the end point, all the mice were euthanized.

(G) The hemorrhagic ascites of euthanized mice was collected and compared quantitatively. Data are presented as the mean ± SEM (n = 5 per group); unpaired two-sided Student's t-test (compared to CMTM4 KO group).

(H) Spleen images were exhibited and weighed comparison between groups. Data are presented as the mean ± SEM (n = 5 per group); unpaired two-sided Student's t-test (compared to CMTM4 KO group).

(I) In the i.p. tumor-bearing mouse model, metastatic nodules in peritoneum, omentum and mesentery were quantified. Data are presented as the mean ± SEM (n = 5 per group); unpaired two-sided Student's t-test (compared to CMTM4 KO group).

(J) In the i.p. tumor-bearing mouse model (CMTM4 WT vs. CMTM4 KO), Representative CMTM4, Ki67, CD44 IHC staining images of peritoneal tumor nodules in mice. The upper panels display images at low magnification (10×), while the lower panels present the corresponding regions at high magnification (40×). Scale bar: 50 μm.

(K) Body weight of the mice was measured every five days, and ascites formation was calculated as a percentage of weight gain. Data are presented as the mean ± SEM (n = 5 per group); unpaired two-sided Student's t-test (compared to CMTM4 KO group).

(L) WB verified CMTM4 overexpression in ID8 cells. Representative WB images were shown (n = 3 independent experiments per group).

(M-P) I.p. injected tumor model was created by injecting 2 × 10^6^ ID8 cells (ID8/OE-NC vs. ID8/OE-CMTM4) (n = 5 mice per group).

(M) When the ID8/OE-CMTM4-bearing mice reached the end point, all the mice were euthanized. After that, the blood ascites in the abdominal cavity of each group were collected for quantitative comparison. Data are presented as the mean ± SEM (n = 5); unpaired two-sided Student's t-test (compared to OE-NC group).

(N) Spleen images of 2 groups were presented, and the spleens were weighed to compare differences between groups. Data are presented as the means ± SEM (n = 5); unpaired two-sided Student's t-test (compared to OE-NC group).

(O) Metastatic nodules in peritoneum, omentum and mesentery were quantified. Data are presented as the mean ± SEM (n = 5); unpaired two-sided Student's t-test (compared to OE-NC group).

(P) In the i.p. tumor-bearing mouse model (OE-CMTM4 vs. OE-NC), Representative CMTM4, Ki67, CD44 IHC staining images of peritoneal tumor nodules in mice. The upper panels display images at low magnification (10×), while the lower panels present the corresponding regions at high magnification (40×). Scale bar: 50 μm.

All statistical analyses were using GraphPad Prism.

**Fig. S4**


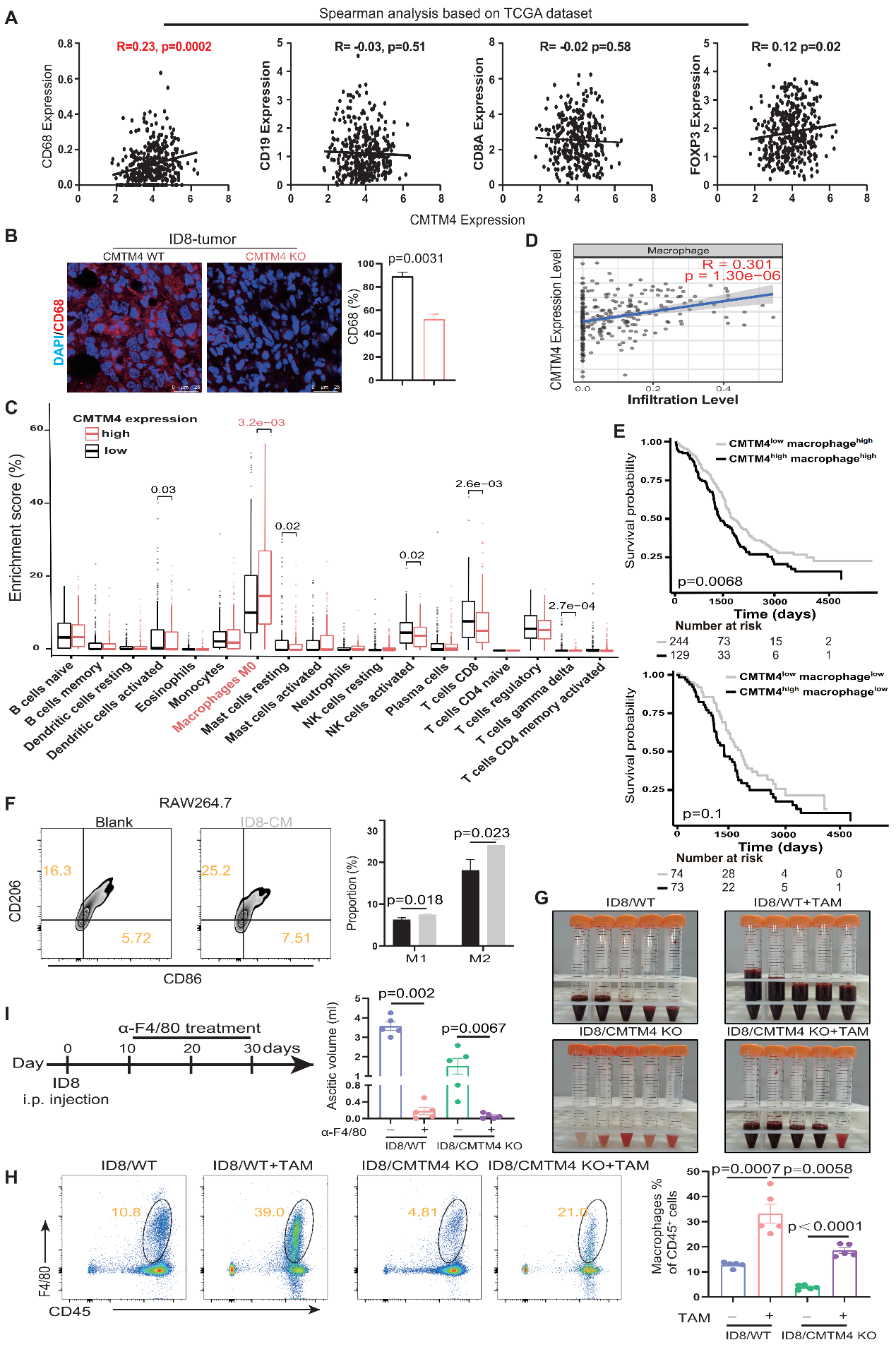


**Fig. S4** Macrophages play a crucial role in the tumorigenic effect of CMTM4.

(A) Association of expression levels of immune cell markers (CD68, CD19, CD8A, FOXP3) with CMTM4 expression in the TCGA-OC cohort.

(B) Fluorescent images of representative CD68^+^ macrophages in mouse tumor tissue (ID8/WT vs. ID8/CMTM4 KO). All panels are the same magnification. Scale bar, 25 μm. Data are presented as the mean ± SD (n = 3); unpaired two-sided Student's t-test (compared to CMTM4 KO group).

(C) Correlation between various immune cell enrichment scores and CMTM4 expression in TCGA-OC patients. Data are presented as the mean ± SEM. Statistical analysis was performed using the Wilcoxon rank-sum test.

(D) A TIMER database search confirmed the positive correlation between CMTM4 and macrophage infiltration.

(E) TCGA-OC data were classified into high macrophage/low macrophage infiltration groups. The CMTM4 expression was used as a marker to predict the OS of the cohort. Statistical analysis was performed using the log-rank test.

(F) RAW264.7 exposed to ID8-CM 48h was collected for FCM to determine the ratio of M1 (CD11b^+^CD86^+^) and M2 (CD11b^+^CD206^+^). Data are presented as the mean ± SD (n = 3); unpaired two-sided Student's t-test (compared to blank group).

(G) Images of malignant ascites in ID8-bearing mice under indicated treatment (n = 5 per group).

(H) The ratio of CD45^+^F4/80^+^ macrophages in hemorrhagic ascites of mice under indicated treatment. Data are presented as the mean ± SEM (n = 5); one-way ANOVA followed by Tukey’s multiple comparisons test.

(I) Schematic diagram of α-F4/80 treatment (left) (300 μg per mouse for three times). Quantitative analysis of ascites in mice with or without α-F4/80 treatment. Data are presented as the mean ± SEM (n = 5); unpaired two-sided Student's t-test.

All statistical analyses were using GraphPad Prism and FCM results were analyzed by FlowJo.

**Fig. S5**


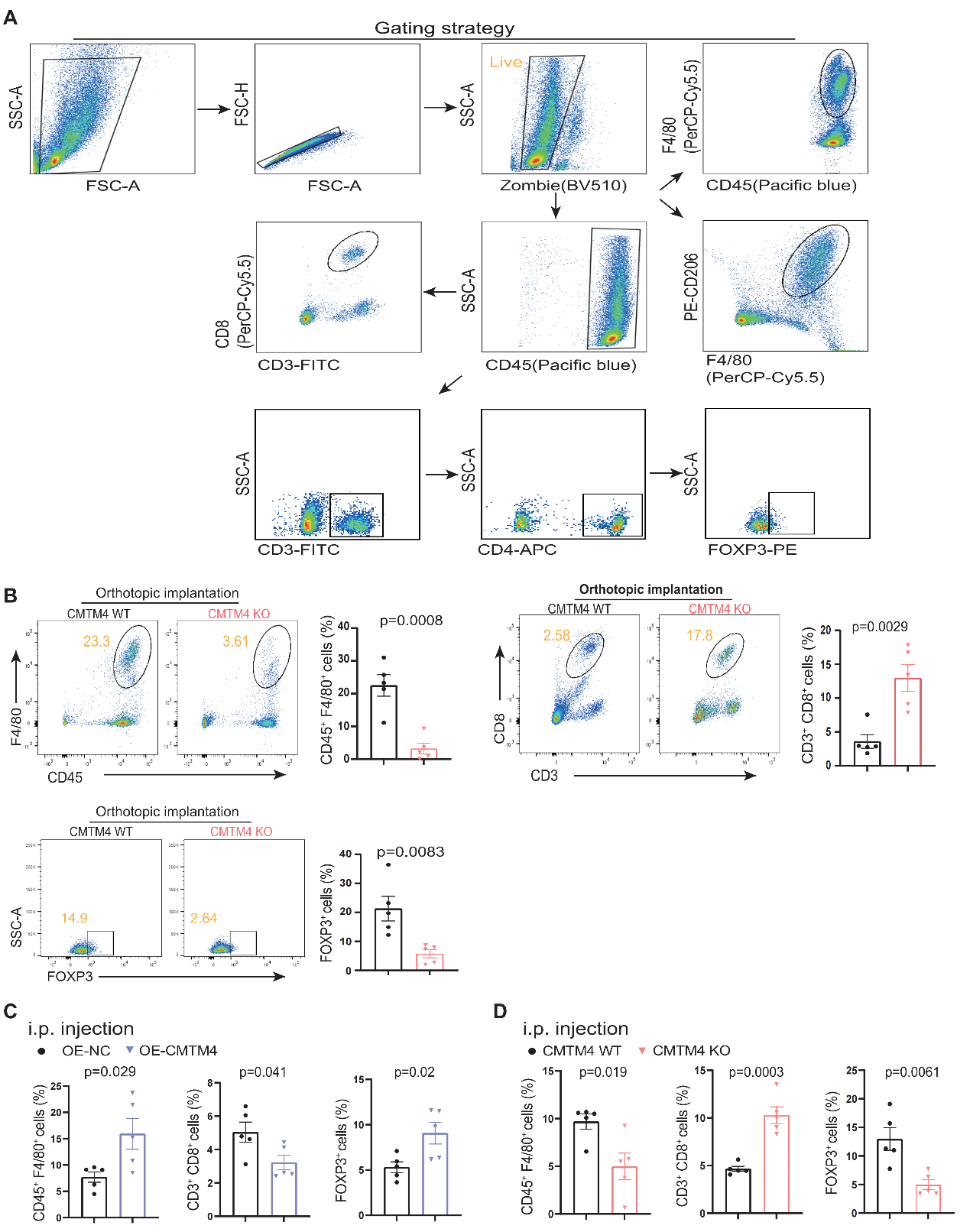


**Fig. S5** CMTM4 reshaped the immune microenvironment in OC.

(A) FCM gating strategy of immune cells. Following gating to live cells by Zombie fixable viability stain, infiltrating immune cells were gated for CD45^+^ cells. Macrophages were identified as CD45^+^F4/80^+^. CD8^+^ T cells were gated as CD45^+^CD3^+^CD8^+^ presented. Tregs were gated on CD45^+^CD3^+^CD4^+^ FOXP3^+^ cells presented.

(B) In an orthotopic ovarian tumor model, the proportions of macrophages, CD8^+^T cells, and Tregs in ascites were analyzed and quantified. Data are presented as the mean ± SEM (n = 5); unpaired two-sided Student's t-test (compared to CMTM4 KO group).

(C) In an i.p. OC model (ID8/OE-NC vs. ID8/OE-CMTM4), the proportions of macrophages, CD8^+^T cells, and Tregs in ascites were collected and quantified. Data are presented as the mean ± SEM (n = 5); unpaired two-sided Student's t-test (compared to OE-NC group).

(D) In an i.p. OC model (ID8/WT vs. ID8/CMTM4 KO), the proportions of macrophages, CD8^+^T cells, and Tregs in ascites were analyzed and quantified. Data are presented as the mean ± SEM (n = 5); unpaired two-sided Student's t-test (compared to CMTM4 KO group).

All statistical analyses were using GraphPad Prism and FCM results were analyzed by FlowJo.

**Fig. S6**


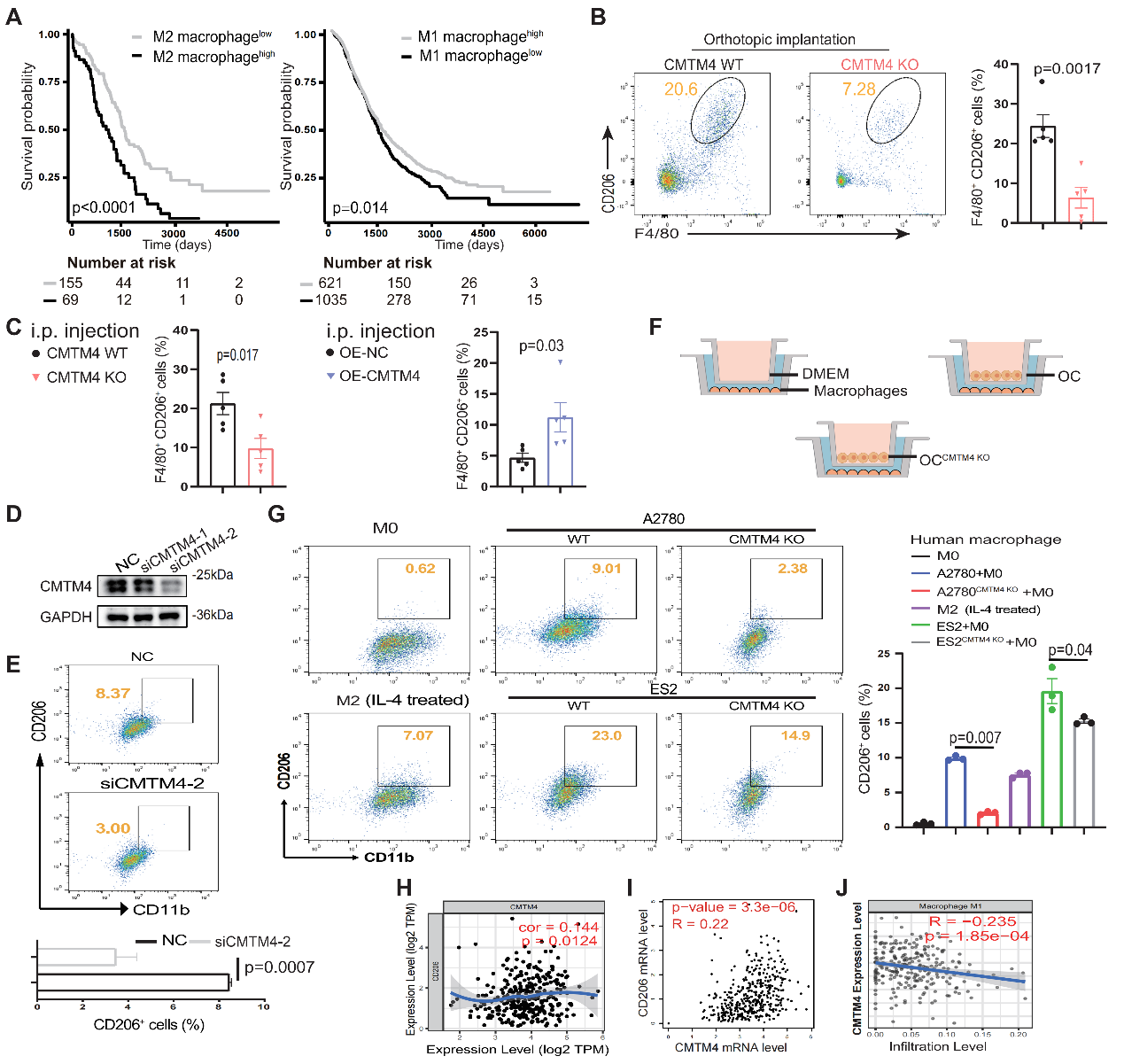


**Fig. S6** The presence of CMTM4 in OC supports the maintenance of M2 macrophages.

(A) The impact of the M2 (CD206) and M1 (CD86) macrophages infiltration ratio on OS was analyzed in the TCGA-OC cohort using log-rank tests.

(B-C) FCM was used to analyze the proportion of M2 macrophages in the hemorrhagic ascites produced by the mouse tumor in situ model and the peritoneal tumor bearing model.

(B) Analysis of M2 macrophages in hemorrhagic ascites of mice orthotopically implanted ID8 cells (ID8/WT vs. ID8/CMTM4 KO, n = 5 mice per group). Data are presented as the mean ± SEM; unpaired two-sided Student's t-test.

(C) Investigation of M2 macrophage profiles in hemorrhagic ascites in mice Intraperitoneally injection ID8 cells (ID8/WT vs. ID8/CMTM4 KO, ID8/OE-NC vs. ID8/OE-CMTM4, n = 5 mice per group). Data are presented as the mean ± SEM; unpaired two-sided Student's t-test.

(D) WB verified the knockdown efficiency of CMTM4 siRNA. Representative WB images are shown (n = 3 independent experiments per group).

(E) FCM was used to measure the proportion of CD206^+^ macrophages at indicated treatment. Representative results are shown. Data are presented as the mean ± SD （n = 3 independent experiments per group）; unpaired two-sided Student's t-test,

(F) Simple model of co-culture of tumor cells and macrophages, macrophages (placed in the lower chamber of co-culture) were cocultured with DMEM or OC cell lines (A2780, ES2) with or without CMTM4-KO for 48 h.

(G) FCM assays the proportion of macrophages with CD206 under specified education. The representative results were shown, data are presented as the mean ± SD (n = 3 independent experiments per group); unpaired two-sided Student's t-test.

(H-I) The positive relationship between CMTM4 and CD206 expression was examined using TIMER (H) and GEPIA (I) database.

(J) The negative association between CMTM4 expression and M1 macrophage infiltration in the TME was analyzed by TIMER database.

All statistical analysis were using GraphPad Prism and FCM results were analyzed by FlowJo.

**Fig. S7**


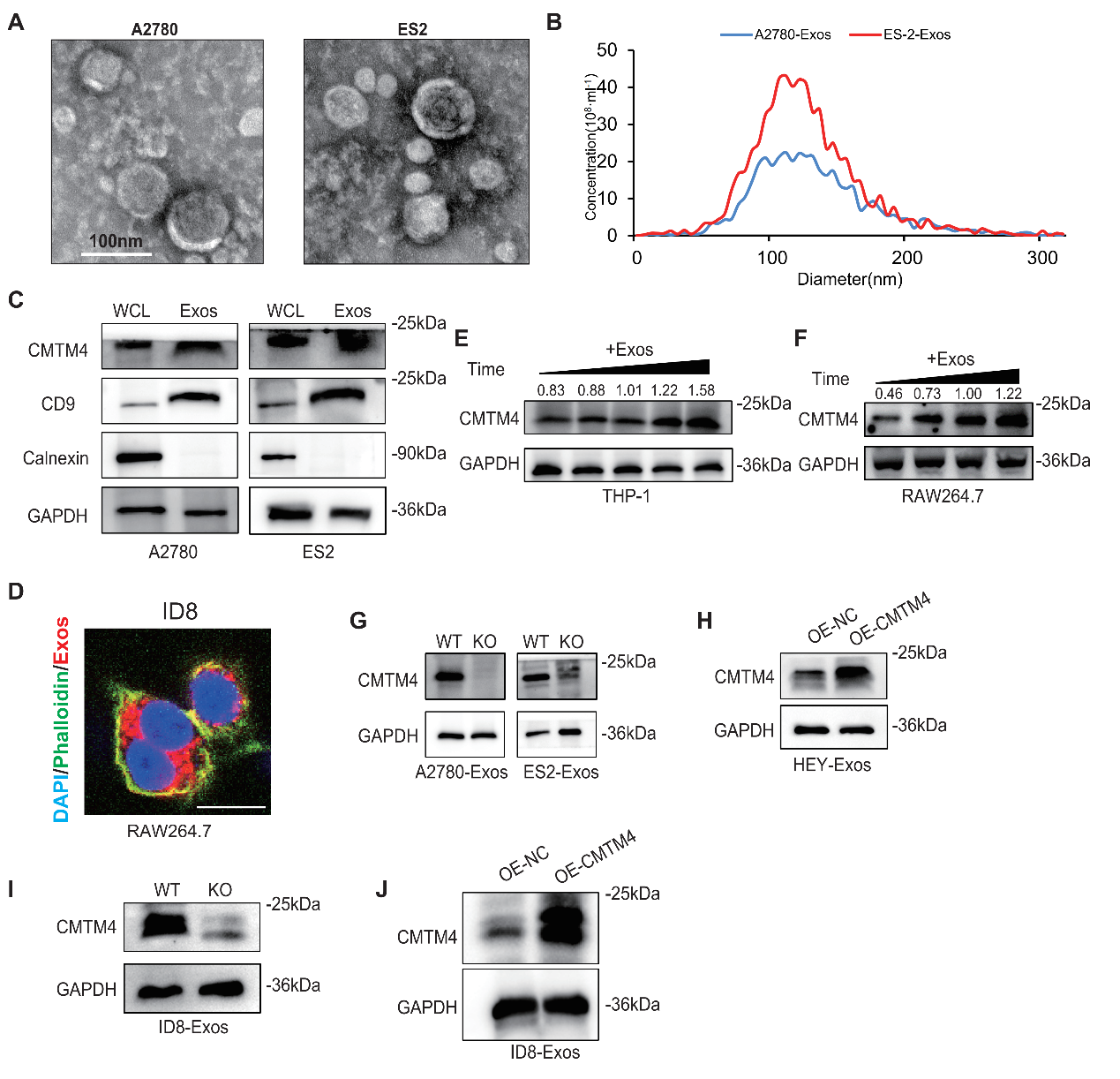


**Fig. S7** CMTM4 protein is encapsulated in the exosomes of OC cells.

(A-B) Phenotypic analysis of exosomes derived from A2780 and ES2 cells was conducted using TEM (A) and NTA (B). Scale bar: 100 nm. The curve graph was generated using Microsoft Excel.

(C) Exosomes were characterized by WB and CMTM4 levels in tumor whole cell lysate (WCL) and cell-secreted exosomes were analyzed.

(D) Fluorescent image illustrating the uptake of ID8 cell-derived exosomes by RAW264.7. Scale bar, 25 μm.

(E) Exosomes derived from human cell lines were cultured with macrophages for 0, 4, 16, 24, and 48 h, and the expression of CMTM4 was assessed by WB.

(F) Exosomes from the ID8 cell line were incubated with RAW264.7 for 0, 4, 24, and 48 h, and CMTM4 expression was evaluated by WB.

(G) The expression of CMTM4 in exosomes originated from the cell line with downregulated CMTM4 expression was assessed via WB.

(H) The expression of CMTM4 in the exosomes was evaluated by WB after the upregulation of CMTM4 expression in the cell line.

(I-J) WB was utilized to evaluate the changes of CMTM4 expression in exosomes derived from ID8 after the change of CMTM4 expression.

**Fig. S8**


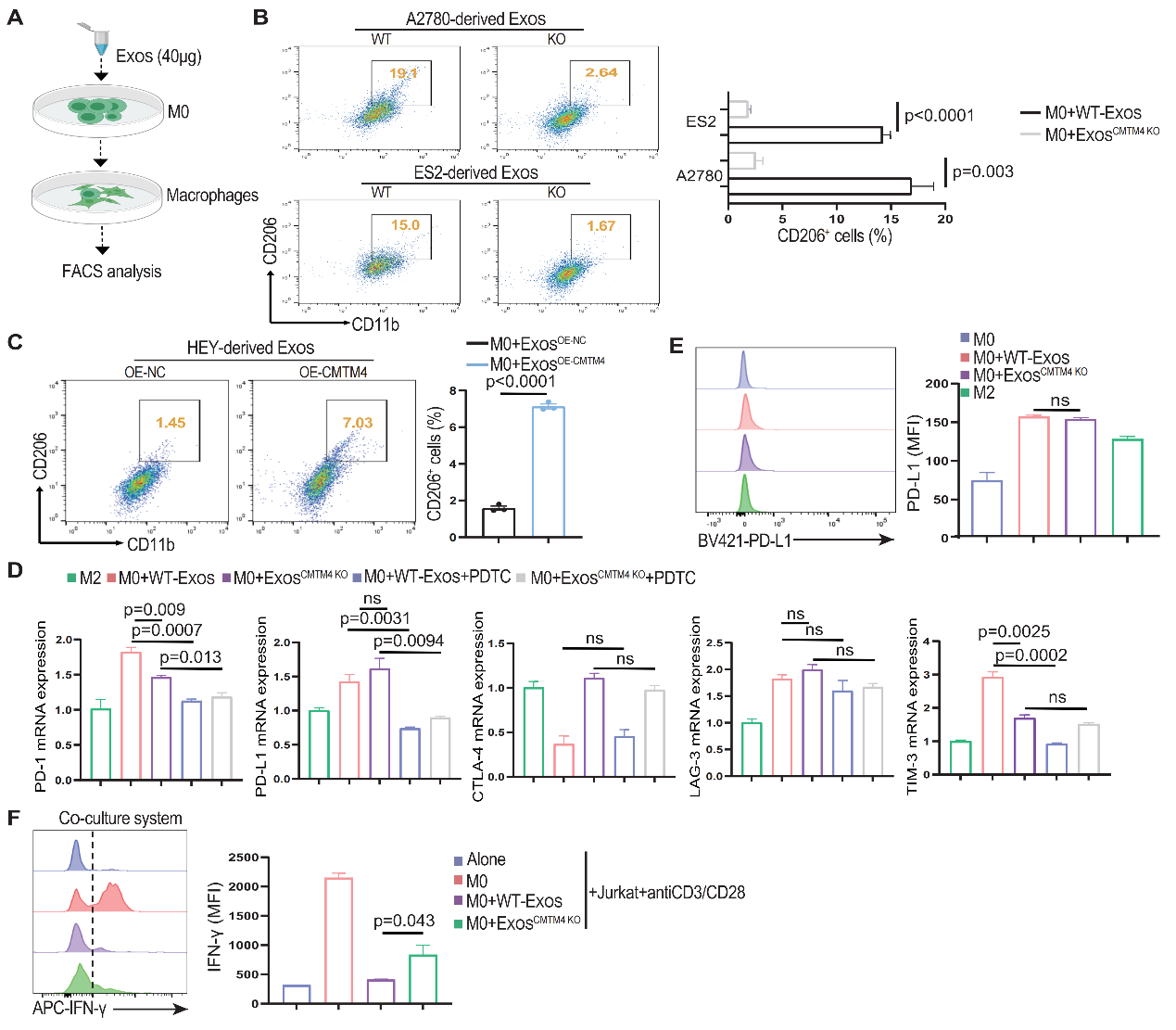


**Fig. S8** Exosome-mediated CMTM4 induction promotes the formation of immunosuppressive macrophages.

(A) Schematic illustration of co-culture between macrophages and tumor cell-derived exosomes.

(B) FCM was used to assess the CD206 level (M2) on THP-1-derived macrophages treated with different exosome (WT-Exos vs. Exos^CMTM4 KO^) preparations. Representative results are shown. Data are presented as the mean ± SD (n = 3 independent experiments per group); unpaired two-sided Student's t-test.

(C) CD206 expression in macrophages treated with exosomes (Exos-OE-NC vs. Exos-OE-CMTM4) was measured by FCM. Representative results are shown. Data are presented as the mean ± SD (n = 3 independent experiments per group); unpaired two-sided Student's t-test.

(D) Detection of mRNA levels of *PD-1, PD-L1, CTLA-4, LAG-3, and TIM-3* in macrophages under specified treatment conditions. Data are presented as the mean ± SD (n = 3 independent experiments per group); one-way ANOVA followed by Tukey’s multiple comparisons test,

(E) FCM was employed to assess PD-L1 levels on macrophages cocultured with exosomes from OC cell line (A2780) control or CMTM4 KO for 24 h. data are presented as the mean ± SD (n = 3 independent experiments per group); unpaired two-sided Student's t-test.

(F) Macrophage treated with exosomes (WT-Exos vs. Exos^CMTM4 KO^) and Jurkat (activated by anti- CD3/CD28) co-culture and Jurkat T cells were collected for FCM analysis to assess IFN-γ across different groups. Data are presented as the mean ± SD (n = 3 independent experiments per group); unpaired two-sided Student's t-test. MFI was analyzed using FlowJo.

All statistical analyses were using GraphPad Prism and FCM results were analyzed by FlowJo.

**Fig. S9**


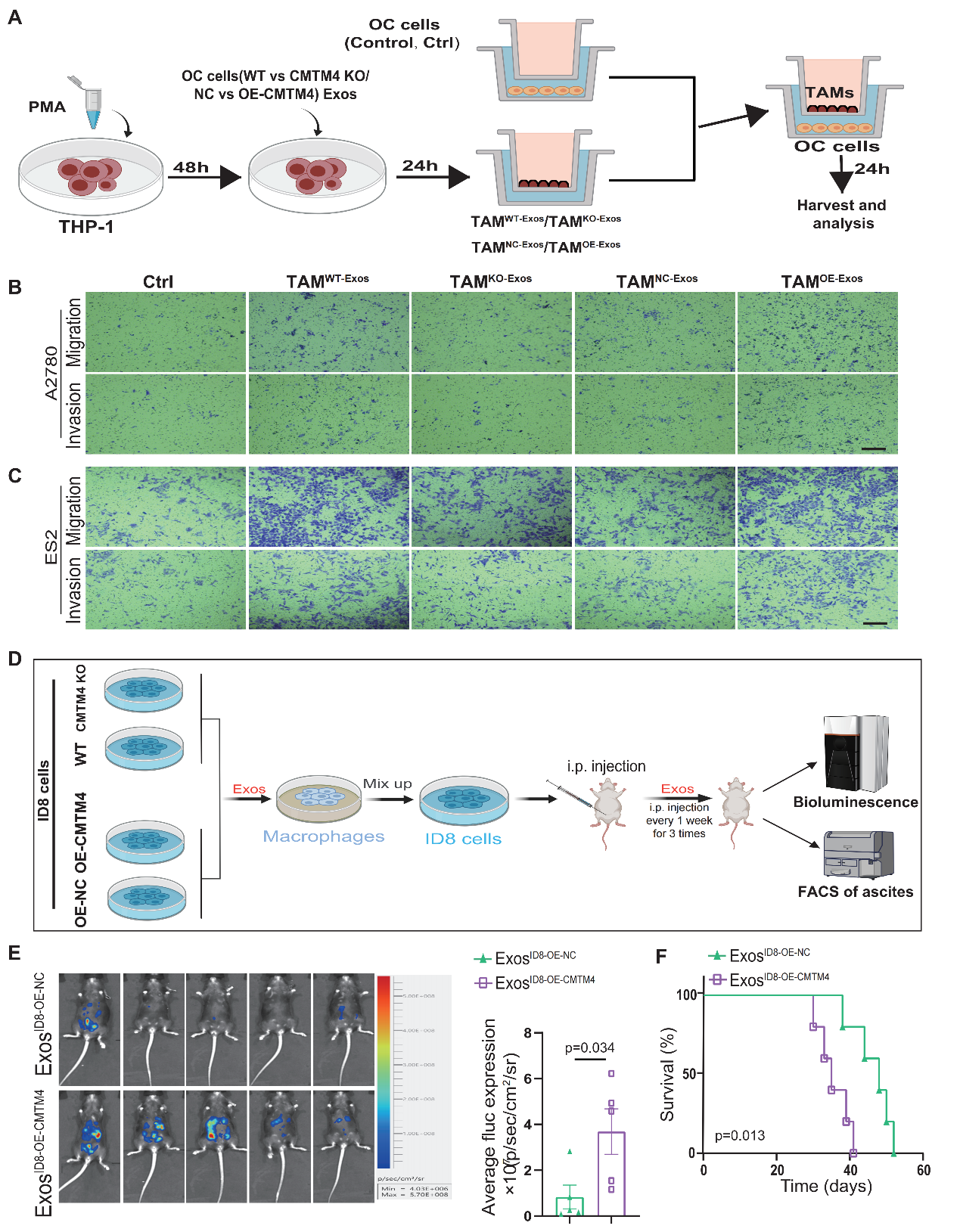


**Fig. S9** Exosomal CMTM4 drives macrophage-mediated OC aggressiveness in vitro and in vivo.

(A) THP-1 cells were initially treated with PMA, followed by exposure to medium or exosomes from tumor cells (WT vs. CMTM4 KO/NC vs. OE-CMTM4) to generate TAMs. These TAMs were then co-cultured with tumor cells for the subsequent experiments.

(B-C) OC cell lines were co-cultured with macrophages under different treatment conditions for 24 h, followed by a Transwell assay to assess A2780 and ES2 migratory and invasive capacities. Representative images of migration and invasion of A2780 (B) and ES2 (C) cells co-cultured with TAMs. All panels are the same magnification. Scale bar, 100 μm, n=3 independent experiments per group.

(D) Mechanism map of in vivo exosome injection and tumorigenicity test. ID8 cells and macrophages (RAW264.7) treated with ID8 cell-derived exosomes (WT-Exos vs. KO-Exos, OE-Exos vs. NC-Exos) were co-injected into the mice’s abdominal cavity at a 10:1 ratio. FCM analysis of immune cells from mouse ascites and in vivo bioluminescence imaging were performed on day 30 to detect tumor growth.

(E) 2 × 10^6^ ID8 cells and 2 × 10^5^ (ratio 10:1) macrophages specified treatments were co-injected into mice, and in vivo bioluminescence imaging was performed on day 30 (n = 5 mice per group). Data are presented as the mean ± SEM; unpaired two-sided Student's t-test.

(F) Survival analysis of mice co-injected with ID8 cells and macrophages under specific treatments was performed using the log-rank test (n = 5 mice per group).

All statistical analyses were using GraphPad Prism.

**Fig. S10**


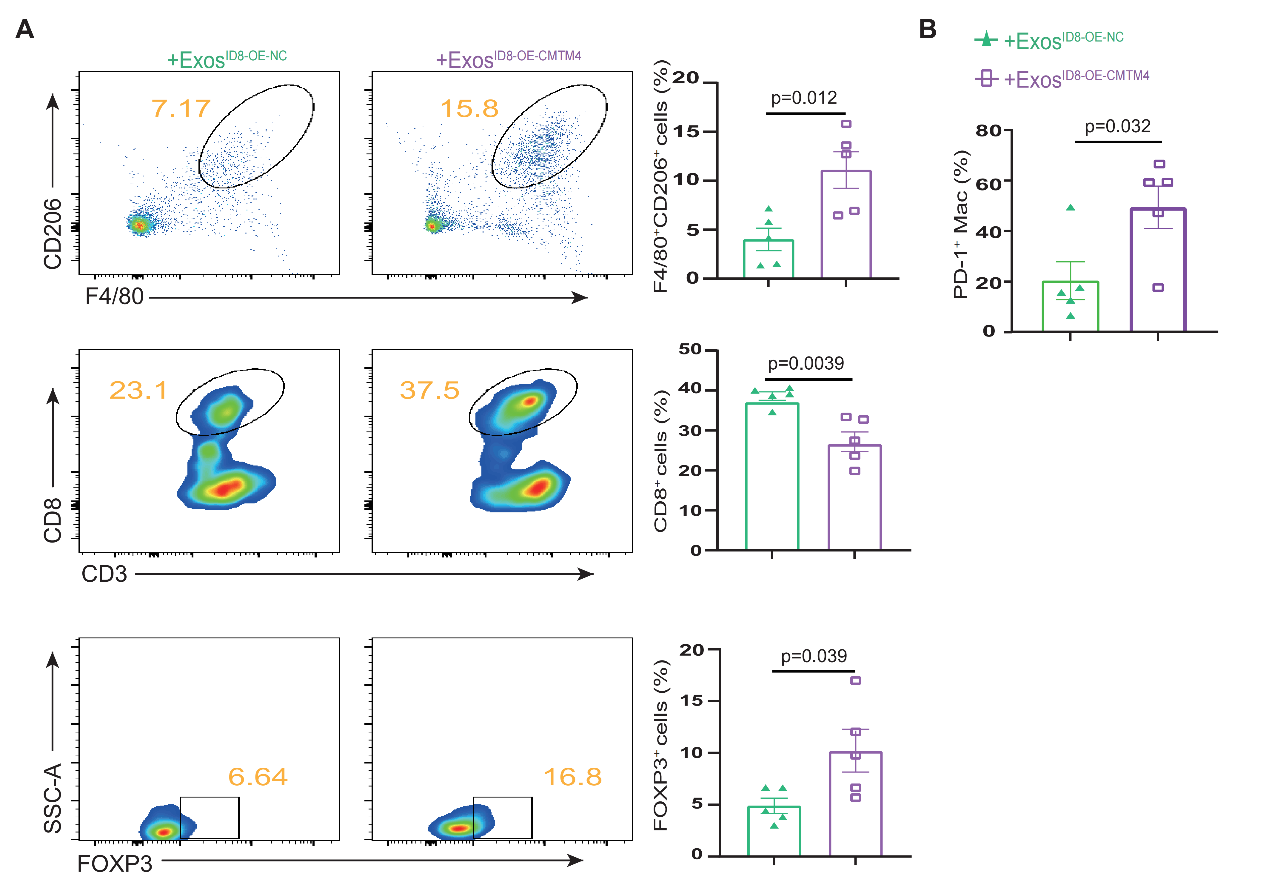


**Fig. S10** Exosomal CMTM4 drives macrophage-mediated immunosuppression in vivo.

(A) FCM analysis the percentage of immune cells (CD45^+^F4/80^+^CD206^+^ macrophages, CD45^+^CD3^+^CD8^+^ T cells, CD45^+^CD3^+^CD4^+^FOXP3^+^ Tregs) of mouse ascites in two groups (n = 5 per group). Representative FCM images are shown. Data are presented as the mean ± SEM; unpaired two-sided Student's t-test.

(B) FCM analysis the percentage of CD45^+^F4/80^+^CD206^+^PD-1^+^ macrophages of mouse ascites in two groups (n=5 per group). Data are presented as the mean ± SEM; unpaired two-sided Student's t-test.

All statistical analyses were using GraphPad Prism and FCM results were analyzed by FlowJo.

**Fig. S11**


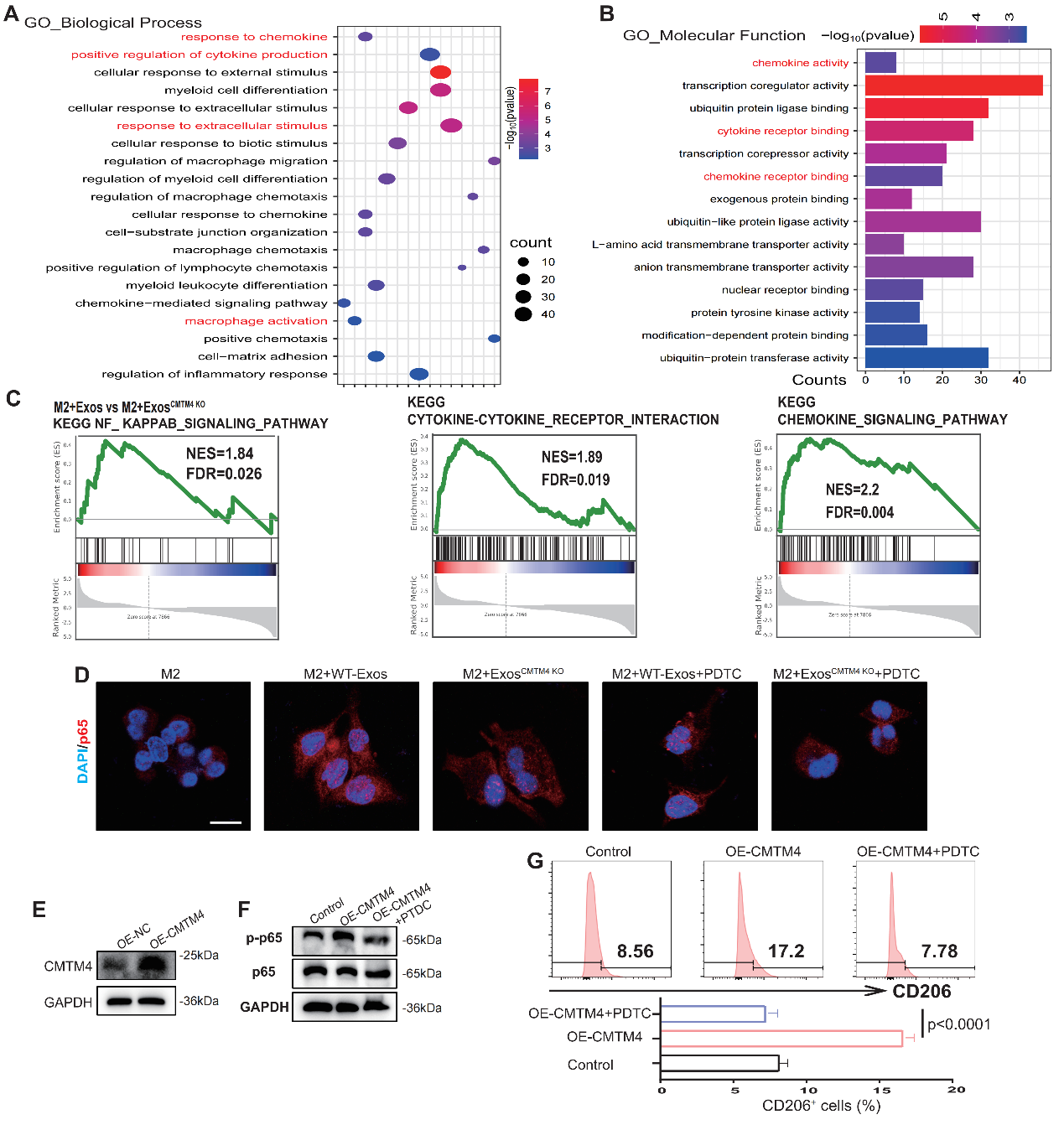


**Fig. S11** CMTM4 regulates M2 macrophage polarization by activating the NF-KB pathway.

(A) Bubble plot showing the GO (Gene Ontology-biological process) analysis of shared DEGs.

(B) The bar chart illustrating the GO-Molecular Function analysis of shared DEGs.

(C) GSEA for NF-KAPPAB signaling pathway, Cytokine-cytokine receptor interaction and chemokine signaling pathway of DEGs (M2+Exos vs. M2+Exos^CMTM4 KO^).

(D) M2 macrophages were treated by OC cell lines‐derived exosomes with or without CMTM4 for 12h, with or without pretreatment of PDTC for 12h. IF was applied for assessing the translocation of p65 in M2 macrophages. All panels are the same magnification. Scale bar, 25 μm.

(E) The efficiency of CMTM4 overexpression was verified by WB in macrophages. Representative WB images are shown, n = 3 independent experiments per group.

(F) M2 Macrophages were treated by CMTM4 overexpression or control, with or without PDTC. WB analysis to detect p65 and p-p65 in macrophages. Representative WB images are shown, n = 3 independent experiments per group.

(G) FCM analysis for CD206 levels in treated M2 macrophages. Representative images are shown. Data are presented as the mean ± SEM (n=3 independent experiments per group); one-way ANOVA followed by Tukey’s multiple comparisons test.

All statistical analyses were using GraphPad Prism and FCM results were analyzed by FlowJo.

**Fig. S12**


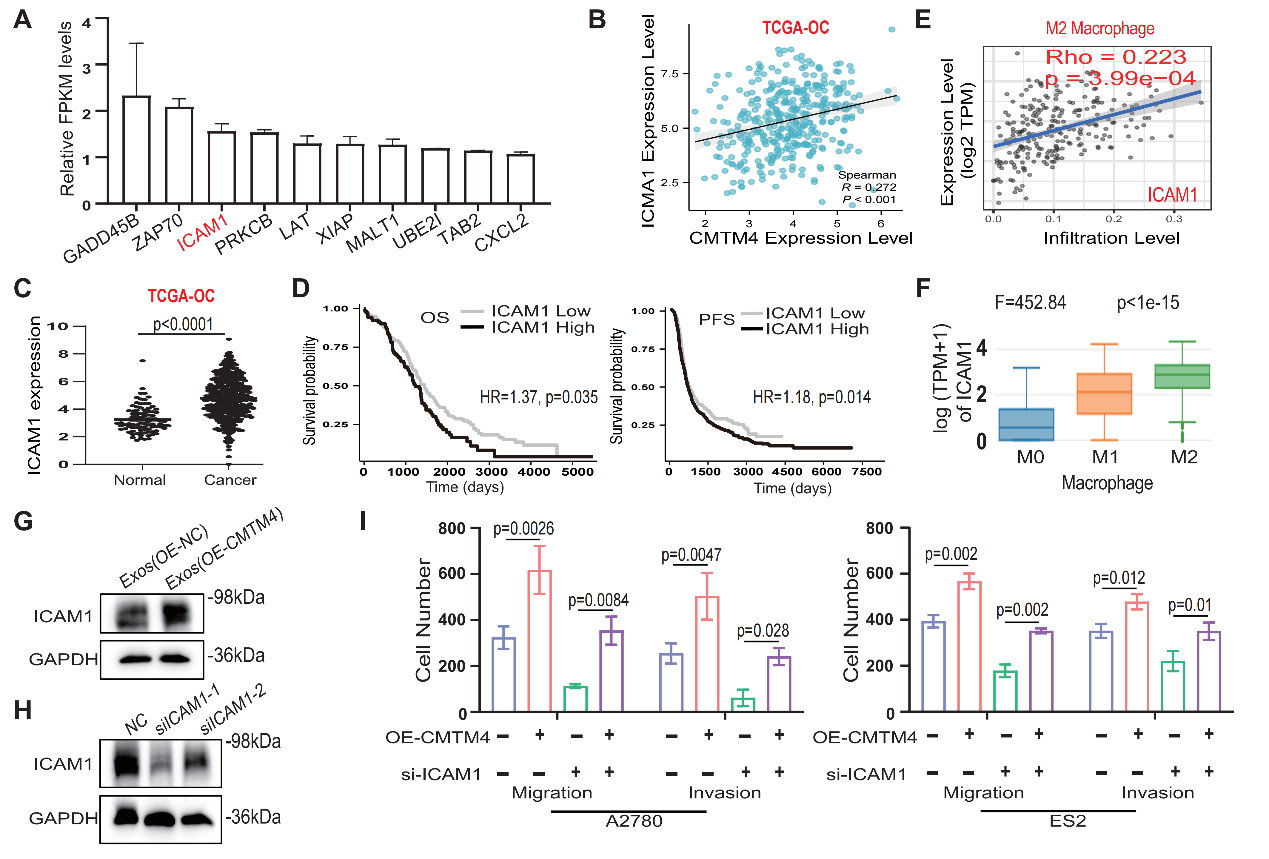


**Fig. S12** Exosomal CMTM4 promotes OC progression by regulating M2 macrophage polarization through ICAM1.

(A) Relative FPKM values of the top ten differential genes of NF-κB signaling pathway in M2+Exos groups (RNA-seq). Data are shown as relative to the M2+Exos^CMTM4 KO^ group.

(B) Correlation analysis between CMTM4 and ICAM1 expression based on TCGA-OC cohort (R=0.272; p<0.001).

(C) The expression of ICAM1 in normal and cancer tissues in the TCGA-OC cohort was analyzed. Data are presented as the mean ± SEM; unpaired two-sided Student's t-test.

(D) The Kaplan–Meier survival curves were drawn to state the relationship between ICAM1 expression and OS (left) and PFS (right) in OC patients based TCGA. Analyzed using the log-rank test.

(E) Correlation analysis for ICAM1 and M2 macrophage infiltration by TIMER databases.

(F) Expression of ICAM1 in M0, M1, and M2 macrophages using GEPIA database.

(G) WB analysis for ICAM1 in M2 macrophages treated with exosomes derived from HEY cell line. Representative WB images are shown, n=3 independent experiments per group.

(H) The efficiency of ICAM1-siRNA was confirmed by WB in macrophages. Representative WB images are shown, n=3 independent experiments per group.

(I) The number of migrating and invading cells of A2780 (left) and ES2 (right) were quantitatively analyzed by ImageJ software after co-culture with designated treated macrophages. Data are presented as the mean ± SD (n=3); one-way ANOVA followed by Tukey’s multiple comparisons test.

All statistical analyses were using GraphPad Prism.

**Fig. S13**


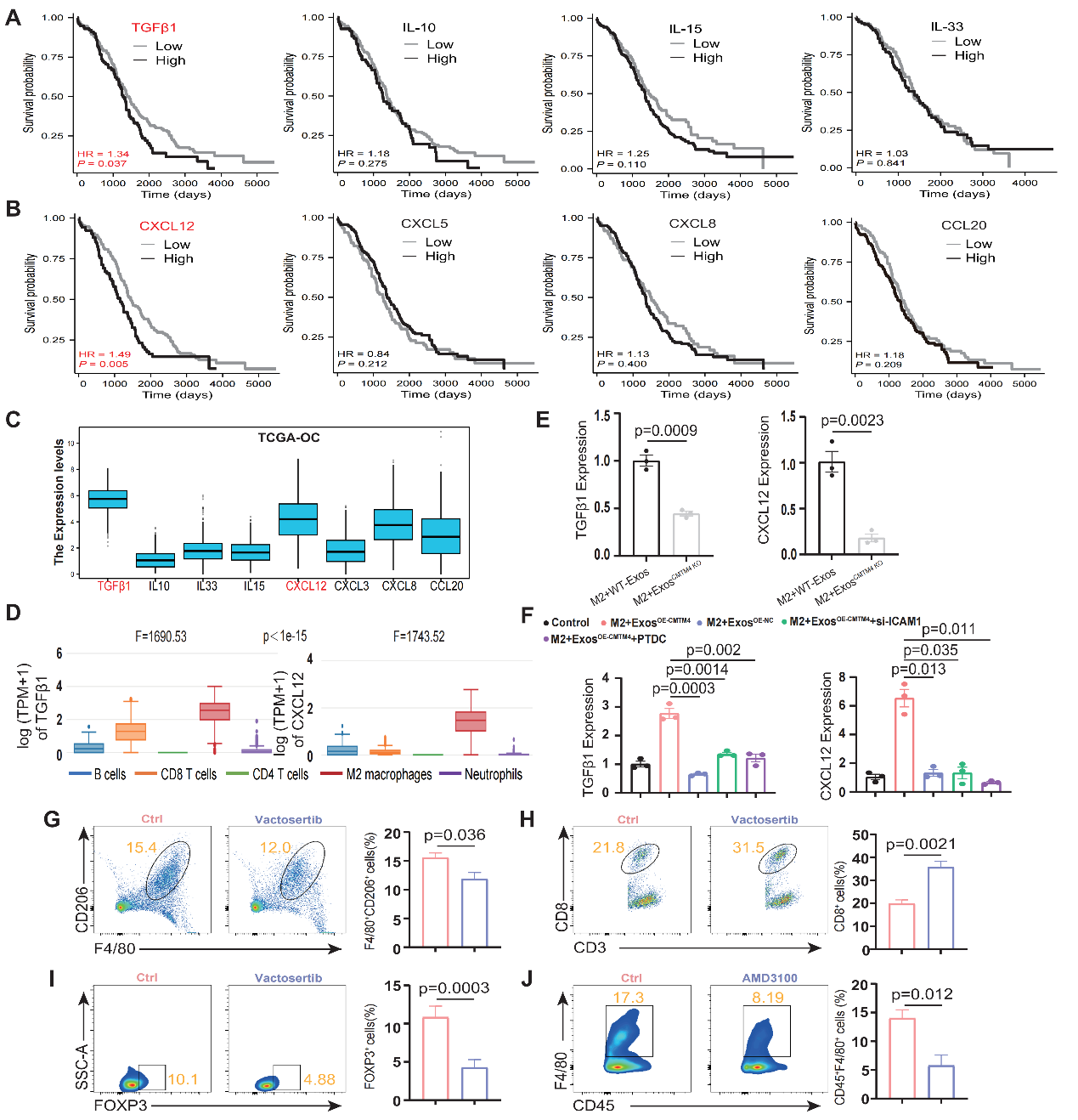


**Fig. S13** Exosomal CMTM4 stimulates macrophages to increase cytokine and chemokine production.

(A) Kaplan–Meier survival curves were generated to illustrate the relationship between inflammatory factors (TGF-β1, IL-10, IL-15, IL-33) levels and OS in OC patients from TCGA. Statistical analysis was performed using the log-rank test.

(B) Kaplan–Meier survival curves were constructed to demonstrate the association between chemokine (CXCL12, CXCL5, CXCL8, CCL20) levels and OS in OC patients from TCGA. Statistical analysis was performed using the log-rank test.

(C) Comparison of expression levels of cytokines or chemokines (TGF-β1, IL-10, IL-15, IL-33, CXCL12, CXCL5, CXCL8, CCL20) in TCGA-OC cohort. Data are presented as the mean ± SEM.

(D) Expression of TGF-β1 and CXCL12 in B cells, CD8^+^ T cells, CD4^+^ T cells, M2 macrophages, and neutrophils using GEPIA database.

(E) qPCR was used to verify elevated *TGF-β1* and *CXCL12* mRNA levels in macrophages treated with exosomal CMTM4. Data are presented as the mean± SD (n = 3); unpaired two-sided Student's t-test.

(F) The concentrations of TGF-β1 and CXCL12 in the supernatant of M2 macrophages after treated with Exos^OE-CMTM4^, Exos^OE-NC,^ Exos^OE-CMTM4^+si-ICAM1, Exos^OE-CMTM4^+PDTC were measured by qPCR. Data are presented as the mean ± SD (n = 3); unpaired two-sided Student's t-test.

(G-I) FCM analysis the percentage of immune cells (CD45^+^F4/80^+^CD206^+^ macrophages, CD45^+^CD3^+^CD8^+^ T cells, CD45^+^CD3^+^CD4^+^FOXP3^+^ Tregs) of mouse ascites from both the control and vactosertib groups (n = 4). Data are presented as the mean ± SD; unpaired two-sided Student's t-test.

(J) FCM analysis the percentage of CD45^+^F4/80^+^ macrophages of mouse ascites from both the control and AMD3100-treated groups (n = 4). Data are presented as the mean ± SD; unpaired two-sided Student's t-test.

All statistical analyses were using GraphPad Prism and FCM results were analyzed by FlowJo.

**Fig. S14**


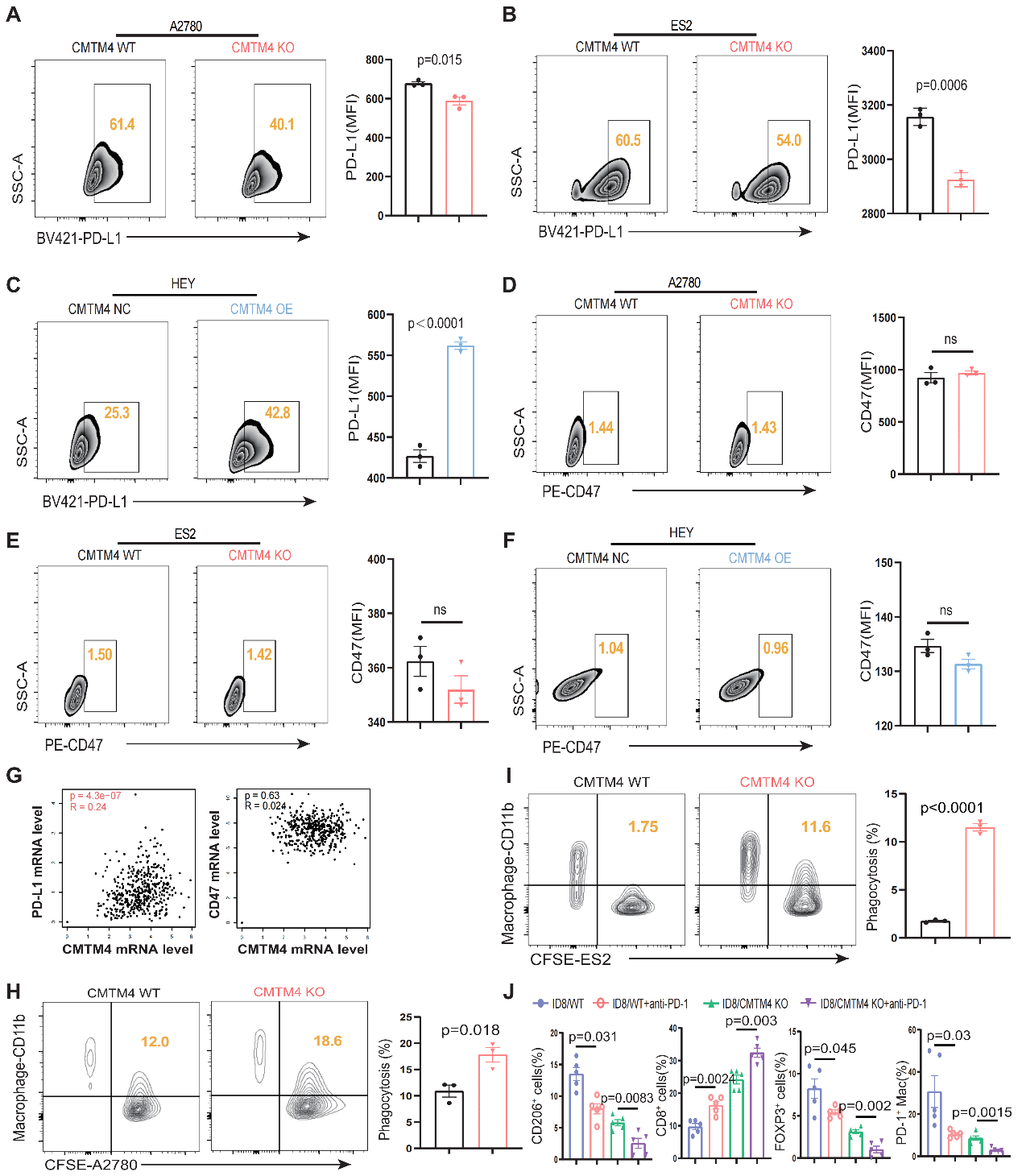


**Fig. S14** CMTM4-mediated PD-L1 expression in OC enables immune evasion by macrophages.

(A-B) The expression of PD-L1 in A2780 (A) and ES2 (B) cells as indicated group (CMTM4-WT vs. CMTM4 KO) was detected by FCM. Data are presented as the mean ± SD (n = 3); unpaired two-sided Student's t-test.

(C) FCM was used to detect the expression of PD-L1 after CMTM4 overexpression in HEY cells. Data are presented as the mean ± SD (n = 3); unpaired two-sided Student's t-test.

(D-F) FCM was used to assess the expression of CD47 in A2780 (D), ES2 (E), and HEY (F) cells across different CMTM4 expression groups. Data are presented as the mean ± SD (n = 3); unpaired two-sided Student's t-test.

(G) The relationship between CMTM4 and the expression of PD-L1 (left) and CD47 (right) was analyzed using the GEPIA database.

(H-I) CFSE-labeled tumor cells were cocultured with CD11b-labeled macrophages for 4 h. FCM was used to assess tumor cell (A2780, ES2) phagocytosis by macrophages in each group. Data are presented as the mean ± SD (n=3); unpaired two-sided Student's t-test.

(J) FCM analysis the percentage of immune cells (CD45^+^F4/80^+^CD206^+^ macrophages, CD45^+^CD3^+^CD8^+^ T cells, CD45^+^CD3^+^CD4^+^FOXP3^+^ Tregs, CD45^+^F4/80^+^CD206^+^PD-1^+^ macrophages) of mouse ascites between groups (n = 5 mice per group). Data are presented as the mean ± SD; unpaired two-sided Student's t-test.

The FCM results (A-F) were quantified using MFI values. Representative images are displayed. All statistical analyses were using GraphPad Prism and FCM results were analyzed by FlowJo.

**Fig. S15**
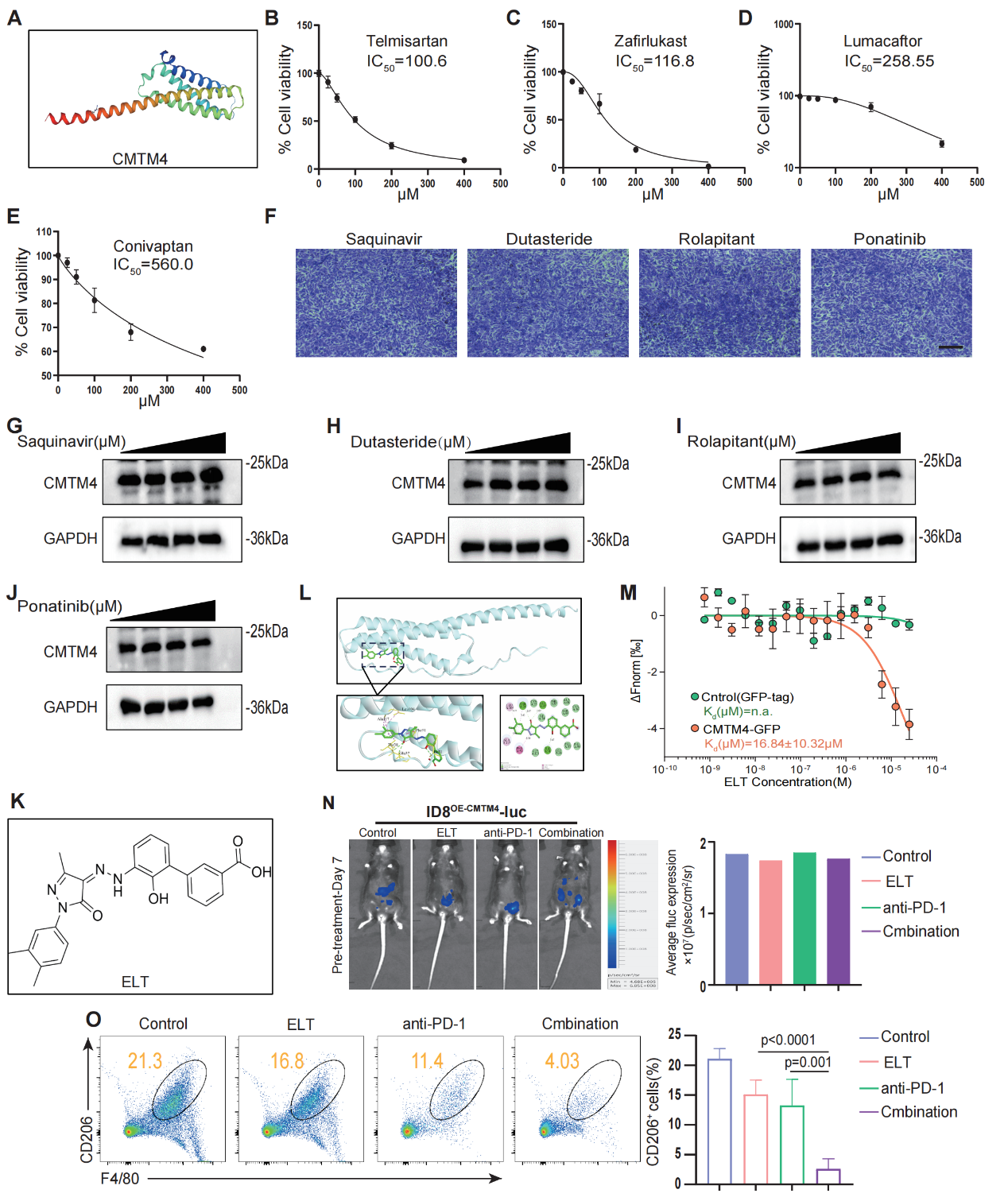


**Fig. S15** Screening of small-molecule inhibitors of CMTM4.

(A) The protein structure of CMTM4 was predicted using AlphaFold.

(B-E) CCK-8 measures the IC_50_ value of telmisartan(B), zafirlukast(C), lumacaftor(D), and conivaptan(E) against ID8 cells for 48 h. Cell viability without drug treatment was defined as 100%. Data represent n = 3 independent experiments per group.

(F) Representative images of ID8 cell migration after treatment with saquinavir, dutasteride, rolapitant and ponatinib for 48 h. All panels are the same magnification. Scale bar, 100 μm.

(G-J) WB was used to detect CMTM4 expression in ID8 cells treated with increasing concentrations of saquinavir (F), dutasteride (G), rolapitant (H), and ponatinib (I). Representative WB images are shown (n = 3 independent experiments per group).

(K) ELT chemical structure was exhibited from MedChemExpress company (<https://www.medchemexpress.cn/>).

(L) Autodock predicts molecular docking of CMTM4 with ELT.

(M) MST analysis of the interaction between ELT and CMTM4. The K_d_ value for GFP-CMTM4 was determined to be 16.84 ± 10.32 μM, indicating a moderate binding affinity. In contrast, no detectable interaction was observed with GFP-tag, and the K_d_ value was not available (n.a.). N = 3 independent experiments per group. The curves were plotted using GraphPad Prism.

(N) Mice received an i.p. injection of ID8^OE-CMTM4^-luc cells (3 × 10^6^) and followed by in vivo bioluminescence imaging. In vivo bioluminescence imaging was performed on day 7 to assess baseline tumor burden. Data are presented as the mean.

(O) FCM was employed to analyze the proportion of M2 macrophages (CD45^+^F4/80^+^CD206^+^) in the ascites of mice (n = 5 mice per group) under specific treatment conditions. Data are presented as the mean ± SEM. Statistical analysis was performed using one-way ANOVA followed by Tukey’s multiple comparisons test.

All statistical analyses were using GraphPad Prism and FCM results were analyzed by FlowJo.

**Fig. S16**


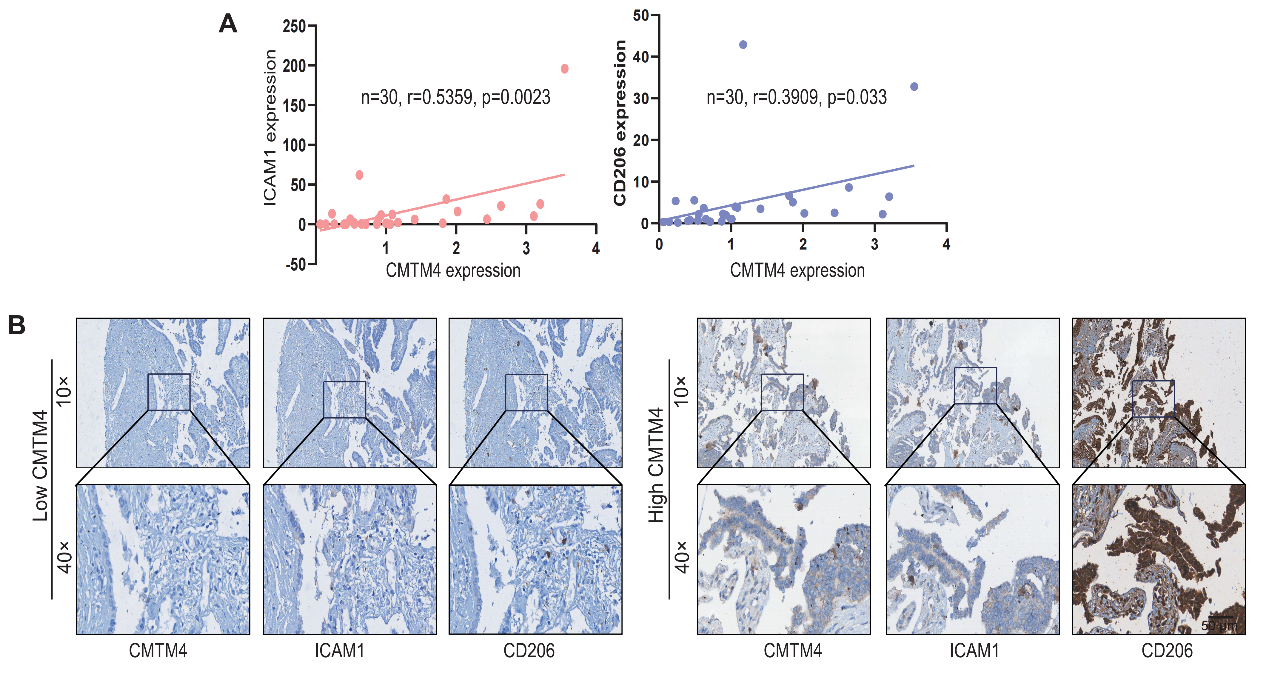


**Fig. S16** Elevated CMTM4 expression correlates with increased M2 macrophage infiltration and ICAM1 expression.

(A) The qPCR was performed to detect the expression of *CMTM4*, *ICAM1*, and *CD206* in OC tissues and analyzed their correlation (n = 30).

(B) IHC results showed that ICAM1 and CD206 were highly expressed in the CMTM4 high-expression region and vice versa in OC tissues. The upper panels display images at low magnification (10×), while the lower panels present the corresponding regions at high magnification (40×). Scale bar: 50 μm.

All statistical analyses were using GraphPad Prism.

**Reference**

1. Newman AM, Liu CL, Green MR, Gentles AJ, Feng W, Xu Y, Hoang CD, Diehn M, Alizadeh AA. Robust enumeration of cell subsets from tissue expression profiles. Nat Methods. 2015; 12: 453-7.

2. Tang Z, Li C, Kang B, Gao G, Li C, Zhang Z. GEPIA: a web server for cancer and normal gene expression profiling and interactive analyses. Nucleic Acids Res. 2017; 45: W98-W102.

3. Gyorffy B. Integrated analysis of public datasets for the discovery and validation of survival-associated genes in solid tumors. Innovation (Camb). 2024; 5: 100625.

4. Li T, Fu J, Zeng Z, Cohen D, Li J, Chen Q, Li B, Liu XS. TIMER2.0 for analysis of tumor-infiltrating immune cells. Nucleic Acids Res. 2020; 48: W509-W14.

5. UniProt C. UniProt: the Universal Protein Knowledgebase in 2023. Nucleic Acids Res. 2023; 51: D523-D31.

6. Li N, Wang B, Yang M, Feng M, Xu X, Xian CJ, Li T, Zhai Y. The Multi-Target Action Mechanism for the Anti-Periodontitis Effect of Astragali radix Based on Bioinformatics Analysis and In Vitro Verification. Nutrients. 2025; 17.

7. Irwin JJ, Tang KG, Young J, Dandarchuluun C, Wong BR, Khurelbaatar M, Moroz YS, Mayfield J, Sayle RA. ZINC20-A Free Ultralarge-Scale Chemical Database for Ligand Discovery. J Chem Inf Model. 2020; 60: 6065-73.

8. Trott O, Olson AJ. AutoDock Vina: improving the speed and accuracy of docking with a new scoring function, efficient optimization, and multithreading. J Comput Chem. 2010; 31: 455-61.
